# Dataset from Code-switching between English and Malay Languages in Malaysian Premier Polytechnics ESL Classrooms

**DOI:** 10.1016/j.dib.2022.108709

**Published:** 2022-10-29

**Authors:** Mazlin Mohamed Mokhtar, Maizatulliza Muhamad, Aireen Aina Bahari, Farah Natchiar Mohd. Khaja

**Affiliations:** Sultan Idris Education University, 35900 Tanjung Malim, Perak

**Keywords:** SLA (Second Language Acquisition), Bilingualism, ME (Malaysia English), ESP (English for Specific Purposes)

## Abstract

The data was collected using a mixed method study of convergent parallel design, conducted using classroom observations, interviews and questionnaires to triangulate the data obtained from the three Premier Polytechnics in Malaysia, which involved nine lecturers and 183 students. The data is useful in focussing on the structure that normally occurs whenever code-switching happens and to test on how effective it is in the learning of English. Further research could also be based on these data as to identify the potential functions of code-switching and its contribution towards the language policy in Malaysia as reference to other countries too. It will provide an understanding of patterns and reasons for code-switching and subsequently offer insights into the use of code-switching as an effective language teaching and learning strategy.

**Specifications Table**

**Table:** 

Subject	*Social Sciences*
Specific subject area	*Education, Sociolinguistics* The learning of English in a country where English is dominantly spoken or where English is the official language. It also covers the study of language in relation to social factors, including differences of regional, class, and occupational dialect, gender differences, and bilingualism.
Type of data	i) Transcriptions – Classroom observation was chosen since the observational data collected was transcribed as a close representation of a natural classroom teaching and learning situation. Discourse was audio-recorded to confirm what had been spoken in the classrooms earlier and then transcribed. The intention was to record the code-switching in action and how it was used as a teaching and learning strategy in the classrooms. The samples of transcriptions were coded by two coders; one of them is the researcher herself and the data was then compared between these coders. The inter-rater reliability of the Kappa coefficient was 0.74, which was in the good agreement category. It was calculated basically as follows: the probability of two coders agreeing on the coding minus the probability of randomly agreeing on the coding divided by one minus the probability of random agreement [1] (ii) The Tables presented data derives from the classroom observations, interviews and questionnaires: Table 1 (functions of code-switching, descriptions and examples); Table 2 (demographic profile of the lecturers involved in the study); Table 3 (the number of student participants); Table 4 (The frequency of code-switching that was gathered from the classroom observation); Table 5 (duration of lecturer talk and percentage of lecturer talk); Table 6 (the functions of code-switching) and Table 7 (the parts of speech in the code-switching analysis); Tables 8 and 9 (lecturers and students preferred language in the classrooms); Table 10 and 11 list the reasons of the lecturers and students’ beliefs on code-switching English language usage. iii) Fig. 1 is a framework of code-switching analysis based on Macaro's [2] areas of teachers’ code-switching and the emerging functions from this research analysis. iv) Questionnaire - The questionnaire collected data to elicit factors that appeared to affect the speaker's choice of code and was appropriate for the students’ literacy level and asked about their use of L1 and L2 in the classrooms and daily life. The second section of the questionnaire, focused on participants’ language. The focus was on participants’ own views as the responses were required from the students. Apart from the Likert scale question type in the questionnaire, open-ended questions were also used to allow all the participants to share their views which will take lesser time to answer compared to an interview. The open-ended questions are used as a context of the participants [3] and they are used sparingly [4]. The data is analysed quantitatively. The research tools of the study, which were the observation sheet, interview questions and questionnaires were checked by the ethics committee of which had been granted approval (Ref: 011716). The validity of the questionnaire was tested using the Pearson Product Moment Correlations in the SPSS v.23. Based on the significant value obtained by the Sig. (2-tailed) of 0.000 <0.05, it can be concluded that all the items were valid. Based on the count value obtained, rxy 0.305 to 0.643 > r table product moment 0.149 (N=183), it can be concluded that the items were valid.
How the data were acquired	The data acquired from the questionnaires (analysed using SPSS), classroom observations (analysed using Nvivo) and the interview sessions with the lecturers after the classroom observations (also analysed using Nvivo) were to identify the code-switching used and their functions in the classroom.
Data format	Raw Analysed Filtered
Description of data collection	The lecturers who were selected for the sampling were varied in job-seniority (4 for junior, 2 for experienced and 3 for senior lecturers) and they represent the various age ranges of polytechnics lecturers. They were all English lecturers from the General Studies Department and would have similar knowledge of the subjects that they were teaching and familiarity with the department's systems. The other group of participants was the students. The students were recruited from the classes in which their lecturers were
	observed for the purpose of this research as they would be able to comment on their lecturers’ teaching styles as well as being able to inform their beliefs from a student's point of view. There was a total of 183 students from the three premier polytechnics in the Civil, Mechanical, Electrical or Commerce Departments who had agreed to participate. They were in their final English course (Communicative English 3, AE501) since they were the earlier batch of ETeMS (English in the Teaching of Mathematics and Science) previously from school and the first few batches of TLSMTE (Teaching and Learning of Science, Mathematics and Technical in English) in the polytechnics. The age range is between 19 and 21 years old.
Data source location	• Institution: Premier Polytechnics Malaysia • City/Town/Region: Perak, Selangor, Johor • Country: Malaysia
Data accessibility	Repository name: Mohamed Mokhtar, Mazlin (2021), “Code-switching in ESL classrooms of Malaysian Premier Polytechnics” Data identification number: Mendeley Data, V2 Direct URL to data: https://doi.org/10.17632/54hr8zjx8r.2 Questionnaire (the questionnaire is provided as a supplementary file)

## Value of the Data

• Why are these data useful or important?

These data are useful for researcher to refer to additional functions in the earlier framework which could enrich further research on code-switching (CS). Although using only the L2 could portray a real-life use of the English language where students are not expected to understand everything they hear, it would not be applicable in Malaysia as code-switching is used naturally especially during conversations. Since language keeps on evolving, English in Malaysia has also developed its new form, which is called Malaysian English (ME). It should be accepted that there are times more than one language can, and should, be used in an ESL lesson. Decisions about the choice of language used should depend on students’ backgrounds, proficiency levels, the objective of the lesson and the language function the lecturers are focussing on at the time. Code-switching should not be considered negatively but seen as contributing to more effectively L2 language acquisition if it practised properly and wisely.

• Who can benefit from these data, how can these groups or people benefit from it?

These data will benefit those who want to add their understandings of the theory and practice of CS specifically in the higher education context such as in schools and higher institutions, as well as to inform the policy maker especially on language policies in regard to the use of L1 in the English Language classroom for teaching and learning purposes. Anecdotally, the use of ‘English only’ in Malaysia throughout the years where teachers or lecturers have been warned by their superiors not to use the L1 at all in the L2 classroom, appears to have been practice based on an unwritten policy. Macaro [2] argues, however, that restricting the use of L1 does not support concept development. As students may already have the concept in their L1, using the L1 could help them understand new words or meanings in L2. L1 can be used to connect their thoughts and ideas with the new information they receive in L2. Increased use of L2 by either the students or teachers in the classroom may not imply students are using the language well. Therefore, code-switching could be a useful language skill to enhance the teaching and learning process and for students in acquiring the new language.

• How can these data be used or re-used for further insight, research, and development?

These data can also be used for further insights in confirming the functions identified and how the function of CS can be use as one of the teaching and learning strategies by teachers/lecturers.Further investigation is needed to establish whether the teachers’ beliefs about code-switching is similar to the students’ beliefs and to ascertain whether teachers have achieved the outcomes they have set to achieve in their lessons. Longitudinal research to identify change over time and impact on students’ achievements could be a great way to gather more data on code-switching. Larger samples that include other higher institutions could assess any diversity of practices as they may or may not have similar functions and communicative purposes used for code-switching. Group research involving a number of researchers across institutions would enable discussions of guidelines for, and the practicality of, code-switching in English Language classrooms, adding to knowledge of code-switching and better understandings of issue that arise.

## 1 Data Description

(i) Transcriptions are based on the recordings from classroom observations and interviews. Classroom observation was chosen since the observational data collected was transcribed as a close representation of a natural classroom teaching and learning situation. Interviews were also transcribed to identify the common themes of code-switching functions and beliefs.

ii) Table 1 and Table 4, Table 5, Table 6, Table 7 in the data repository was collected from classroom observations and interviews. Table 1 shows the functions of code-switching, descriptions and examples that were identified during the classroom observations. They are based on Macaro's [2] five areas of code-switching occurrence in the classroom: *building personal relationships with learners, controlling students’ behaviours, giving complex procedural instructions for carrying out an activity, teaching grammar explicitly*, and *translating and checking understanding*.

**Table 1:** Functions of code-switching, descriptions and examples.

Label	Description	Example
1. Building personal relationships with learners	Serves as a way to create closeness, solidarity and intimate relations with the students/lecturers	T: Designer, ok. Whatever that is not stated as the requirement, *bukan keperluan*, ***(not a requirement)*** but you feel that it will help you, it will give you more strength in application, extra knowledge *la*. Extra skills *la tu. Kan?* (.) *Betul? Macam tak faham je.****(Right?*** ***Like don't understand)*** Ss: *Faham****(Agree)*** T: *Kalau faham kena senyum, kalau tak faham kena kerutkan kening.****(Smile if understood, or frown if you don't)*** ((Laughing)) T: So, *kita kena fahamlah sebab senyum ye. Kan.****(we need to understand and that's why we smile. Right.)***
2. Giving complex procedural instructions for carrying out an activity	Serves to reinforce, emphasise or clarify messages that might not be understood	T: Ok, now, you need to put them into: six–six groups. Going up, going down. No change, going up and down, small changes, big changes and low points. Ok, what I want you–I give you about five minutes, what you need to do is you need to put those words accordingly. Ok? The words that you have, *letak tempat yang betul*. Ok? Now, if you do not know the meaning, it's ok. Guess. ***(put accordingly)***
3. Controlling students’ behaviours	Serves to get students’ attention and concentrate on the lesson	T: Discuss in English. Discuss in English. That has never happened. *Kalau* discuss *je, meletup bahasa Melayu*. ***(When doing discussion, Malay language will burst out)***
4. Translating and checking understanding	Serves to speed things up because of time pressures and to transfer the intended meaning in order to avoid misunderstanding	T: Supposed to be going down? ‘Reached a low’. What is the word ‘reached’ means? Reach me. Reach. Ss: *Sampai*. T: *Sampai*. ‘Reached a low’. *Sampai* a low. ***(until)*** Ss: Low point T: Yes, why? Because it reached: a: low: level. ‘Reached a low’. It's not that much, is there.
5. Teaching grammar explicitly	Serves to teach grammar using L1 only or L1 & L2 together	T: Going up, ‘increase’, ‘grew’, ‘line up’. Ok, another word ‘rose’. ‘Rose’ is the past tense of ‘rise’: R I S E. ‘Rose’ is the past tense ↑of: ‘rise’. *Bukan nasi yang kita makan tu.* Ok? ‘Rise’, S E not C E. They grow, almost same spelling different meaning. ***(Not the rice that we eat)***
6. Direct Malay words or acronyms	Serves as generally known fact by L1 words that are familiar to the students	T: I believe you have your Industrial Training, *L I, Latihan Industri*, right? Ok, so make sure you remember what you have done in your er: *L I*. ***(acronym for ‘Latihan Industri’ which means Industrial Training)***
7. Malay slang/English + Malay particles	Serves to lower the language barrier that might have between myself and the others	T: How do you know? I know *lah*! ***(yeah)*** Ss: The size–the size–the size of advertisement T: It's not because I know *lah*! ***(yeah)*** Ss: No T: You see people said, I know *lah* this one. Small one! What–what–how much they can write, right? Isn't it? ***(yeah)***
8.Compensating for lack of vocabulary	Serves as replacement of words/phrases to get the lessons going	T: And the *apa* handover–handover date will be that progression for this presentation. ***(what)***
9. Giving explanations	Serves to explain a message from one code to another code either literally or in somewhat modified form for clarity and comprehension.	T: Rapport is like you just you build relationship. You build a relationship a bomb, no need er: details. Besides, the introduction is to the interviewer. You *kenalkan diri* you. ***(introduce yourself)***
10. Giving simple instructions	Serves to get students’ attention and trust before proceeding to the teaching of concepts or theories, especially to those students who are weak	T: *Ya, semua* mention. ***(Yes, all)***
11. Accommodating students’ code- switching	Serves to compensate students’ fluency and to respect those who are not fluent in either languages	Ss: That is in B M, ‘stabilise’ is *stabil*. Stabilise is *stabil*. E I, *betullah, betullah*. ***(stable, that's right)***
12. Giving clues	Serves as a change in topic and to get students’ attention	T: Yes, you are in the formal condition, ok? Sometimes your: shoes come with variety colours of erm: Ss: *Tali****(rope)*** T: What is it called? Ss: *Macam*: ***(like)*** rope–rope T: Rope? *Tali kasut tu*? ***(that shoes lace)*** Ss: Shoes–shoes lace T: Ah: Shoes lace. Perhaps it comes: with lots of colours, right? Ss: Shoes lace *tali kasut****(shoes lace)***
13. Teaching vocabulary	Serves to explain the vocabularies that might be new or unfamiliar to the students	T: Right, now no change it means it does not go up or down. It's just stays the same. At one point it doesn't change. In Malay, can anyone tell me, a word that you–not same as the word–word that means does not change. It starts with the word ‘M’. ‘M’ *jugak pun ah:****(also)*** Ss: *Mendatar****(horizontal)*** T: *Mendatar*. Yes, *mendatar*. That means no change, right? ***(horizontal)*** Ok, good. Right, up and down, ‘zig zag’ it means it's not stable. Ok, it's not stable. And suddenly you gonna have a heart attack. Is your pulse stable? No, right? Goes up and down, up and down, up and down. So what if very loose? What happens to your pulse? Ss: *Mendatar*. ***(Horizontal)*** T: Ok, *mendatar*. ***(horizontal)*** What do you call it in English? No: change, right? You know that beep? It goes up and down, up and down, up and down, you're still alive but not stable, right? But not stable. So, if it stops? Ss: Teettt ((sounds of beep)) T: Teettt ((sounds of beep)). Are you still alive? Ss: No. T: No, but the line is? The same position, stays the same unless somebody revises you. Then it goes up and down, up and down. Ok? Same concept. Remember that. Ok?

**Table 2:** Demographic profile of the lecturers.

Lecturers	Academic Qualification	Age	Class	Gender	Poly	Race	Years of Teaching Experience
PUO Lecturer A	B. Ed TESL	36	DKP3B	Female	PUO	Malay	11
PUO Lecturer B	B. Ed TESL (Hons.)	51	DKB5	Female	PUO	Chinese	27
PUO Lecturer C	Degree	27	DUT5B	Female	PUO	Malay	3
PSA Lecturer A	Master TESL	41	DPB5C	Female	PSA	Malay	10
PSA Lecturer B	B.A. Linguistics	50	DPB5B	Male	PSA	Malay	25
PIS Lecturer A	B. Ed TESL	28	DJK5C	Female	PIS	Malay	4
PIS Lecturer B	B. Ed TESL	29	DFP5C	Female	PIS	Indian	5
PIS Lecturer C	B. Ed TESL (Hons.)	26	DEP5B	Male	PIS	Malay	2
PIS Lecturer D	Master TESL	57	DRI5B	Male	PIS	Malay	34

**Table 3:** Number of student participants.

	Frequency	Per cent
PUO	67	36.6
PSA	55	30.1
PIS	61	33.3
Total	183	100.0

**Table 4:** Frequency of code-switching.

	PUO			PSA		PIS				
Participants	A	B	C	A	*B	A	B	*C	*D	TOTAL
Lecturers	11	3	8	2	**21**	6	10	8	**32**	101
Students	2	5	4	0	1	9	5	3	1	30

*Note:* * = Male lecturers

**Table 5:** Duration of lecturer talk and percentage of lecturer talk in L1 and L2 from the nine lecturers.

Participants	Length of lesson (min/s)	Length of lecturer talk (min/s)	Total lecturer talking time (%)	Lecturer talk in L2 (min/s)	Lecturer talk in L2 (%)	Lecturer talk in L1 (min/s)	Lecturer talk in L1 (%)
PUO Lecturer A	48’46”	42’11”	86.1	41’32”	85.3	0’39”	0.8
PUO Lecturer B	51’23”	47’49”	92.1	47’44”	92.0	0’05”	0.1
PUO Lecturer C	53’23”	39’29”	72.6	38’45”	71.7	0’44”	0.9
PSA Lecturer A	45’01”	41’24”	93.2	41’31”	92.5	0’33”	0.7
*PSA Lecturer B	36’35”	28’19”	77.5	28’21”	76.4	0’38”	1.1
PIS Lecturer A	48’57”	41’14”	84.2	40’22”	83.1	0’52”	1.1
PIS Lecturer B	49’00”	40’13”	82.1	40’08”	81.2	0’45”	0.9
*PIS Lecturer C	29’05”	26’44”	91.2	26’05”	89.8	0’39”	1.4
*PIS Lecturer D	38’06”	36’34”	95.3	33’17”	85.9	3’57”	9.4

*Note:* * = Male lecturers

**Table 6:** Functions of code-switching analysis.

	PUO			PSA		PIS				
	A	B	C	A	*B	A	B	*C	*D	TOTAL
1. Building personal relationships with learners	2	1	2	0	5	0	2	3	7	**22**
2. Controlling pupils' behaviour	0	0	0	0	2	0	0	0	4	6
3. Giving complex procedural instructions for carrying out an activity	0	0	0	0	0	0	1	0	0	1
4. Teaching grammar explicitly	0	0	0	0	0	0	1	0	0	1
5. Translating and checking understanding	1	2	1	0	0	2	7	2	6	**21**
6. Direct Malay words or acronyms	0	0	4	0	1	3	1	1	1	11
7. Malay slang/English + Malay particles	8	0	0	2	8	0	0	0	6	**24**
8. Compensating for lack of vocabulary	0	0	0	0	1	0	0	0	0	1
9. Giving explanations	0	0	1	0	3	0	3	2	5	14
10. Giving simple instructions	0	0	0	0	3	0	0	0	6	9
11. Accommodating students' code- switching	1	5	4	0	1	8	5	2	0	**26**
12. Giving clues	1	0	0	0	0	2	0	0	3	6
13. Teaching vocabulary	0	1	1	0	0	1	9	1	3	16

*Note:* * = Male lecturers

**Table 7:** Parts of speech in the code-switching analysis.

Parts of speech/Linguistic features	Total	Percentage (%)
Noun	63	**17.3**
Pronoun	36	9.9
Verb	104	**28.5**
Conjunction	14	3.8
Adverb	36	9.9
Adjective	56	**15.3**
Preposition	7	1.9
Malay slang	28	7.7
Malay word	14	3.8
Acronym in Malay language	7	1.9
TOTAL	365	100

**Table 8:** Lecturers’ language choice in the classroom.

	Frequency	Percentage
English only	4	44.4
Both	5	55.6
Total	9	100.0

**Table 9:** Students’ preference on lecturers’ instructional language in the classroom.

Frequency	Percentage (%)	
English only	15	8.2
Malay only	2	1.1
Both	164	89.6
Total	181	98.9
Missing data	2	1.1
Total	183	100

The additions of functions that were identified throughout the analysis are *direct Malay words or acronyms, Malay slang/English + Malay particles, compensating for the lack of vocabulary, giving explanations, giving simple instructions, accommodating students’ code-switching, giving clues*, and *teaching vocabulary*. The descriptions for each function with examples are shown in Table 1.

Table 2 provides the demographic profile of the lecturers involved in the study and Table 3 shows the number of student participants. Participants were recruited from the three Malaysian Polytechnics located in the North, Central and South of Malaysia. The lecturers were from the Language Unit of the General Studies Department, teaching the final year students. They were both male and female, aged between 26-57 years old and with a range of seniority and teaching experience: three were from Polytechnic A, two were from Polytechnic B and four were from Polytechnic C. There were more males (n = 3) than females (n = 6). Seven lecturers with a B.Ed in TESL, one has a B.A in Linguistics and one did not record her qualification. As they were all English lecturers from General Studies Departments, they would have had similar knowledge of the teaching subjects and familiarity with their department's systems. Participants were selected using purposive sampling where any English Lecturer teaching final year students at the Malaysian Polytechnics was allowed to participate in this research. The second group of participants comprised the students in the classes taught by the participating lecturers who were observed for the purpose of this research. They were included in the research as they could comment on their lecturers’ teaching styles as well as being able to give their perception of the issues being investigated in the research. 134 students, aged 19 and 21 years old in their final English course (Communicative English 3, AE501) from the three Malaysian polytechnics in the Civil, Mechanical, Electrical or Commerce Departments agreed to participate. They had previously completed ETeMS (English in the Teaching of Mathematics and Science) in schools and TLSMTE (Teaching and Learning of Science, Mathematics and Technical in English) in their respective polytechnics.

The frequency of code-switching that was gathered from the classroom observation was then presented in Table 4. The two highest frequencies of code-switching functions, *Malay slang/English + Malay particles* and *accommodating students' code-switching*, were not listed in the Macaro [2] functions of code-switching. This may be because the context of this research differed from the context in which the taxonomy has been established. In this study, English was the L2 and most of the participants had a common L1, Malay language, although they came from different races and background. The highest frequency of code-switching functions used by students in the classroom was accommodating students’ code-switching when lecturers asked them to explain the meaning of certain words. The use of the particle *‘lah’* was apparent in the data adding to the high frequency of the function, Malay slang/English + Malay particles.

Table 5 is to show duration of lecturer talk and percentage of lecturer talk in L1 and L2 in order to triangulate this data with the frequency of code-switching. There was no evidence however that the quantity of teacher talk was related to the frequency of code switching. For an example, Poly B Lecturer A had 93.2% of lecturer talk but only 0.7% of code-switching; similarly Poly A Lecturer B with one of the highest lecturer talk among the other lecturers (92.1%) had only 0.1% of code-switching as shown in Table 5. Therefore, it is unlikely that a lecturer with a high frequency of lecturer talk would also code-switch frequently in the classroom when teaching the English Language subject in the Malaysian Polytechnics. Poly A Lecturer B and Poly B Lecturer A both had high percentages of lecturer talk but low percentages of code-switching. It is possible that these lectures were able to use explanations in English to ensure that students understood without having to use their L1, which is the Malay language.

The next table, which is Table 6 represents the functions of code-switching that were used during the lessons observed. A total of 158 episodes of code switching were observed in the classroom observations of the nine lecturers. The highest frequency of code-switching functions were accommodating students’ code-switching (26 times), Malay slang/English + Malay particles (24 times), building personal relationship with the learners (22 times) and translating and checking understanding (21 times). The least frequent functions were compensating for lack of vocabulary (1 time), giving clues and controlling pupils’ behaviour (6 times).

At the same time, the parts of speech in the code-switching analysis were also listed in Table 7. Parts of speech and linguistic features identified in the code-switching during the classroom observations from all the lecturers. The unit of analysis differs from that in the frequency and function analyses. Each code-switched word was considered as one part of speech each time it appeared. As can be seen in Table 7, code-switching occurred most frequently with the verbs (28.5%), nouns (17.3%) and adjectives (15.3%) of the sentence. Most of the words or phrases that were elected in this research were the verbs and the next one was the nouns. This was mostly due to the choice of the speakers’ intentions.

Both Tables 8 and 9 are to present lecturers and students preferred language in the classrooms. While lecturers had positive views, generally about code-switching and its role in the teaching and learning process, some appeared to have reservations because of its possible negative impact on the language learning process. In the questionnaire, lecturers from Poly B and Poly C said they preferred to use both languages. Poly B Lecturer A and Poly C Lecturer D both reported they were “comfortable and have no problem using both languages…fluent in both”. They said they did not feel awkward using both languages. Similarly, Poly C Lecturer C stated, “teaching English in both languages can help my students to learn because they have different proficiency in English,” while Poly B Lecturer B wrote in the questionnaire that “sometimes students’ level of English language competency is below par, so I need to explain in the native language so that they understand better”. On the other hand, the majority of the students, 89.6% (n = 183), preferred their teacher to use both languages in the classroom; only 8.2% of the students expressed a preference for only English language during the lessons. When asked about their choice for an instructional language in the classroom, most reasons given by the students for their preference for both English and Malay languages related to understanding lessons. Responses included “weak students could follow easily or understand better”; it would “avoid misunderstanding”; “both languages will be used for communication in the future”, and thus, by using both languages, students could “improve their skills and language”. Students’ choice of appropriate language, or languages, to be used during an English lesson appears to be related to the importance of understanding of the lessons for them to acquire the skills and language, and to improve their English language. Code-switching therefore seems to be valued as a teaching and learning strategy in the English Language classrooms. Whether it is an English only lesson, or a lesson with dual language, what matters to students is understanding the lesson and acquiring the L2.

Table 10 and 11 look at the reasons of the lecturers and students’ beliefs on code-switching as well as to the importance of using the English language in their daily lives. Overall it appears that the majority of the lecturers believe that code-switching has positive impacts on the language learning process. The students (n=183) responses to the statements were similar but the percentage of those who agreed was less. Their agreement with each of the statements that code-switching would be beneficial when used as one of the teaching and learning strategies ranged from 68.3% - 94%. Most of the students (94%, n=172) agreed that code-switching would be able to show “respects to others who are not fluent in either language”. This is one of the statements that students had similar belief with the lecturers. A lower percentage of students agreed with other statements in comparison with the lecturers. There also some negative beliefs about code-switching. An average of 92.6% of the lecturers did not believe that code-switching had negative implications in the classrooms. However, 82.5% (n=151) of the students’ agreed that code-switching was used to “cover up my weaknesses in English language”, most likely because of lower levels of competency. Since all the English language lecturers were expected to be competent in the language, they would be unlikely to agree that code-switching would be used for this purpose. However, two of the lecturers agreed with the statement. The disparity between the positive and negative beliefs category suggests that, although most lecturers believed that code-switching was a useful teaching and learning strategy, they were also aware of some potential negative effects. Table 11 shows the highest frequency of English usage was related to their studies such as the use of the Internet and word processors, followed by presentations in the classrooms, reading academic books as well as writing memos and reports. These items would be related to their studies where they need to surf the Internet to do research, read academic books for references, present their assignments in the classroom and after that, the students need to write reports on what they have found and presented. Most subjects in the polytechnics are taught in English so the process of preparing, presenting and writing are completed using English, hence the high frequency of English in those activities.

**Table 10:** Beliefs about code-switching.

(i) Positive beliefs of the effects of code switching on the language learning process				

	Lecturers		Students	
	Agree (%)	Disagree (%)	Agree (%)	Disagree (%)

Know both English and Malay languages very well.	100.0	0.0	90.2	9.8
To create closeness among my students/friends.	88.9	11.1	72.7	27.3
To lower the language barrier thatmight have between others and me.	100.0	0.0	73.6	26.4
To respect others who are not fluent in either languages.	100.0	0.0	94.0	6.0
To transfer the intended meaning in order to avoid misunderstanding.	100.0	0.0	89.1	10.9
To reinforce, emphasise or clarify messages that might not be understood.	100.0	0.0	88.0	12.0
To use both languages equally either at the Polytechnic or at home.	77.8	22.2	68.3	31.7
Average	95.2	4.8	82.3	17.7

(ii) Negative beliefs of the effects of code switching on the language learning process				

	Lecturers		Students	
	Agree (%)	Disagree (%)	Agree (%)	Disagree (%)

Just to show off that I know both English and Malay languages.	0.0	100.0	27.5	72.5
To show some western value/status in myself.	0.0	100.0	38.8	61.2
To cover up my weaknesses in English language.	22.2	77.8	82.5	17.5
Average	7.4	92.6	49.6	50.4

**Table 11:** Students’ usage of the English language in their daily lives.

	Frequent (%)	Not Frequent (%)
Listen to radio stations that use English.	66.7	33.3
Watch movies or shows shown on television.	94.5	5.5
Speak with your friends/family.	32.8	67.2
Use the Internet to either email or do homework/assignments.	92.9	7.1
Use word processor programmes such as Word, Excel to do homework/assignments.	92.9	7.1
Do presentations for classroom assignments.	86.3	13.7
Read magazines/story books during your free time.	44.8	55.2
Read books related to homework/assignments.	71.4	28.6
Write on forms of memoranda or reports, etc.	59.0	41.0
Order and buy food and drinks.	33.9	66.1
Average	67.5	32.5

iii) Fig. 1 in the data repository indicates the framework of code-switching analysis based on Macaro's [2] areas of teachers’ code-switching and the emerging functions from the data collection of the research. There were five functions of code-switching originally, however, additional eight other functions were identified from the research. Thus, code-switching could create more opportunity for communicative purposes. The framework presented demonstrates Macaro's [2] code-switching functions together with those that were identified in this research. The functions are related to the beliefs of the teachers and students have about code-switching. Positive and constructive perceptions of code-switching, held by lecturers and students, may benefit the teaching and learning process in the classrooms. iv) The questionnaires are provided as supplementary files. A questionnaire was chosen for the students to put their thoughts down individually. With a questionnaire there was a possibility, with a larger sample of students, that the results gathered would be more and could be linked to data from the observations and interviews .The questionnaire, therefore, used simple words with the opportunity to request clarification from the researcher. During the pilot testing, the questionnaire was tested and feedback was given by the respondents. Changes and editing were done later to ensure that the questionnaire is valid for the actual research. In addition,the questionnaire contributed to the triangulation of the research data where more information and resources with data generated from different methods.The data analysed from the questionnaires are presented in Table 2 (demographic profile of the lecturers involved in the study); Table 3 (the number of student participants); Tables 8 and 9 (lecturers and students preferred language in the classrooms); Table 10 and 11 (reasons of the lecturers and students’ beliefs on code-switching English language usage) as described in the sections above

**Fig. 1:**
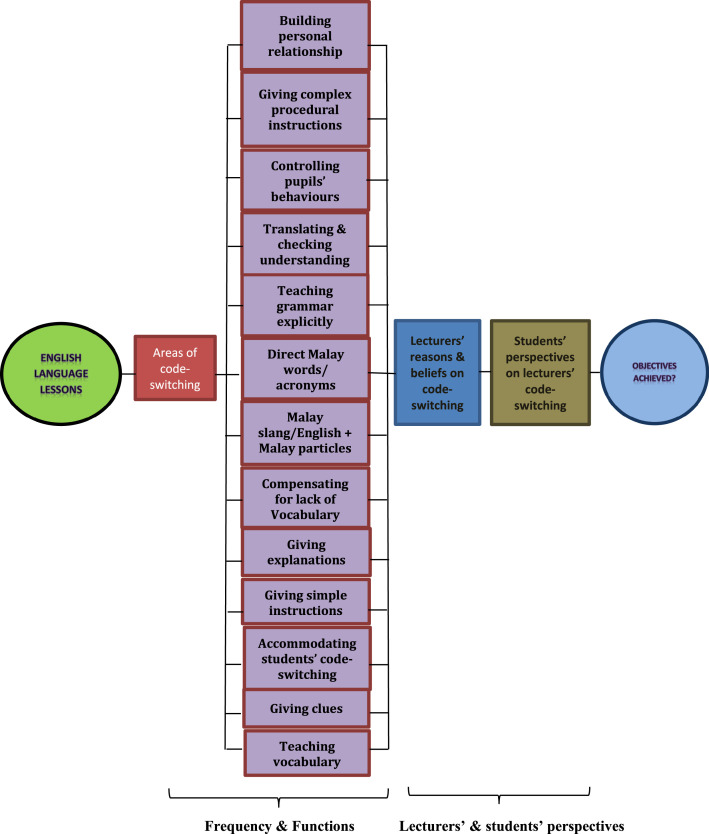
Framework of code-switching analysis based on Macaro's (2005) areas of teachers' code-switching and the emerging functions from this research analysis.

All the data can be retrieved at https://doi.org/10.17632/54hr8zjx8r.2 entitled “Codeswitching in ESL classrooms of Malaysian Premier Polytechnics.”

There are also two files of tabulated data provided as supplementary files: Tabulated data 1 – Students’ Survey, and Tabulated data 2 – Lecturers’ Survey

## 2 Experimental Design, Materials and Methods

The data was collected using a mixed method study of convergent parallel design, conducted using classroom observations, interviews and questionnaires to triangulate the data obtained from the three Premier Polytechnics in Malaysia, which involved nine lecturers and 183 students. A self-completion questionnaire was adapted from “ESL (ELL) Literacy Instruction a Guidebook to Theory and Practice” [5] which are related to the literacy level and the use of L1 and L2 in the classrooms and daily life. The second section of the questionnaire focused on the language use of the participants. It was adapted from a sample survey [5], which was supposed to be filled out by parents of children attending school. However, the focus of the survey was changed to the participants own views instead of focusing on other people's practices or views. For example, “What was the language of instruction in the home country?” was changed to “Which language do you speak/hear most at home?” as the responses were required from the students and direct questions will be easier for the students to understand. Apart from the Likert scale question type, open-ended questions were also used to allow all the participants to share their views which will take lesser time to answer compared to an interview. The open-ended questions are used as a context of the participants [3] and they are used sparingly [4]. The data is then analysed quantitatively. The validity of the questionnaire was tested using the Pearson Product Moment Correlations in the SPSS v.23. Based on the significant value obtained by the Sig. (2-tailed) of 0.000 <0.05, it can be concluded that all the items were valid. Based on the count value obtained, rxy 0.305 to 0.643 > r table product moment 0.149 (N=183), it can be concluded that the items were valid.

The data acquired from the questionnaires (analysed using SPSS), classroom observations (analysed using Nvivo) and the interview sessions with the lecturers after the classroom observations (also analysed using Nvivo) were to identify the code-switching used and their functions in the classroom. The convergent parallel analysis was used as both the qualitative and quantitative data were collected during the same stage of the data collection process. By having two different types of data, it can be used to confirm what had been identified and provided another view in the research as well [6]. This is because one method may not be efficient to stand alone without the other method to support it, where the results need to be examined and explained further to enhance its credibility. Both sets of qualitative and quantitative data were analysed and compared. They were then merged to present the results and analysis. This design type will help the researcher “to triangulate the methods by directly comparing and contrasting quantitative statistical results with qualitative findings for corroboration and validation purposes” (p.77) [6]. It was a parallel-database variant based on qualitative and quantitative methods [7]. With nine lecturers and 183 students’ participants involved in this research, it is hoped that the evidence will lead to a broad generalisation of the issue being studied in the Malaysian Polytechnics context.

A case study method with the main method of qualitative analysis was chosen with the focus on the explanatory type of case study. This type of case study was chosen in order to seek answers that may be able to explain the causal links [6], for example, between a new teaching strategy with the beliefs that the participants have in accomplishing the outcome of the lesson. According to Yin [8], a detailed study of the participants and its evidence will be based on professional applications. The case study design needs to have five components, which are: the “research question(s), its propositions, its unit(s) of analysis, a determination of how the data are linked to the propositions and criteria to interpret the findings” (p. 59) [9]. This method is beneficial in terms of testing the theoretical models in different samples and situations as well as to see how far the model is applicable in the real world. There is a quantitative aspect with the qualitative method in this design. It would also be more valid when the analysis is synthesised and compared between the qualitative data and statistical results [7]. It will also allow several ways in analyzing the cases either in pairs or according to themes and triangulate across the cases [10]. Of course, the result from this analysis is not intended to be generalised for the whole world as it is meant to test the theoretical model by Macaro [2], the areas of code-switching in using different samples and settings to see how far the model would be applicable. Below is an overview of the research design:

**Fig. 2:**
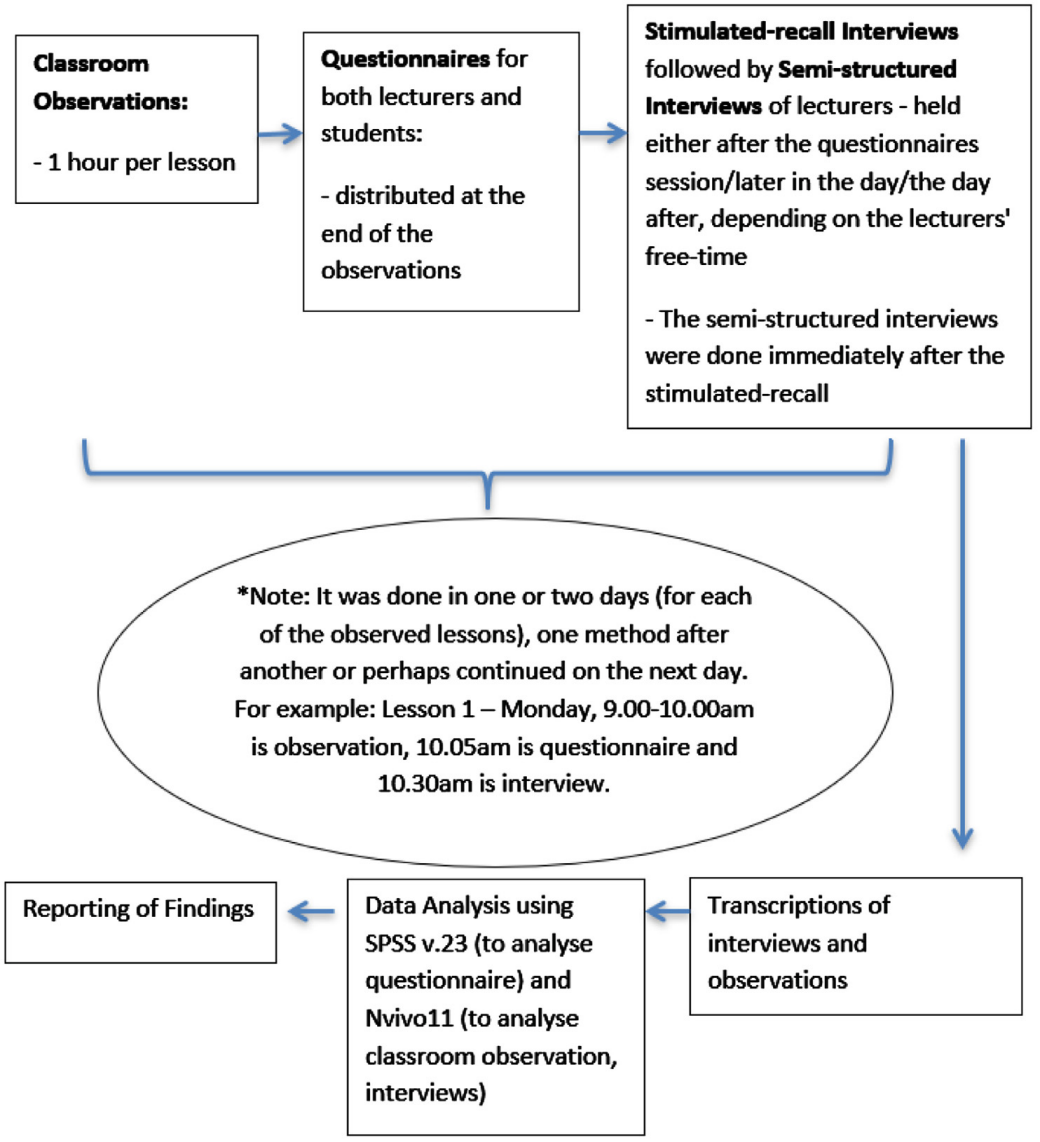
Overview of research design and the flow of data collection from each step.

## Ethics Statements

Before conducting a field study, an appropriate ethical arrangement was made by submitting an Ethics Application to the Ethics committee members of the University of Auckland, New Zealand. The research tools of the study, which were the observation sheet, interview questions and questionnaires were checked by the ethics committee of which had been granted approval (Ref: 011716). The validity was for three years. Applications were also sent to the Education Planning and Research Division (EPRD) of the Ministry of Education, Malaysia and the Economic Planning Unit (EPU) of the Prime Minister's Department to get their permissions to conduct this research at the three Malaysian Premier Polytechnics. A letter from the EPU indicating that permission was granted to conduct this research in Malaysia was received as well as a research pass given by the EPRD, which was valid for a year.

The next step was to contact the Director of the three Premier Polytechnics as well as meeting with the Head of Department or the Head of Language Unit to request for permissions in conducting this research at their premises. By having an early contact with them, it was easier for the department or unit to give access and arrange the timetable to accommodate to the research. The lecturers were chosen by their Head of Department or Head of Language Unit. They were briefed about the whole procedure of the research as the participants would be in doubt or had questions to ask, including other confidentiality issues were complied with.

## CRediT Author Statement

**Mazlin Mohamed Mokhtar:** Writing – original draft preparation; **Maizatulliza Muhamad:** Conceptualization, Methodology; **Aireen Aina Bahari:** Data analysis, Methodology; **Farah Natchiar Mohd. Khaja:** Reviewing and Editing.

## Declaration of Competing Interest

The authors declare that they have no known competing financial interests or personal relationships that could have appeared to influence the work reported in this paper.

## Data Availability

Code-switching in ESL classrooms of Malaysian Premier Polytechnics (Original data) (Mendeley Data). Code-switching in ESL classrooms of Malaysian Premier Polytechnics (Original data) (Mendeley Data).

## Appendix Supplementary materials

Image, application 1

Image, application 2

Image, application 3

Image, application 4

Image, application 5

Image, application 6
