# Supplementary material for: Dataset from Code-switching between English and Malay Languages in Malaysian Premier Polytechnics ESL Classrooms
Source: Data Brief. 2022 Oct 29;45:108709. doi: 10.1016/j.dib.2022.108709 (PMC9679697; doi:10.1016/j.dib.2022.108709)
Supplement: Supplementary file 1 [file mmc1.pdf]

i) PUO Lecturer A Transcriptions

Transcription of Classroom Observations

Recording No.: 2
Date: 3/9/2014 (Wednesday)
Time: 10.00-11.00am
Class: DKP 3B
No. of Ss: 20
Level: Mixed ability
Age range: 19-21 years old
Topic: Job Hunting Mechanics (Job Advertisement)

**Notes:*

T:	Lecturer
Ss:	Students
(.)	short pause
(0.2)	2sec pause
[	overlapping speech occurs
↑	rise in intonation
↓	drop in intonation
--	change of the topic/rephrase/repeat
::	elongated speech/ stretched sound
=	latched speech, a continuation of talk
( )	words spoken here were too unclear to transcribe
(( ))	transcriber's description
Ø	no talk, ambient noise
U N	capital & space for acronyms
red	Malay words
' '	Sentences read from the board/book/reading materials

**Based on Jefferson, G. (2004)*

T: The same topic has complete yesterday, right?
Ss: Yes
T: Ok. But er: still focus on a few things, alright? Ahh..as you know, we talked about job hunting mechanics. What is few techniques require?
(0.4)
((T wrote on the whiteboard))
T: Job hunting mechanics…ok? Right, first (.) what have you done before this?
S1: Ah: search for the
T: We have what? Analyse: the:?=
S1: =Job advertisement
T: Job: advertisement: is it right?
Ss: Yes
T: Ok. So, what did you look into a job advertisement?
Ss: Salary, company background
T: Ok. In the job advertisement, you will see company background--company's background, what else?

S1: Job requirement
T: Ah: you will also see ah: job requirement. Ok, what else? (0.2) Besides that?
S1: Salary
T: Ok, they also mention regarding salary. Besides ↑salary? Ok, they mention about the background: ((while T wrote on the board)) ok, what else? (0.2) Ah: just now
S1: Reference
T: Ah: just now requirement--job requirement
S1: Position applied
T: Erm:: salary
S1: Location, address
T: Ok
S1: Location
T: Ok, details--company's details, right. Location: er: email address, ah: what else? Contact number, alright? Alright, so this one is done. What else?
S1: Ways to apply
T: Ok
S1: Ways to apply
T: Ah: methods of application, so there are quite a few isn't it? Alright, so yesterday also, ok, we have--you have been asked to choose a: ↑company.
Ss: Yeah
T: Alright, which you and together with your partner find a suitable (.) based on ↑your: qualification, isn't it right?
Ss: Yes
T: Ok now, for that, alright, ah: I would ask you you to: give or come up with two reasons, ok? Have you identified the reasons why you choose the company?
Ss: Yes
T: Sure?
Ss: Yes
T: Very confirm?
Ss: Yes
T: Both of you have discussed?
S1: Not yet.
T: Not yet, ok. Shall we move from one group to another? We start with Group 10--we start with Group 10 first? Ah: group 10? Or should I give a few minutes to er:: sit together with your partner?
Ss: Yes
T: Ok now: find a partner. I told you yesterday, to already prepare the two reasons, ok? One, for each advertisement why you have already decide what the reason for you to choose for that specific company. (0.3) ↑Ah: (0.2) Because what?
((Ss sit with partner for discussion - 03:10 to 03:43)
T: Ok. Ah: are you ↑ready?
Ss: Yeah
T: Ah: (0.2) Come again? You don't want the advertisement? I told you yesterday to prepare. But yesterday? you have already finished, that's why you re-start? So you have to think of a reason. I already mentioned this yes: ter: ↑day. Alright, now. Erm: we are going to recall back one by one, ok, advertisement ok. Advertisement 1 would be from: DIC Malaysia, ok. And the job er: would be Lab Assistant Technician, Production Assistant and Warehouse Clerk. Did any group choose these company? Is there any group?--Is there any group choose DIC company?
S1: No

T: Ok, why?
S1: Because it is not related to our:
T: Ok, it's ↑not: related
Ss: Yes
T: Oh: You can't be Warehouse Clerk?
Ss: No
T: No: ok, alright. So this is not relevant. Ok, now look at Advertisement 2 that would be (05:00) searching for true business Explorer, from: erm: Finest & Admin Department of Sarawak Convention Bureau. Anybody choose this company?
Ss: No
T: No: why?
Ss: No, this company: not suitable
T: It's not suitable with you:
Ss: No
T: You are not a businessman?
Ss: Yes
T: Ok, it's my answer, how about you? What do you find it is not suitable?
Ss: Because it is not related
T: Again, it is not related with your study. Ok fine, ok. Look at Advertisement 3 that would be walk-in interview from shelving partner, which is Team Leader Despatch Animate Assistant and IT Technician. (.) Did you choose this?
Ss: No
T: Ok, all the group--all the pairs decided not to choose this job, ok? Now, we choose--we gonna move on to Advertisement 4, that would be Marine HVHC Engineer. (.) Which pairs choose this company? (.) Position would be er: yeah ok Marine HVHC Engineer, ok. Tan & Edward you choose this company. ↑Why? Could you stand up? One of you I need two reasons, right? Ok
S1: First it meets the requirement of our field.
T: Ok: second reason?
S1: Second because they pay--they have a holiday pay.
T: Yeah, that's holiday pay. Ah: that's interest you.
S1: Yes
T: You are so attracted to--to have that benefit. Ok, out of all, you choose this company?
S1: Yes
T: Ok, fine. Ok, Is there any other group who choose this ad? No? Ok, now we are gonna to move on to Advertisement 5. Jasa Marine (.) Seafarers.
Ss: Yes
T: Oh: I think one person from each pair--one person from each pair--one person (.) from each group. Ok, 1, 2, 3 so 3 er: pairs have chosen this job which is Seafarers.
Ss: 4!
T: 4? Eh again again again! Raise up your hands. Oh! 5, 6! Uh: 1, 2, 3, 4, 5, 6. Ok, now we shall listen. (.) Ok start now what's your reason? Seafarers?
S1: The position that we choose is Chief Engineer, 1st Class QC and Marine Coaster. So, the reason is because er: er: when we go to that kind of company--ship er: there will be less work and there'll be less leave and at the same time the salary will be very high
T: Ok, alright.
S1: Besides that er: Marine Coaster you can still be here and measure here. So whatever it is, you can still be with your family and come back later. And another:
T: Ok, two reasons only, ok.
Ss: (laughing)

T:Ok, if you tell us, let's hear from the others. Thank you Saras! That's the reasons why both of you choose this company. Ok, move on to Bob.
Ss: Bob!
T: Bob and Raimi
S2: Because the company provide er: position suitable for us.
T: Ok, why is it suitable?
S2: For Engineer
T: Ok
S2: Er: practical good for CEO and Deep COC er: 4th Class. We get er: apply for this position
T: Ok:
S2: Second is the sailing time for this company is not too long
T: ↑Ok, why do you think it is not too long?
S2: Erm: *paling pun* 2 months. *Dia tak ada* change
T: Ok, that is why you apply for this job. Ok, 3rd pair, is actually behind. Can see, right? What's the reason?
S3: We choose this: er: what company? Because
T: Seafarers
S3:Ah: Seafarers, because the salary they give is erm:
T: Salary, what else? Why are you interested with the job?
S3: Is: enough for: us with Diploma and this position
T: Oh: it's related with your qualifications and your skills. That's the reason? Erm: ok next?
S3: Because the Seafarer place is in Malaysia
T: Oh it's also in Malaysia. So, you don't have to be far away from you family.
S3: Yes
T: That's the reason. Ok, right, ↑next! Ah yes, the fourth group?
S4: Our reason are our sea time is enough just one year suitable to apply the bigger position.
T: Ok, so you're looking into that.
S4: Second, we don't have any: competence certificate
T: You don't have ↑any?=
S4: =Competence certificate
(10:00) T: Ok, seriously?
S4: So, whether it is a suitable job
T: Whether it is a suitable job for you?
S4: For the new seafarer
T: For…?
S4: For new seafarer.
T: For new seafarer. Ok, that's a reason. Ok, 1, 2, 3, 4, 5! So we come to the 5th group yeah. Why--why you choose this seafarers job?
S5: Because our supersenior, maybe go to
T: Our supersenior? Wow! Supersenior, ok, alright.
S5: Work for this company.
T: Most of them work, as the seafarers
S5: So, we will feel more comfortable, if we work with them
T: Hmm: I did not know that. Alright, so it means that when you have these people that you know, it is easier.
S5: Yes--yes

T: Ok, so you find er: it's much comfortable: ↑oh: this group also yeah. You would consider, alright, one, next reason?
S5: Next reason is: I do not longer time on board.
T: Ah: Ok: Oh! Mistake, only don't prefer to be on hot floor.
((Ss Laughing)) Miss=
T: =You miss your family so much, ok.
S1: *Apa nak buat…*
T: Ah? *Apa nak buat?*
((Ss Laughing))
T: ↑Eh! Last group, group no.6. Why you choose this company?
S6: First reason is: ah: the company provide the: suitable position for us.
T: Ok:
S6: Related to our certificate of competency
T: Alright
S6: For the second (.) reason, the company is related to: oil and gas company. So, they have strong base for eco--ah: economic basis.
T: Oh: it's a stabilised company yeah
S6: Oh yes
T: So you find the company can guarantee you a good career and a good ↑money!
S6: Yes
T: Ok yes, alright. Seafarers, anyone else? Ok, you have not. Ok make sure *ye* you remember this advertisement cause I would also.--I will you need each to make a copy of this cause it is a continuous stuff. Remember that I said, we're in Job Hunting Mechanics, we'll do the err: the study on job advertisement, continue with--afterwards we're gonna look into job enquiries and other job enquiries, writing resume, covering letter, finally it will be: ↑the: job interview. Ok! Now move on to: no.6. Who has chosen vacancy on Alam 19 Sendirian Berhad? Ok, now give a reason. The position in Technical Superintendent and Executive H S C. Ok so why? Give reasons.
((2 students stood up))
T: Oh: one by one. Ok, why?
S7: The salary that companied paid is affordable
T: Oh: ok, one is affordable
S7: The job eh: provide is actually related with our studies
T: Ok
S7: Because as a Superintendent, we only to observe the ship from the--from the land so anything happen to the ship, we are the person sent to--who make step to: ah: to repair or something=
T: =When something happen: only. Then go? Oh: so you love the job that on land rather than on board
S7: Yes
T: Ok, sit down. Ok, family first, is it? Ok right, ah:: 6 done. Advertisement no.7: which compa--group? 7? 7? Done? No. 8? ↑Oh 7 is not there!
((Ss laughing))
T: Ok guys: I need to wear spectacles already now.
Ss: Sorry
T: Surely most of you do not want to do this. Thank you. Now is Technician at Meru.
((Ss laughing))
T: ↑Tettt! Right?
((Ss laughing))

T: Technician, right? Technician: hmm:: ok, leave it aside. No. 9 Practical Mechanical Internship, is it relevant ↑class or ↓look into it first? Did you apply for this job? Internship Practical Mechanical from:: where is this? Ok, Pearl OGP Sdn Bhd. Ok and ok! Ah: no. 10, Orque Sdn. Bhd., ok, how many groups? We start 1, 2, (.) only 2. The rest has already presented, right? Ok, there are 2 groups have chosen this ah: company. Quite a number of positions yeah Safety Quality Manager, Technical Superintendent, Operation Superintendent, Purchasing IT, so on and so forth. Ok, we start with erm: Aniq, is it?
S8: Ah yes
T: Ok Aniq, give 2 reasons. You can choose yeap reason one by one
S8: Ah, one by one? The job?
T: No no no! You:--each one of you can present one reason. Ah:: ok? Ah ok, why you choose this company?
(15:00)S8: Why I choose this company because
T: =and position
S8: There are:
T: Why?
S8: Ah: (0.2) we choose the Safety Quality Officer
T: Come again?
S8: Safety Quality Officer
T: Ok, safety (.) why Safety Quality: Officer?
S8: Because the: first is the position is very related to our course
T: Hmm:
S8: because we have the Oracle based and certificate
T: Ok, it's like a skill is it?
S8: Yeah
T: Alright, two reasons. Second reason?
S9: Ah: the salary is=
T: The salary is what?
S9: The salary is better than working at other company
T: Oh you compared with other company, the salary is--is--is much more interesting yeah?
S9: Yes
T: Ok
S9: The second one is the work is less stress and=
T: ↑Oh! Less stress
S9: =and modest. Just walking around
T: Oh, just walking around
((Ss laughing))
T: Ok, I will look into this, just walking around is it? Ok. (.) A: that's the reason why you choose this company?
S9: Yes
T: Ah: because of these reasons. Ok, sit down. Alright, next? Another pair, right? Ok.
S10: Yes
T: Which is--who is your partner? Ah! Right.
(0.6)
S10: Because I choose this company because related to our: ah: studies--field of study
T: Ok, related to your field of study. Two?
S10: No.2 ah: although working with big company but their ship only sail near coastal and not foreign going. I don't want to live apart from my family.

T: Ok, alright, ↑Thank you! That's the 2 reasons. No. 1 professional wise, no.2 very personal yeah. Hm:, ok. So these are among the reasons why you choose the company, ok. I'm sure er: prev--yesterday when I asked both of you to come in front, choose the one which is suitable er: I'm sure there are quite a number of criteria that you looked into. Ok, first one would be about ↑the:? What would be your first priority? The first thing that you would look into?
Ss: Salary, position, position!
T: Position, salary, somebody says positions, some says salary will be no.1, ok. Some says the company that strong is no. 1, ok? So you look into quite a number of reasons, ok. By analysing the job advertisement, as what we have written there and by looking into all the data or information given in the job ads. So, you have decided together with your partner, which advertisement or which job position is suitable with your qualification. Is it only based on qualification? What else you need to: erm: ↑well we are talking about this job, right? The new job that you're going to work so, you also look into this personal matter like not being far from parents, isn't it? Parents: or family?
Ss: Family.
T: Oh family: (.) alright so from your family: the distance also you look into, ok, ah: so on and so forth. There are--so far I've not listened any of you saying that because it is your interest. Does any of the job just now look into the job scope? Saras? Because it's your interest? The job that you are applying?
S1: Yes
T: It--it--it (0.2) So, it suits to your: interest?
S1: Yeah
T: Why do you say interest?
S1: Actually interest it doesn't necessarily come with interest actually, it comes with passion
T: ↑Ah: passion, yes.
S1: Passion to become a Chief Engineer. Chief Engineer is like one of the main qualified job on-board of the ship. The rest of the--to repair of to--so it's a go ahead to
T: Erm: that is mainly your passion when you take up=
S1: The work is
T: =this engineering thing, this is the passion. This the job that: ideally for you.
S1: Yes
T: Ah: ok, thank you. Alright, so one person is. Ok, we continue this aspect and these are the things ah: I would say that you have to decide together with your partner. Ok, we are gonna move on, right? So, we have look into in analysing job advertisement. What would be the second stage after you have analysed and decided? Could you tell me? (0.3) Ok, you have chosen: the company so, what is your next step?
Ss: Resume, application, preparation, contact--contact the=
T: You must make ↑job:: you must know--or must (0.2) job en--qui--ries ((while writing on the board)).
Ss: =Enquiries
T: Ok, so we are moving into job enquires. Ok, what is enquiry by the way?
S1: Question
(20:00)T: The word enquiries. What can you understand the word enquiries? Or enquire?
S1: To get to know more
T: Ha:: ok Tan says to get to know more about something. Ok, to ask ↑for: infor--mation
Ss: =Information

T: Ok, example like this right? Ha: do you think that they given you sufficient information?
Ss: No!
T: How do you know? I know *lah*!
Ss: The size--the size--the size of advertisement
T: It's not because I know *lah*!
Ss: No
T: You see people said, I know *lah* this one. Small one! What--what--how much they can write, right? Isn't it?
Ss: Yes
T: So, no. One because of the size. You--you'll definitely know--you can't put everything, ok. Compared to this advertisement, ok, they can write quite a number of things. Ok, ah: ok why--what making they will lead up?
Ss: The background?
T: What do you think when you compare this kind--this kind of advertisement, what do you they will: lead up?
Ss: No: company background
T: Details about what? Yes?
Ss: No company background
T: ↑Ha: they will might be, for this kind of advertisement, there will be no company's background. Besides that?
Ss: Address, erm: no pictures--job offer
T: Ok, so for that, let us take a look at the book, ok? Now, we are going into the job enquiries and all of you must know or be able to make an enquiry when you talk about job, alright? So, ahh::: turn to page (0.2) ah: 51. Moving to page 51, is it the same page? Ok, job enquiries. Ok, now job enquiries you must have the--you must know or you must be able to understand what is the reason actually that you have er: that you must er: acquire what's the reason that you should know or you should learn to make job enquiries. Ok, as I asked all f you just now, what would be your next step? Your second step would be--would be on making job: enquiries. Ok, what do you think enquires? I already asked you just now what do you understand by the word enquiries: to ask for information, ok? We are not er: asking for example. In the context of er: you and your new friend, ok? We are in the context of looking into the job ad--ver--tise--ment, ok? What would be the other information that you would like want to know from the company. Ok. So if you--if you take a look at the introduction, ok? So this is er: it states er: some of the reasons why you need to make a job enquiry, ok? To project a good impression, you need to acquire appropriate telephone skills ok you need to speak clearly and politely to ↑be: understood. Ok? Now when you are on the phone, what is most important?
Ss: Greetings
T: Erm: in terms of: (.) language. What is the most important area? Hallo! am I speaking to a ghost?
Ss: Pronunciation--pronunciation
T: Erm: er: er: ok! So, sometimes when we speak with the person--ok in the other line, you can't see the face right (   ) ok? You--you're on phone so the person depending on ↑your:=
Ss: =[Pronunciation
T: =[Pronunciation. That's why you have to speak clearly so that people ↑can: understand. This is the composition maybe that you focus on telephone. Telephone skills is ↑very important. Ok, so therefore language focus is looking into ah: quite a

number of things and when see this is an enquiry ok, *bila kita faham itu* enquiry? Enquiry is to ask. Ok so, I repeat. Enquiry itu *kita kena bertanya* ok? So I need you to understand that very well. *Bila bertanya*, what would be the language focus?
Ss: Erm:
T: Besides pronunciation just now? What would you ask? (0.2) Questions right?
Ss: Yes
T: Ah: so when you ask question, what type of questions you will be using?
Ss: Line--line
T: Ok. Turn to page 52. (0.5) Ok? Job enquiry is--is about asking questions ok? When you (25:00) talked about questions, automatically you'll know--you have to use this language. So they are quite a number of question types. Ok, we have the first one, what do you called it?
Ss: 'Wh-'
T: ↑Ha: 'wh-question', ok? So, have you used 'wh-qestions' before?
Ss: Yes
T: Yes, very often yeah, different kind of settings, different sc--scenario just now when you go to the canteen, you will ask your friend might be, what would you ask your friend? Your friend wants to go to the canteen just now?
Ss: 'What do you want to buy?'
T: Ah: 'what do you want to buy?'ok. Ah:: what else? By using 'wh-question'?
Ss: 'Which one would you like?'
T: Oh: 'which one would you like' oh: at the canteen *ye*! 'Which one would you like, karipap or er: sausage?'
Ss: 'How was the food?'
T: Ah: who is with you? Ah:: you are right! Ok, ah:: 'what you want to eat?' maybe. Maybe you want to buy for your girlfriends. This is what you said 'what do you want to eat?' So you go and buy something for your friends, ok? So that would be 'wh-questions'. You know 'wh-questions' ok, ah: examples given on page 52 when looking--when we are looking into the job advertisements, ok? We have 'wh-questions' for example asking who's on the other line ok, 'who is on the line, please?' 'Who', ok? Next, you have 'what is the main requirement of this job?' You use the word 'what', ok? 'Wh' right? 'When can I start working?' 'When' ok? Next, 'which job is suitable for my qualification?', 'how many days in a week do I work?' Ok? So these are among the 'wh-questions' that you will be using. (.) Alright, erm: there are quite a number also written, 'who' refers to people. When you use 'who' you must know when to use 'who', ok? So 'who' is referring to: people, ok? Next we use 'which' ↑for? When do you use 'which'?
Ss: Job
T: Class?
Ss: For this job
T: 'Which' is: used when you: ask ↑for?=
Ss: =Job position--job position--choice
T: Choice! When you want to make a choice, 'which job would you like to apply' for example. There are A and there will be B so you use the word: 'which'. When there is an option, you use the word 'which'. Ok? Next one, 'when'. 'When' talks about what?
Ss: Time
T: Ha: ok. 'When' you will use when you want to ask regarding the time. Ok, next one? 'What' it is for specific=
Ss: =Information
T: Information, things, animals or incidents in world occurrences. And finally 'how'?

Ss: Ways of incidents
T: Hm: is a question that you are ask on the way erm:: the situation happens, ok? So, notes--look at the notes. Answers to these types of questions are unlimited but they must relate to the information sort by ↑the?=
Ss: =‘Wh-questions’
T: ‘Wh-questions’, ok? Next one, ‘yes/no question’, ok? There’ll be ‘yes/no questions’, what would be ‘yes/no questions’, you ask for example like ‘do I have to work overtime?’--‘Do I have to work overtime?’ so the answer would ↑be?
Ss: Yes
T: Yes ↑or?
Ss: No
T: Only you know what, alright? So, it’s not like you: ↑yeah, you say ‘yes, you have to work overtime, daa--daa--daa:’, alright? So, ‘do I’, it doesn’t start with ‘wh’, ok? Is field--is work field found at the job requirements, then ‘yes/no’ again and ‘do I have to submit my resume?’ again you are looking at the two--only one answer there’ll be ‘yes/no’. Answers to these questions are limited to a simple ‘yes/no’, ok. So, first is the ‘wh-questions’ and no. 2 is ↑the?=
Ss: =‘Yes/No’
T: ‘Yes/No questions’, ok. And the last type would be ‘tag questions’, ok? ‘The job is on a permanent basis, isn’t it?’ Ok? You give the statement, then you confirm. You--you are asking them to confirm whether your statement is right or wrong, ‘isn’t it’ at the end, ok? It’s a ‘tag question’ and we have No. 2 ‘I have to know how to use computer, don’t I?’ alright: And the next one would be ‘computer skills is an advantage, isn’t it?’ And lastly, ‘I do have to go out for field work, do I?’ (0.2) Alright? So, these are what you called as erm:
Ss: [‘Tag questions’
T: [‘Tag questions’. Ok? Such questions are intended to ask ↑for confirmation, just to confirm like ‘are you single?’
(30:00) Ss: No, maybe, yes… ‘are you single?’ Ah::
((T jokingly coughing))
T: Ah: not available
Ss: Hahaha
T: Alright, so these are the thing that you have to look into, ok? Look at Task 1, alright, very quickly we’re going to do an activity of the words but let’s take a look at one here, Task 1, alright. So, this is an advertisement given, right?
Ss: Yeah
T: Ah: there’s an advertisement given which is er: on the post of Assistant Engineer and it is based er: in Subang Jaya, ok. The company is based in Subang Jaya, ok. While looking at the advertisement, do you find that the advertisement actually give you all the information?
Ss: Yeah
T: Does that advertisement ah: give all or have written everything that you want to find out about the company?
Ss: No
T: For sure it is not. Ok, so: if you look at the advertisement, it just states there: the externship--experiential programme, they want--they want to have gardening articles, job, er: few requirements and you are required to--interested, you are required ↑to: call them, ok? And below that ok, there will be a script given. Isn’t it right?
T: You have the script?
Ss: Yes

T: Ok. Now, if you take a look at it together, ok. Leave it for a while, ok. Ah: between these conversation, ‘A’ and Chris. Silent reading all, by referring to the advertisement and read the dialogue silently, right. Between A and Chris. If you have questions, ask questions, ok?
((Ss did silent reading - 31:45 to 31:59))
T: Short dialogue *ye*
((Ss continue silent reading - 32:02 to 32:12))
T: Who do you think A is?
Ss: Receptionist, HR Officer
T: Receptionist? Does ‘A’ a Receptionist? Do you think ‘A’ is the Receptionist?
Ss: No, Human Resources
T: Most probably you can see the A is the Human Resource Officer, ok? What else? Get ready to perform.
((Ss continue silent reading - 32:35 to 33:24))
T: Ok, done? So this is between whom and who? When you talk about this job erm: situation. Job hunting situation, A would consider as the HR Officer, how about B?
Ss: Jobseeker
T: Will be the jobseeker, ok. So these are conversations between two different persons which is on the line and then they are asking about er: the jobseeker ah: has asked quite a number of questions, ok. Could you analyse--first of all what would be the first question that the jobseeker asked: to the company or to the:
Ss: Qualification
T: HR Officer?
Ss: Qualification
T: Ok. What’s the question that he’s asked? Used, sorry. What’s the question he’s used?
Ss: Er: ‘yes/no Questions’
T: Ok. Can I have the questions? Please, questions?
Ss: ‘Can you list some of the responsibilities of an Assistant Engineer?’
T: Which one? ‘Can you--one by one?’ Ok, alright. So, if Human Resource actually asked ‘may I help you?’, isn’t it right? And please answer ‘I’m Christopher.’ Ok, then what did he do?
Ss: ‘I’m calling:’
T: ↑Ah: ‘I’m calling regarding the advertisement which appear in ↑The: Star.’ What did he do? (0.2) He? Yes Edward?
Edward: Purpose of calling
(35:00) T: Yes, he err: mentioned the purpose of him calling the company. State the purpose why you call the company. Alright, number--next, do mean adver--advertisement for the post that’ll be from HR, yes it is yes exactly. They said, ‘very well sir, may I know your qualification please?’ And Chris answer ‘I have a Diploma from Civil Engineering from PUO. Am I suitable for the post?’ What is he doing?
Ss: Check the positions
T: Ah: he is using ‘yes/no questions’, ‘Am I: suitable for the post?’ So he’s looking for? Confir=
Ss: [=mation
T: [=mation. Confirmation whether it suits--it suits with the job or not. So what’s the answer?
Ss: ‘Yes, you are.’
T: ‘Yes, you are.’ Ok, so this, a very simple isn’t it? Now, anything would like to ask any more of the questions that he’s asked?
(0.3)

Ss: Work experience
T: Ah: you also you ask regarding work: experience? Why? In the advertisement, it doesn't--does it state work experience?
Ss: No
T: It doesn't state err: regarding work experience. In the requirement, the company doesn't state that they want (.) a person with a work experience. So actually, do they want a work experience staff or not?
Ss: No. It is just an advantage.
T: Yeah. It states that (0.2) if you have work experience, it will be an added advantage, isn't it? Alright, next one what other question that he asked?
Ss: Responsibility
T: He also asked question regarding ↑the: responsibility. The job's responsibility. What is the job responsibilities?
Ss: Production
T: Ah: so HR mentioned he's already responsible for the production at the factory. Ok, next question? Third question, what did he ask?
Ss: Working hour
T: Ok, because why? In the advertisement, it is not stated what would be the working hour. Alright, so it is being used 'What are', 'wh' *ya*, what are the working hours like?' Ok next? What else did he ask?
Ss: Salary
T: *Ya*:: this is your favourite question, right?
Ss: Yes
T: Ah: ok. But, did he ask at the beginning? No, he like going down, ok, working as what then what did he--what he need to do. He moving down and he looking at things erm: digesting the information and then move into the salary part. So, er: what's the questions that he used?
Ss: Hm:
T: About the salary?
Ss: 'May I know what'
T: 'May I know what the salary is like?' ok. 'May: I: know:' could you start like 'What--what is the--what is the salary like?' Could you say like that?
Ss: No
T: Why cannot?
Ss: Not polite
T: Oh: it's not polite. So 'may I know' is polite.
Ss: Yes. It's better
T: It's better.
Ss: Yes
T: It's soothing. 'May I know what the salary is like?' Ok, it is more polite. Ok, polite, thank you. Ok, next one what's the last thing he asked? So, one thousand eight to: what's the salary? One thousand to one thousand five (.) hundred. So, do you think that he's interested?
Ss: No--yes!
T: He seems like he's interested *ya*. With the job scope, with the: working--working experience, with the: salary, with the time--working time. He seems like he's interested with the job. So what did he do?
Ss: Interview. He ask for interview.
T: ↑Ha!: he asked for an interview. He wants to arrange for interview appointment. So, he said that 'I like--I'd like to make an interview appointment'. Then he said, 'may I

know if it is possible for you to arrange that?' So, he's interested. Ok, finally what did he er: asked when the interview is being arranged? For the interview, what did he ask?
Ss: Reference. Company address.
T: Ok, so, erm: the HR Officer needs his full name and the company oh sorry contact number. So then (.) what else--what else did he ask?
Ss: Company address
((Ss sneezed))
T: He's already asked for the company address
Ss: 'Do you think you can come tomorrow?'
T: So finally he confirmed on coming to the interview. Alright, so close by seeing them 'I'll be there for the interview tomorrow.' 'see you then' and…
Ss: 'Bye'
(40:00) T: 'Bye':: ok, is it er: very simple conversation?
Ss: Yes
T: Ah: but very meaningful, right?
Ss: Yes
T: Why did you say so?
Ss: Because
T: Because?
Ss: More information
T: You make job enquiries because you want to--you had previously--you looked into a very comprehensive erm: job ad that you can get all the information. But sometimes, the job advertisement could also attract you to call them to find out more and you'll be to? (0.3) Decide, if they have given sufficient information, so straight away you can decide to go for ↑an:=
Ss: [Interview
T: [Interview. Ok, so can you done this before might be?
Ss: Yes
T: Oh yes. You have an experience before?
Ss: Yes
T: Ok when?
Ss: LI
T: Oh ok. Is it now?
Ss: Yeah
T: Yeah at the moment, right? Oh: ok, you've an experience. All of you have--already have a place for LI?
Ss: Yeap, yes, no
T: Oh? So you actually have to make: a call to the specific company and asking whether they can (0.2) take you, would they accept you?
Ss: Yes.
T: Yes, they really accept?
Ss: No! Not really
T: Not really. Maybe because you are not using the correct 'wh-questions', maybe. Right?
((Ss laughing))
T: Right? Maybe you did not use the: correct questions to ask them. Ok, so when we are talking about job enquiries, this is sample. This is a sample that you need to err: first alright, let's go back to this. In job enquiries, make sure you use the correct 'wh-questions', ok. Or you can also use…
Ss: 'Yes/No questions'

T: ‘Yes/No questions’ and the other one?
Ss: Tag
T: Tag questions, ok. Ah: this is very important. So this is when you would be able to get the correct information. Ok, if you ask the wrong questions, then you will not be able to get all the…information that you want. Ok, so in making job enquiries, you’ll have a purpose, isn’t it right?
Ss: Yes
T: So, you have already erm: you have already decided what would be the questions that you will look--you will ask ↑the other party. What would be the question the first thing? (0.2) What are among the questions? No. 1? You ask the company about ↑the?=
Ss: =Job application--advertisement
T: Erm: about the?
Ss: Job advertisement
T: ↑Job?=
Ss: =Advertisement
T: You ask the HR Officer about the? Job responsibility. Isn’t it right?
Ss: Yes
T: Ok, next?
Ss: Qualification
T: Ah: what is it you ask them regarding your qualification? You want to again to make sure that your qualification suits with the:
Ss: qualification
T: Er: need of the job. Ok, next? What other questions just now we have looked into? What else?
Ss: Working experience
T: *Apa lagi nak tanya soalan apa ni? Ada banyak soalan…soalan apa?*
Ss: Working experience, working hours
T: *Ya!* You would need to find out from their working hours. Do you understand? *Tanya tak cuti berapa hari?*
Ss: *Tak--tak*
T: When do you ask this question?
Ss: Er: interview
T: During ↑the:=
Ss: Interview
T: Interview. Ok, don’t add forgot forgot, keep your questions first, right? Look into this important matter. Ok, first you asked them the job responsibility er: then you go into qualification, right? After that, you er: you want to find out about their working hours. It’s very important, is it? Why? You cannot work 24-7?
Ss: Yes, we cannot.
T: You cannot
Ss: We are not robot.
T: You are not robot. You also have your own life.
Ss: Yeah
T: But you want to make lots of money!
Ss Yes, money is money, life is life!
((Students laughing))
T: Wah: ok Bob, understand.
((Ss continue laughing))
T: Ok next one! Anyone else, what else? Hmm: responsibility, qualifications, working hours, what else?

S: Err: hmm: salary
T: Oh: salary! Ahh: salary *ya!* Ok, sa: la: ry: Ok. Is there any other question?
Ss: No
T: These are among the main four questions that you will ask. And finally if you are already interested, what would you ask from them?
Ss: Interview
T: Ah: you gonna fix for ↑an?=
Ss: =Appointment
T: Interview. You arrange=
Ss: =Appointment
(45:00) T: =For an: (.) interview, ok? So these are among the:: enquiries that you will be making when you talk about these Job Hunting Mechanics, is it right? By using the 'wh-questions', these are the enquiries components that you will be using. Ok, so therefore what are we going to do next? What do you think?
Ss: Hmm::
T: Ermm:: ok, do you have your advertisement?
Ss: Yes
T: Yes or no?
Ss: Yes
T: Yes or no?
Ss: Yes--no
T: Ok, I'm gonna give back your advertisement (0.2) as we already learnt, you have one sample which is in the book, ok? There are two students in each err: group, is it right?
Ss: Yes
T: This is a special one, ok. So, one student will be as ↑a?=
Ss: =Interviewer, HR
T: HR Officer ok, and another student will be ↑the?=
Ss: =Job seeker
T: Job seeker, ok. So I want--by referring to the advertisement that you would have already identified, I want you to come up with a dialogue job enquiries based on the advertisements. Ok? Is it possible?
Ss: Yes
T: Sure. If you feel sleepy what you need to do?
Ss: Wash the face
T: Wash the toilet, is it ok?
Ss: No!
T: Wash your face not wash the toilet.
Ss: Yes
T: So make sure you try to complete this, I give you for about 15 minutes, ok? Discuss together with your partner and afterwards you have to present.
Ss: Oh:
T: Oh, yes, right. In front, thank you, ok. Make sure you use the appropriate 'wh-questions' err: you can choose either one or you can combine all, ok, in your dialogue. Is it possible? Yes! Ok:: I will change--ok now let me see the group. You are group 1, group 2?
Ss: Group 4
T: Group 4, ok. You're group 1? Ok, (0.5) group? Group 2?
Ss: 3
T: Group 3. (0.3) Er: Group 1? Come again?
Ss: 9

T: 9. Group? Ah: together sit next to your partner, goodbye!
((Ss moving place))
T: Ok, next? Group 4. (0.4) Ok, next? Group: (0.3) 6. Next group? You are group no. 10
((Ss still moving place))
T: And:: (0.3) so, I give you 15 minutes discuss together with your partner: and afterwards you are required to present in front of the class. You may refer to the sample that we have in the book but you are requested to er:: according to the job advertisement which has given to you, the authentic material, so you have to come up with this suitable: conversation on job enquiries, Ok? (48:46)

Transcription of Interviews

Recording No.: 3
Notes:
A: Interviewer
B: Interviewee

A: Ok, asslamualaikum and good morning.
B: Waalaikumussalam
A: (laughing) How are you today?
B: Ah: good, thank you.
A: Ok, thank you for agreeing to talk to me and the purpose of my research as you know is to investigate the use of code-switching amongst the English Language lecturers in the Malaysian polytechnics. Alright, I would like to have some views on lecturers practices and the use of code-switching in the classroom. So the content of our talk will be confidential and only be used for my research. Alright?
B: Aha:
A: Ok, so have you heard of code-switching?
B: Yeah I heard of code-switching which is commonly used in the classroom er: especially in Malaysia. You know that code-switch is very common, even though we have good students in the class, nevertheless we will come to a certain point or certain time, we will have to: code-switch our language which is for me ahh…Bahasa Melayu for them to understand better the--the topic or the discussion that being made so that they are--they can understand and the: objective of the lesson being achieved.
A: Hmm: so you did notice that you used some code-switching in your lesson?
B: Yes, that’s for sure.
A: Ok, so the reason is actually just want to make the student understand better of the topic?
B: Yeah--yeah because we have mixed ability students. When we have this kind of group, so we cannot like expect all the student to understand. They’ll be those who are good which can understand us throughout the lesson by using 100% English but they’ll be some who are not good. So therefore, we also have to look at this matter by code-switch that, for example certain terms, certain words. Ah…when we stressed thing, there are students who will not be able to catch. So therefore, by code-switch language, so erm: yeah mostly the classroom comprises of er: Chinese, Malay and Indian, and they’ll be able to understand example Bahasa Malaysia--Bahasa Melayu.
A: So it is common that everybody would know Bahasa Melayu, right.

B: Ah yes--yeah Bahasa Melayu. So code-switch is easy for them to understand.
A: So it is not a planned thing or it's unplanned?
B: It's unplanned. Usually when a lecturer or teacher code-switch, it will make--it will happen unplanned. It's not something that you plan.
A: Alright. Ok, what if we don't code-switching? Is there any other alternative that we can: use?
B: Yeah, there will be a time where we still err: our concentration is in make sure in making sure the students will be exposed to er: English throughout the lesson. But nevertheless, er: if we don't code switch, if I don't intend to code-switch, I will try like to explain by using--by demonstrating or using simple English words so, for them to understand words that they are familiar with or I'll to demonstrate the action so that they can understand. So this is--this is other approach that I will use if they'll not be, other than code-switch.
A: Ok.
B: Is still they did not understand, the process or the idea or the topic, then the final stage would be code-switch.
A: I see. Alright, let's move on to another part of the interview which is more of the teaching of English as a general not the lesson before.
B: Ok.
A: So, your first language would be Bahasa Melayu, right?
B: Yeah, Bahasa Melayu.
A: So English is the second language.
Yes.
A: Any other language apart from that?
B: No--no.
A: Ok, what subjects are you teaching?
B: Ah: at the moment I'm teaching 3 main subjects, that will be AE: Communicative English 1, Communicative English 2 and Communicative English 3.
A: Alright, so 3 subjects. So how long have you been teaching?
B: Erm: almost 11 years.
A: Wow! So do you have any particular interest on certain: subjects? Which one would you like the most?
B: Erm: I would consider that the most err…the particular subject which I like the most I would say challenging and really test my ability would be Semester 1, A E Communicative English 1.
A: I see. Alright, do you teach in English all the time or?
B: Are you saying that throughout the times mean throughout the lesson I'm using English 100%?
A: Yeap, yeap all the times, not only in lesson yesterday, the other lesson as well, the other classes. Is there any difference? I mean, do you speak more English in certain classes or less English on other classes or is it quite the same?
B: Ah: quite the same.
A: I see. They are all of mixed ability
B: Yeah, they are mixed ability students.
A: Ok, so if you don't use English 100% in the class, do you feel guilty about it or you just or you don't feel guilty at all?
B: Don't feel guilty at all. Ok, er: seriously, when we become a teacher, our er: our focus our aim is to let the students learn. So if we are--we fail to make them learn, I think I will feel much guilty on that. So therefore, I...I feel that the process of making them to understand and learn, there are quite a number of things that we have to consider,

alright. So therefore, ahen I'm using the code-switch approach, don't say that I'll feel totally guilty of I have done, because I wanted them especially those we called as a weak--weak students, we also need to tackle this er: this level students, so therefore I don't feel 100% guilty but nevertheless, I will--I will try to make sure that my erm: you know the--the it doesn't happen most of the time. I--I have to let them move from one level to another. Another--another words it means that ok, in the beginning er: in the beginning of the semester, I'll--there'll be quite a number of code-switch. Then, er: when I move to--with the time, so less code-switch should be done. So, I: because I want them to not only learning English or practising English by using--I don't want them to be exposed too much with their 1st language.
A: I see: you think they will understand better and benefit if you use code-switching
B: Yeah
A: To certain--to certain extend, right?
B: Certain time, it's not would be all the time. So therefore I don't feel that guilty to code-switch. I'm not like all the times using er: keep on code-switching the language. Just only for certain time or certain situation.
A: So do you consider yourself to be proficient in English?
B: Yeah, ok
A: Or else you are not teaching English, right?
B: Ok, as a lecturer, what do you think are your strength?
A: Ok, after being teaching for 11 years, my strength that I would say that erm: I--so far when I find my students were able to achieve their confidence level in using the English language er: they--they'll be able to speak in front of their friends, so er: by through--through the practices that I've done with them er: and I feel that my strength is that I'll--they'll be--they'll be able to become more confident is when I'm giving them the confidence to speak. So my strength that I after been teaching about 11 years, I'll be able to make them aware that by using the English language, it doesn't that you are erm: you are--you know--to--to motivate them. My strength is that I--I--I'm able to motivate them. Erm: that's the thing.
A: So you're talking about strength, what about your weaknesses?
B: My weaknesses, erm: there are a lot. Ok, so when you talk about teaching, ah: yeah my weaknesses would be looking into--into how to tackle students which is er: good. There are consider as good., is very challenging. Er: and--and those who are nt good in the same class. Ok, mixed ability is very challenging so I find to certain extend that I'll not be able to cope er: that is my weaknesses. My teaching strategy.
A: So what do you think er: the steps in order to improve in your teaching to overcome your weakness?
B: Oh: from time to time, I need to like to always update myself with the current technique of technologies yeah especially as you know that nowadays erm: by using quite a number of software that attract them ok, they--when talking about English, they'll need these technologies to be in the classroom. If not, they'll either whether they are good or er: you are talking about this mixed ability students, they'll--they find that teaching would be kind of boring. So when that thing happen, so our purpose of letting them to learn the language will fail. So therefore erm: for that I"ve taken an approach by going through or learning new techniques, new erm: software that can be used in the classroom. E-learning so on and so forth.
A: So, e-learning is still on here?
B: Yeah.

A: Alright, that's good to hear. So, saying that you have been teaching about 11 years, right? So I'm sure you can see the difference between the 1st year that you are teaching until the 11th year now, how do find English language in general?
B: Erm:
A: In polytechnic or even in Malaysia in general. So how do find it…is it becoming…
B: Better
A: Or it's up to you. How do you:
B: So far, after being teaching about almost 11 years, when talking about students' polytechnic level, erm: yeah, they have improved. I think this is the effect from the PPSMTI that they have done in school, ok. They have done something and is er: we can see when they enter erm: example like PUO or polytechnic system, these products of PPSMTI they'll be able to learn the language er: it doesn't--I'm saying that for example. Previously, the students are--they have a very low confidence in using the language, but now after the PPSMTI, you can see that when they er: enter the university er: polytechnic or university level, I think, they're--they're level of confidence has increased and they are more of err: they are better much much better compare with before PPSMTI. Ok, so when we approach them, in--in the classroom, they are--they are erm: their ability can be seen and their readiness to learn also can be--can be seen altogether.
A: Because of the English better, do you think that before this you might code-switch more compared to now?
B: Yes, that's true. Ok, before this, yeah it's true.
A: Alright.
B: It's start from their schools years *la*. Whatever things they've done during their school if they are strong enough then when they've moved to the higher level, so they will have a good proficiency but if it doesn't if they don't have a good strength er: a good platform or their proficiency is low er: in schools, so the result be seen especially in er: when they enter the institutions. So previously, I've--I have to admit that lots of code-switching. But now, ok, I'll say that the number of code-switching is lesser…lesser compared to the previous.
A: So meaning, you are using code-switching more of--on the weaker students not on the er: better students.
B: No. It depends--it depends on the situation ah: code-switching happen because it depends on the situation.
A: Ok.
B: Ok. And also the activity that we're doing.
A: Alright.
B: So let's say if we are doing a group work, ah: less code-switch because the students are discussing. When they discuss among themselves, so they'll be able to understand better when their friends explain but when the activity for example like individual task, er: or pair work sometimes, we'll mix erm: most probably there will be time that we put them together with those who are also weak. So therefore, the--it will come to a point that er: these students have to--I have to code-switch , so that my lesson can be achieved.
A: Ok. Alright. Thank you so much for this interview. So if you have anything else to add or clarify, you can contact me further.
B: Ok
A: Alright, thank you so much.
B: Ok. (13:21)

ii) PUO Lecturer B Transcriptions

Transcription of Classroom Observations

Recording No.: 4
Date: 9/9/2014 (Tuesday)
Time: 10.00-11.00am
Class: DKB 5

No. of Ss: 25
Level: Mixed ability
Age range: 20-22 years old
Topic: Job Hunting Mechanics (Cover Letter)

Notes:

T:	Lecturer
Ss:	Students
(.)	short pause
(0.2)	2sec pause
[	overlapping speech occurs
↑	rise in intonation
↓	drop in intonation
--	change of the topic/rephrase/repeat
::	elongated speech/ stretched sound
=	latched speech, a continuation of talk
( )	words spoken here were too unclear to transcribe
(( ))	transcriber's description
Ø	no talk, ambient noise
U N	capital & space for acronyms
red	Malay words
' '	Sentences read from the board/book/reading materials

**Based on Jefferson, G. (2004)*

((T had small talk with the students before starting her lesson - 00:00 to 00:40))

T: How many of you did the cover letter?
Ss: Everybody
T: Last--Thursday, what did I say? What did I say last Thursday?
Ss: Ah: you explained about the advertisement, the
T: See. I haven't--I haven't gone into cover letter yet. I just explained on the resume, right? Resume, advertisement. (0.3) Did I do anything on cover letter?
Ss: *Sikit.*
T: What did I do?
Ss: Er: er:: introduction, instructions (   )
T: That's all part of the instructions, right?
Ss: Yeah
T: That is just instructions. I was explaining
Ss: We are so advance
T: So, hold on first, because you need to know how to do the cover letter.
((Lecturer getting ready with her slides and to start her lesson - 1:55 to 3:30))
T: Class, are you ready?
Ss: Yes
T: So er: I'm going to give something--I'm going to give you something good on cover letters. I think I mentioned this last week. A cover letter is as important as a resume. In fact, a resume, you should accompany with a cover letter. You cannot just send a resume with your job ad. And the cover letter is actually like a shop assistant (0.2) when you want to a buy a product, a very complicated product, let's say a hi-fi set, you don't know how to--you don't know how it works. So you need a shop assistant to explain. Give

you brief explanation on the er: features of the product. So without the shop assistant, you may probably don't even know which one to buy. Which model to buy or which one is good. So, the…the resume is the product, ok. And the cover letter--the shop assistant is the cover letter. So the cover letter highlights the important features of the resume, ok? So that's why a cover letter has to be: (05:00) done has to be written well. Because we know that if you don't write a good cover letter, most probably er: the employer the potential employer may not want to look at your resume because you have given a bad impression, ok? Ok, let's look at some of the things about cover letters. (0.6) Er: what is cover letter?
Ss: Cover letter explains the product
T: Cover letter expresses your interest in the job and in case the qualification er: reveals your qualifications for a position to a prospective employer. The word prospective means the one you are going to work for if you get a job. Prospective. So:
Ss: Company
T: Yeah, it's actually a letter that says how much you are interested in the job, ok. And this cover letter will accompany your CV. (0.5) Since it is a very important letter, which should it accomplish? Er: .as I said, it should highlight or introduce the main points of your resume. The main points. The resume is full of the tips--information. The cover letter will just extract some important points and mention in your--in the cover letter. It should also help you to sell your qualifications. It's like a promotion isn't it? Sell promotion to the prospective employer. (0.2) Ok, what should be in the cover letter? Er: the header or the top part of your cover letter, it should address it to a specific person, means you need to find out who is this person you are going to write to. Sometimes it's just given as a Human Resource Manager. It's fine. (.) I mean it's fine if you just address it to a person by its designation or title. But if you want to be more personal then you--you can phone the company and find out who is the Personnel Manager your are writing to, ok? Do you think it is er: necessary?
Ss: It's more beautiful when you put
T: More beautiful? More personal, right?
Ss: Yeah
T: Let's say if you received a letter with your name in it--on it. Do you feel like this person knows you, takes an effort to find out your name. So that will give you--make you different from the other candidates. Ok? (0.2) So try to do that in your real life. (0.3) Ok, what is the first thing you must do before you write the letter? When you see the advertisement, what's the first thing you must do?
Ss: Position of the:
T: Yes, you need to some kind of research. Yeah, you need to find out the background of the company. What else?
Ss: Position--position
T: You need to find out about the job itself. What kind of job you--you're going to apply and the job scope if possible. Some advertisements they mention the job scope. It's good to find out more about it. And then what else?
Ss: Qualifications
T: Yeap, what other qualifications that are required from you and what skills. Qualifications doesn't mean just your: like paper qualification like your Diploma. Maybe there's more than that. How much of work experience should you have. What are the skills and strengths that (.) they: are looking for. And all these can be--can be found, I mean some of this information can be found on er: (0.3)you can actually if you can get the companies' reports (.) or you can get some of the newsletters, magazines. You know some companies produce their own magazines. Then or now of course with

the Internet, you can just click er: you can just go to Google and check on the company's background. So it's very easy. Those days I think it's difficult. (0.4) Ok, so we go straight to the introductory paragraph. Ah: just like essay, the first paragraph is very important. You should try to grab the attention of the reader. Who is the reader?
S: The company, the Resource Manager
(10:00) T: Human Resource Manager, the one who's going to read your letter. So how do you grab his or her attention?
Ss: Interest, strength
T: Tell me about your strength.
Ss: Er: strength on qualification.
T: Another words, straight away tell him about your--what you have to offer.
Ss: No
T: Then?
Ss: Er: interested
T: Tell them what interested you are in the job. Why you are interested
Ss: Tension--tension.
T: Don't tell how you feel, what potential that you have. Ok, yes, you will tell them everything that you have said in your letter but the first paragraph?
Ss: Interested
T: That it is stimulate interest, then why you are--you are appropriate for the job. Ok, and then make it clear to the person being addressed. Make it clear as why are you applying--why are you applying. Most probably you are applying because you are really interested in the job or in the company.
Ss: To develop skills
T: Yeah, that one will come in the second paragraph, ok? So the rest of your letter, I would look at it. (0.4) Before that, let's look at what is a solicited application letter. You know that there are two types of letters? One is called solicited. The word solicited means, letters which are written in response to advertisements. That means, this company has advertised on things (0.2) this company has advertised the vacancies and then those who have seen the advertisement, will apply. So, this type of letter is called solicited, ok? And er: so if you are--if you are writing in response to an advertisement in which you are going to do (.) for your assignment, then we need to mention where you learn about this company, where you saw the advertisement. You need to mention that in your letter, ok? So can er: can someone read these examples? (0.6) Who would like to read? I believe=
Ss: =I believe that my knowledge of investment banking and my good communication and leadership skills make me a strong candidate for the position of Finance Executive that was adverti…advertised on Jobstreet.com last Saturday.
T: Yes, so this can be an opening--the first paragraph for your solicited letter. Why is it called solicited? Because (.) why? Why--why--how do I know that this is er: er: paragraph for solicited letters?
Ss: Mention about the--where did it advertised.
T: Yeah, where you see the advertisement., Jobstreet.com. Last Saturday, if you are writing today last Saturday, will be just a few days ago.
Ss: Before 10.30
T:Yeah, you must have a date for your convenience. You can also mention the date on the--ok, that of course er: of course we also have the other of letters which is called unsolicited letter which you will write later when you graduate and er: you find that how come I've never seen this kind of advertising. So, you--you need to take the first you need--you need to be proactive and write to company as many companies that you…you

would like to work with and write this kind of letter that you are going to write for unsolicited. So the company did not--the company does not--does not advertise. The company does not say that once you would like to work again. But you write and ask them. Ask them whether they have any job or any vacancies suitable for you as a polytechnic graduate. So--so unsolicited application letters are written to companies that have not posted job advertisement. Ok, so it is important to gain the readers' attention and persuade them that you can contribute to the company's boost. So, this a letter of persuasion where you need to persuade, you need to convince the (15:00) person, that in case, there is an opening, please consider me. Please look at my letter. So, most probably if you are a potential candidate, the company thinks that you are potential, they will K I V you. You know, they will keep your letter and then once there is a vacancy, how do you--how does the company has a vacancy? (0.4) Tell me. Like for example, now they are full, they have enough staff but when--what situation is it they can have a vacancy?
Ss: They want to open a new branch
T: Ha:when they open a new branch, what else?
Ss: Retired
T: When someone retires, but that person must be--not--not a new experience, old, someone maybe not retired.
Ss: Fired
T: Fired? When someone is fired.
S: Fired *hampir macam*...
T: Ok, when someone resigns. (0.3) Then, there is a vacancy. What is vacancy? *Kekosongan*. So from there you ah: you might be considered the job. So that's why is important to try to apply, I mean not apply, to write letters to many companies upon graduation. Otherwise you completed your studies, it's good to ah: take this step instead of just waiting for companies to advertise. Ok, can someone read the example?
Ss: As a member of one of the fastest growing banking organisation in the world, you have a company that is established. I'm a recent polytechnic graduate with as specialisation in banking & finance & budget analysis areas.'
T: Yes, so this is a question isn't it? But in the question, you'll find that there are a few things mentioned here. This person knows what kind of company it is ah: it is a member--this bank is a member of one of the fastest growing banking organisation in the world. It is a very established company and then er: he wants to work in the analysis department, he is a recent polytechnic graduate. He specialises in banking and finance and he has some experience in budget analysis. So, all inside, right. So many in.
Ss: In finance
T: Yeap. So, this can be really catchy, isn't it? Do you have anything for me? (0.3) Of course there are many ways of writing. Any questions so far? (0.2) So, you already learn that--there is such things as solicited and unsolicited letter. What you are going to do is writing a solicited application letter, not the unsolicited. (0.4) Ok, remember er: after the introductory paragraph, you have the er: we called it the body paragraphs. So, what did you write in the--the 2--next 2 paragraphs?
Ss: Work experience
T: Yes. You highlight your work experience plus your qualifications, anything else? Maybe your achievements, your skills, your strengths, all these can be written in the next 2 paragraphs. Skills, strengths, work experience, qualifications, special project, achievements. Achievements can be if you are the: top student and what else? If you take part in a project, and it is very relevant to the job that you are going to apply, use them. Highlight the strongest qualifications for the position for which you are applying.

Strongest qualifications. Of course here you don't mention your SPM anymore ha: Diploma is your highest qualifications. Whatever that is--that is relevant to the job, you should highlight it and another thing is you must refer to their advertisement. In an unsolicited letter, you actually do not really know what they want, but in er: solicited, they listed for you what kind of skills they are looking for. You should, what kind of strength er: you know strengths, what kind of personality. For some jobs, you know, they want someone who is very friendly you know, especially like customer service, someone who is pleasant. So, go back to the advertisement. Look for the--(20:00) look for the requirements in the advertisement. (0.3) This advertisement is very important ha: so that--so that you still find that you are the right person. How to know whether you are the right person?
Ss: Requirement
T: Yes, in the requirements. (0.2) First of all, you have a Diploma in Finance & Banking (.) from a recognised institution like from Ungku Omar Polytechnic. And then secondly, what is it? (.) We are able to evaluate?
Ss: Soft--problem solve
T: It can be your skills. Ah: is it soft skill or hard skill?
Ss: Soft skill, both
(0.2)
T: Ah: the second one, is it soft or hard?
Ss: Soft, hard
T: Yes. Probably this job requires you to--you need evaluate, you need prioritise and solve problem. Ok, so it is part of the job. So if it is part of the job, it's a job requirement, then it is a hard skill. And then the third one, good in time management (.) hard or soft?
Ss: Soft
T: And able to meet tight deadlines. So this is talking about time management er: punctuality and er: able to meet deadlines. That means you do not procrastinate. You are not the type who put on hold
Ss: Last minute
T: Or last minute kind of worker. Are you? Are you a:
Ss: Yeah, sometimes (Ss laughing)
T: In this job, you cannot afford that you cannot say ok 'never mind', the boss will understand. You know, you cannot say that ah: you need to be on time in any project. Erm..,the fourth one. Excellent interpersonal skills and able to work well with all level of people. Under categorised under? Soft, isn't it? Anything to do with people is considered soft ah:. So, excellent that's the keyword. Not just good but excellent. So do you--do you think you have excellent interpersonal skills? Some of you may have, but how about able to work well with all levels--all levels not just certain level, your colleagues but all levels. From the top management, right down. Can you? So this type of er: requirements, are equally important, as important as your Diploma, ok? Computer literate. Computer literate means you need to know how to use the computer. The advertisement does not say what kind of er: program or software you must have but at least you know the basic. If they--if it is the requirement, they would mention the name of the programme. But I think basically, er: you need to know Microsoft software (.) and maybe some of the banking software. (0.2) Ok, and the last part, before employers do you--how do the employer get you, you have enclosed the resume. (0.5) Any questions?
Ss: No

T: Look at your letter since you have done the letter. Ok, look at the letter and see whether er: is done correctly according to the--why are you keeping it? Correct it now? You want me to correct it now
((Ss laughing))
T: So special. I will do it--I will do it. So, but, look--look at it ah: how many pages--how many pages?
Ss: One, one
T: You adjust--just one page. I'm sure you can do it.
Ss: (   )
T: Let's continue. Detailing your experience means? (0.2) Detailing your experience is? (0.2) Providing details for your experience ah: working experience. Ok, the first one, show in bracket, don't tell ah: don't just tell but show the employers your qualifications. In other words, prove it ah: what--what er: what support your qualifications with proof or evidence. (25:00) Includes specific credible examples of your qualifications ah: So, the keyword here is the:
((Ss knocked on the door and came in late))
T: Where were you?
Ss: Print
T: Oh…we have a guest. (0.5) Where did you go?
Ss: Printing letter
T: Come again
Ss: Printing letter
Another Ss: She print out the letter
T: Oh: you went to print? Not necessary actually. (0.3) Ok, it's ok. But don't be--next time, let me know first ah: before you go out. Ok, er: includes specific credible example of your qualification. Meaning that, be specific ah: when you mention your experience or your qualification. You've done? Don't put it in a general sense. Try to be very specific everything, ok? So, how to be specific? Use numbers, names of equipment. This one is for engineering or names of software, banking software that you have used or features of a project that you have applied to the job. So, remember this. We do not want general statements: in the letters. Try to be specific. Ok, let's look at one example. In this example, so far I tried to cater it to your: banking and finance skills. But of course there'll be one or two who's er: in engineering field. Ok, can you read it? (0.5) Sharifah. Louder--louder. Louder please.
Ss: 'As a banking representative at a bank, I provide
T: Provided
Ss: provided quality customer service while doing the sales of product to customers. I also handled outwards 20,000 a day and was responsible for balancing the bank'
T: Yes, so, can you see? What so er: special about this?
Ss: Specific about handling the:
T: She's responsible.
Ss: Has experience in banking
T: She has experience?
Ss: From working
T: Ok, now let's look at the first one. I provided quality customer service--I provided quality customer service.
Ss: Soft skill
T: Yeah. But what is the: difference about her sentence is over quality.
Ss: Best

T: Everyone in this line will provide customer service, but she mentioned it as quality. That means she is set a--she's a
Ss: High
T: High standard person ah: whatever she does, she will er: make sure there is quality, ok, while promoting. So she--while promoting, she can also mean that she's doing two things at one time. So, you can say that she's multi-tasking. Multi-tasking is?
Ss: Can do two work in one time
T: Ok, yeah. She can handle two things or more than two things yeah. 'I also handled--I also handled outwards 20,000 a day and was responsible for balancing the bank.' So you see, she's a very capable person. This is talking about your experience. From the experience, you can deduce and say what kind of person she is, what kind of worker. If you are, I believe that if, as a student if you are the responsible type, you are also be responsible in your work. This kind of--this quality is don't change, as a person. Ok, any question? So remember your work experience need to be specifically detail. Cannot be too general. (0.4) Using active language, er: what are the don'ts yeah. Don't be vague. What's vague? (0.2) Ok, don't be vague in your description.
Ss: Over
(30:00) T: Over what?
Ss: Over describe
T: Over describe? Don't be vague. (0.2) 'I worked as a forwarding agent at Air Asia.' Is that consider vague? Vague means *kabur* you know *kabur*? Vague
Ss: Oh:
T: So, 'I worked as a: forwarding agent,' although it is true, 'I worked as a bank executive or whatever.' It's true but what do you do? What do you do as a forwarding agent ah:? Or is very general. Not specific. Don't use weak verbs. Such as endeavour, tried, hoped and attempted. So, all these words are also vague. They are--they are not specific. They are not er: active. They called it you know, it's just like I hope--I hope, I tried or I attempted verb ah:. So, we are attempted to attract customers. How did you attract? How did you do it? That's what we want to know. (0.2) Ok? You use strong--I mean, you use active verb. Don't use
Ss: Sexist
T: Sexist language such as chairman and manpower, ok. I don't know whether this is applicable to you er: in letter writing. Here, erm: it's good not to use sexist language. That means, don't offend another sex. Another sex can mean the: in this case the women. Do--would you say the chairman? Don't know, you said. It's not fair you know, there are people who do things are women.
Ss: Oh:
T: Why you use the word chairman?
Ss: For men.
T: Chairwoman isn't it, instead of chairman. Why not you just use the word chairperson?
Ss: Chairperson
T: You see, all will be very happy because is not sexist. And manpower--manpower.
Ss: Human power
T: Can you say womanpower? Cannot. Because the word manpower is a--a word that means a the--the stuff, the manpower the word done by human. So what can you say? (0.5) Ha? Human resource? Ok, in another word, don't use sexist language. Don't er: don't try to offend other people especially the weaker gender. (0.4) Ok, what other use then, just now was the don'ts. Use concrete verb to describe your experience. Concrete. That's the--to differentiate from the weak verbs. Er: use present tense. Ah: this is very important, because your letter is a description of your experience and so on. If it's a past

experience, then you use past tense. If it is a present working experience, that means you are still working, make sure you use all present tense. Erm: be as specific as possible in the description. List dollars, amount and figure as you can. As I said, figures are very important. Numbers are very important. Ok, let's read the vague one. (0.3) Would you like to read?
Ss: 'I worked as a calling agent at the'
T: Calling
Ss: 'As a calling agent, I assisted and oversaw things to confirm the result. I stopped when the industrial training is finished.'
T: Yes, Can you see? 'I worked--I worked' is past tense. He's no longer working there ha: as a calling agent. And then, when you describe, the same thing you need to er: I mean the 1st one is too vague. Then you need to improve it
Ss: Job scope.
T: Yeah, improve it by telling what you actually doing as a calling agent. 'I assisted' the past tense 'assist', 'assisted'. 'Oversaw', oversee is the
Ss: 'Stopped'
T: basic word and 'stopped', 'ed'. So check your letter and see whether you use past tense you know, for industrial training experience. Ok, have someone to read the next one. Er: Nor Syed? (0.6) the second one, 'I attempted
(35:00 Ss: 'I attempted to attract customers' wrong. 'I initiated a programme to attract customers to Pizza Hut. Resulted in a 5% increase of sales for the month of June.'
T: Yes. So, can you see the difference? 'I attempted to attract customers.' Is very vague ah: What do you mean er: you attempted. What did you do--what did you do to attract the customers? So, the strong one would be, 'I initiated a programme,' that means, he's the one who's--who acts--the idea comes from him you know, he tells the boss, why don't we do this. He has a great idea. So, he initiated a programme to attract customers Pizza Hut and what is the result? Just to attract customers but the end result is there was a 5% increase in sales for the month of June. So, doesn't that show that er: this person is you know is not the same--not ordinary. He has a lot of ideas and then he can initiate. A lot of initiative. So, show that in your letter. (0.2) Ok, show what you have, what you have done in the letter. (0.5) Ok, organising your letter. So this is er: (0.2) in general.
Ss: 1 page is not longer
T: Is no longer than 1 page. Anyone has more than 1 page? (0.4) That's why, class, you are so advance.
((Ss laughing))
Ss: Yes, we referring by this book.
T: Which is good, you can do on our own but you still need check whether you are writing the right thing. Organise your body paragraph, emphasise your strongest (.) and most relevant qualifications. Make sure that you highlight ah: you emphasise your strongest and most relevant. Only include 2 or 3 strongest in here. As I said resume--your resume has a lot of things, only take 2 or 3 important (.) points. Make it easy. How to make it easy for your reader to read your letter and know what you have written? Use?=
Ss: =Simple sentences
T: Topic sentences. You know what is topic sentences?
Ss: Yeah
T: You have topic sentences in yours?
Ss : In here
T: That is subject heading.
((T read one of her students' letters)).

I noticed that is not already have a topic sentence. I mean this letter I read. So, a topic sentence actually is a sentence at the beginning of a paragraph that tells (.) what a paragraph is about. Why you make it a dot dot? If you are going to talk about your qualifications and experience, and mention that in the 1st line of your paragraph.
Ss: I am writing to
T: That one is introduction.
Ss: Oh I see, you have to mention the (.) main points
T: Yes, you have to tell (.) in the topic sentence, what your paragraphs about. Or (.) the topic of your paragraph.
Ss: Topic?
T: So ah: if you are talking about skills and strengths, then mention that in the topic sentence. Ok, you need to re-write it. Re-write your letter. Actually, (0.5) this topic--this word topic sentence is not er: nothing new. We always er: use in essay writing especially for MUET, you know, we always tell the students, that we need to have topic sentence. What--it actually, it doesn't have to be at the beginning of the paragraph. It can be somewhere around, but normally is at the beginning. So that from there the reader can--they are guided. Oh: this paragraph is about (0.2) for example, it's about the effect of something. This paragraph is awareness of something. Then only you give the (.) details. Ok? (0.3) Ok, how do you conclude the letter?
(0.5)
Ss: Provide the summary
T: I'm sure your letter and resume is not just for them to read and see only. It defeats the aim (40:00) for writing the letter. What's the objective? Class, why do you write a letter?
Ss: To get a job, to promote
T: To promote yourself and then that's it. Finish?
Ss: For the (.) job
T: Because you want to be called for an interview and most job require er: you to attend an interview. Most jobs. So that's why you write the letter to convince them that they are the (.) potential candidate for the job. So, but then it's still not over yet ah: going for an interview doesn't mean that you will get the job. (0.2) It's just the next step (.) to get a job. But it is an important steps because there are people who will fail the job interview. To the--if you don't receive any reply from them, it means that you are not=
Ss: =Qualified
T: Qualified for the interview. Ok, let's look at how you can conclude your letter.
Ss: Provide your contact no., your
T: Can someone present that section? Ask your neighbour. (0.3) What's his name? Simpson?
((Ss laughing))
Ss: 'I would welcome an opportunity to discuss my qualification with you if you are interested. Please contact me at 017502602 before 11am or feel free to leave a message.'
T: Yes, where to leave a message?
Ss: Fax, WhatsApp
T: So this is actually a modern day ah: before this, I don't think you can write 'leave a message'. Now with the handphone, with your handphone no., they can leave a message. Ok, so 'I would welcome an opportunity to discuss this.' I supposed earlier on you have said something. 'And other qualifications that you (.) are interested. I you think that I am the right candidate, please contact me.' Can you see--do you see any word er: anything about interview in that sentence?
Ss: No--no

T: So there are many ways for asking for an interview. You can say erm: please grant me an interview and you know or you can say
Ss: Discuss my intention
T: Yeah, the word to discuss means you want to meet face-to-face. (0.3) Ok, is it ok to tell them when you are free?
Ss: No
T: 'Contact me at, before 11am'. Is it ok?
Ss: No, before 11am *tu macam berahsia sikit*
T: If you don't tell them, what if they contact you when you are having a meeting or a class or:
Ss: It's ok
T: It's ok isn't it. It's just that any time before 11.
Ss: You can leave a message
T: To leave a message. So, it's good to give specific instruction to the employer so that they know what to do (0.2) and the won't *kacau* you.
Ss: Sorry *kacau*
T:It's not nice to be interrupted in the meeting. Let's say you are the person--the person chairing the meeting--a meeting and then there's a call from the company, it's not nice isn't it ha:? So be specific about how the interviewer should contact you and say thank you. (0.3) Erm: (.) this one is about the--the design. Actually, there is no--more than one. It says coordinate the design of the letter with the resume. That means, your resume and cover letter should have the same design. If you are doing some design. If not, then it's just a normal cover letter. Some people put some design in the resume so the cover letter and the resume can be the same design. Simple design. (0.5) What are the key points to remember, the last part?
Ss: Elaborate information
T: Make sure you (.) elaborate, elaborate means?
Ss: Tell about
T: Provide more details ah: instead of just making general statement. Evidence of your qualification? To provide evidence meaning?
Ss: Certificate
T: No--no it's not--it's not about enclosing the certificate. No, it's just that when you (45:00) mention that you have Diploma, can you tell more about your qualifications yeah.
Ss: Subjects
T: Probably the relevant subjects or something about your: achievements. Proof-read. This is very important, when I look at your letter, I just have to have one look (.) and I can see all the mistakes.
Ss: Oh no: *takutnya:*
T: I have able to evaluate--I have able to evaluate--I am able to--'I am good at time management.' (0.5) 'I am proficient in variety of computer literate.'
((Ss laughing))
T: Look at this! 'I am proficient in variety of computer literate.' Er:
((Ss laughing))
T: What's that? (0.6) And 'able to deal with client.' One client?
Ss: With 's'
T: There's no such thing as the finance field (0.3) in the finance field
Ss: Financial
T: Cut and paste?
Ss: Eh no!

T: Copy and paste? Same mistakes!
Ss: No!
(0.5)
T: Ok, actually if you have copied it from somewhere, make sure you copy properly.
((Ss laughing))
T: Don't pick up the mistakes. Ok, this sentence 'I very much hope--I very much hope you will invite me for an interview'. (0.3) 'I will--I very much hope' why must you very much hope? 'I hope'
Ss: *Maksudnya sangat berharap*. 'I hope you will invite her'
((Ss laughing))
T: Most of you actually, most of you--forget your this small letter. Find a letter which is very important. You know that?
Ss: 'a'
T: Article 'a'. A lot of you don't realise that you need to put it, when necessary ah: for example, industrial training. (0.3) Er: 'I have done--I've done 4 months industrial training.' You must say 'I've done'
Ss: 'a'
T: 'a 4-month'. (0.3) 'I've undergone--I've undergone or I've done'. But that 'a' is very important
Ss: Month not months?
T: Why because training, (.) we are describing the training 'a 4-month training'. Later, I will check. So, the aim of writing--the aim is to make your letter error-free.
Ss: Yeah
T: Error-free means I cannot even find one tiny mistake. So that is your aim ah:. And check for typo mistakes. Typo means, glaring ah: not tiny mistakes.
Ss: Check mine
T: I need to check some, I need to check. So, what I want you to do is er:
Ss: Pass up
T: No, I want you to hand-in now, I want you to make your partner look at your letter.
(50:00) Ss: My partner always same, copy!
T: Look, not only for (.) er: content, format--format, content, language. Language part you can check for grammar mistakes. If you notice any mistake, please correct it or highlight it. Circle it. Can you do that?
((Ss continue checking their partners' letters - 50:30 to 51:16))
T: Ok, can you exchange it with your partner? (51:23)

Transcription of Interviews

Recording No.: 5
**Notes:*
A: Interviewer
B: Interviewee

A: Good afternoon.
B: Yes, good afternoon.
A: Thank you for agreeing to talk to me. Ok, as you know the purpose of my research is to investigate the use of code-switching among the English Language lecturers in the

Malaysian Premier Polytechnics. Therefore, I would to have some views on your own practices in the class and how you use code-switching in the classroom. So, it will be confidential and only be used for his research purposes.
B: Ok.
A: Ok, I'm sure you have herd of code-switching before, right?
B: Yeah.
A: So what can you understand, or what did you know about code-switching?
B: Ok, er: I think code-switching is when you switch to another language while you are using one language, one particular language and then without realising it or…unconsciously you just use another word er: I mean, a: either you explain the word in other language or you continue the sentence with another language.
A: Ok, so did you notice that you code-switch during the lesson just now?
B: Yeah, I did: a few times.
A: And based on my observation, there were only a few code-switching.
B: Aha:
A: Why do you think you do it?
B: Ok, the reason why I did it is because err…because I wanted the students to understand the word that I used for example 'vague'. I find that when I asked them what's the meaning , they didn't understand, I mean they couldn't give me the right meaning. So, for I think the best way is to tell them in Malay so that they can get it.
A: So do you, was it a planned one or an unplanned strategy that you used?
B: It's not planned. At that time, I just er: wanted them to understand it fast.
A: Ok. Is there (.) any other alternative that you could think better rather than code-switching at that time? So do you think code-switching is better to do it during that time?
B: E: maybe at that particular time I was I remember the word in Bahasa so I just used it. But if I can explain it, er: in English, I would. But I think it was bit difficult to explain…in English.
A: Yeah, and it can actually saves time, is it, you think so?
B: Yeah, save time.
A: Ok, so what is your 1st and 2nd language that you used normally? Your 1st language will be English and…
B: No. Ok ((clearing throat)), at the moment, I use English most of the time with my family, but before this before I (.) actually started a family, I--I also used Malay with the--my own family.
A: I see, ok.
B: But, I still use er: Malay when I go back to my hometown.
A: That is still consider as you 1st language?
B: That's my 1st language. That's my mother tongue.
A: Ok. So what subject are you teaching here?
B: Oh: I only teach English.
A: So, how long have you been teaching?
B: I've been teaching for 27 years.
A: Wow! Ok, that's a lot. So most of the time, you use English?
B: Yeah. Because since I started teaching, I--I don't think I've taught any other subject apart from English. Maybe just a once or twice short still on Moral Education.
A: Ehm: do you think that what you did like just now, code-switching will benefit the students or actually it will have a negative impact on the students?
B: Erm: I think it will benefit them because er: benefit them in the sense that it will save time for me to explain but er: if they expect me to teach everything in English, it won't look good on me. But, however I think my students don't mind if I code-switch.

A: Do you consider yourself to be proficient in English?
B: I think so
((Laughing))
A: Alright, as a lecturer what are your strengths?
B: What about my strengths?
((Laughing))
A: Overall, generally.
B: Related to teaching?
A: Yeah.
B: Related to English?
A: Ehm: English teaching or teaching whichever subject
B: Ok. I--I still have a--I still love teaching even after so many years of teaching. So, when I teach, I probably put my whole heart into it and I care for the students whether they learn whatever I'm teaching, they gain something from it. So, probably I can say that er: I'm quite, I'll make sure or I'm quite responsible in making sure the students gain something from it. And, I--I'm quite serious about you know, doing this. Maybe you can say I--I teach with my heart.
A: Auww: that's nice.
B: I do care for what I do: whether the students gain something.
A: Yeah. Because I can see some lecturers they would rather consider finishing their syllabus rather than their students' understandings, right? So you are putting more of your students'
B: Interests
A: Interests first rather than yours, right? Ok, so you have your strengths, of course you have your weaknesses.
B: Yeah
A: Ok, what do you think are your weaknesses?
B: Ok, my weakness er: my weakness, ok physically I think my voice is the weakness. A lot of students have complained, a lot of observers also complained that I don't have a loud voice to er: that's why because of this, I find students tend to er: lose concentration and they start to chit-chat. They start to do some other things. And I'm also quite soft-hearted, so that makes me not a good, I mean is not good (.) I find it as a weakness because (.) they sometimes they are not serious in their work because of me. And I'm not very stern, very strict on them. So I think that I can--if I can will be stricter with them. Probably they will listen, they will do their work better than what they are doing now.
A: Ok, what do you think of English language in Malaysia in general? Do you think the level is good or?
B: Well er:
A: Is it deserve to be a second language or....anything, that you can find. Is it important?
B: Ok er: I feel that it is more important now than before. Although I--I can sense that I'm not sure whether the students' language--the students' proficiency has been improved. I'm not very sure, but I can see that in some ways they have improved that er: where er: vocabulary is concerned, they know more words than before partly because some of them have done Math and Science in English. They are more exposed to new terms and all. But in terms of speaking, er: speaking communicative ability, I still feel that it is almost the same as before.
A: I see

B: So the main problem--the main er: I mean if you want to improve we should concentrate more on their communication skills, I mean their speaking. Because the--the in terms of word power, improve a lot compared to those days.
A: Not enough practice I guess.
B: What was the question again?
((Laughing))
A: How about English in general?
B: Ok. Generally I think they have improved. Generally.
A: In terms of, I mean vocabulary and all
B: Yeah
A: Ok. So do you think that (.) you code-switch before more compared to the students in the new intake or is it more or less the same?
B: More or less the same.
A: More or less the same.
B: I code-switch not because of their proficiency, is weaker now. It's just because I think at that time I can use another word to explain better. So that's the
A: Ok. So that they understand.
B: So those days I also code-switch for this same reason.
A: I see. So that it doesn't matter whether they are weak students or (.) you could still (.)
B: I supposed if the class is weaker that means they don't really--I mean there are some classes which are not. I mean most of the students are not as good as the other class. So if I find that this class, most of them are weak in English, then I would code-switch more often because my objective is to make them understand. So, I would do that. But, so far, I have not encountered a very weak class.
A: Alright, I think that's all. Ok, thank you for talking to me. So, if you have anything to clarify or anything else you want to add on, you can just contact me. Alright?
B: Ok.
A: Thank you.
B: Ok, thank you. (09:46)

iii) PUO Lecturer C Transcriptions

Transcription of Classroom Observations

Recording No.: 8
Date: 12/9/2014 (Friday)
Time: 12.00-1.00pm
Class: DUT 5B
No. Of Ss: 21
Level: Mixed ability
Age range: 20-22 years old
Topic: Job Hunting Mechanics (Cover Letter)

**Notes:*

T:	Lecturer
Ss:	Students
(.)	short pause
(0.2)	2sec pause
[	overlapping speech occurs
↑	rise in intonation
↓	drop in intonation
--	change of the topic/rephrase/repeat
::	elongated speech/ stretched sound
=	latched speech, a continuation of talk
( )	words spoken here were too unclear to transcribe
(( ))	transcriber’s description
Ø	no talk, ambient noise
U N	capital & space for acronyms
red	Malay words
‘ ’	Sentences read from the board/book/reading materials

**Based on Jefferson, G. (2004)*

T: Ok guys, so we are moving on to the 3rd step. You have you er:: what your:: job advertisement, and then you have come up with your resume. And then we are moving on with ↑your:: ?
Ss: Cover letter
T: Cover letter. Ok? (0.2) So what is a cover letter? Again? Ok (.) read in front. A ↑way::
Ss: A way to (.)
T: To tailor qualifications to job opening. Ok, look at the word there ‘tailor qualifications’ in red. So the word ‘tailor’ means (.) what?
Ss: Someone who makes clothes.
T: Someone who makes clothes? In terms of a:: cover letter?
Ss: Specially for someone
T: So which one? A or B? Tailor:: ?
Ss: B!
T: Tailor is a-- a what? Is a you can made ↑of:: =?
Ss: =Especially
T: Actually a cover letter you made (.) especially because you want ↑to:: apply for ↑the=
Ss: =Job
T: Job. Ok, good. (0.2) Ok, so in cover letter, these are the things that you need to include between the job that you want to apply, what is your ability that you have in order for to apply for the job, what are the knowledge that you have. Remember that you have learnt on skills and abilities?
Ss: Yes
T: So basically, these are the information that you need to include, ok. So cover letter is actually:: a what?
Ss: A summary of a resume
T: A summary of ↑a:: resume. Ok, when cover letter complements ↑your::?
Ss: resume
T: ↓resume. True or false?
Ss: True
T: Complement means?
Ss: True

T: Complement means?
Ss: Er (.) (   )
T: Complement means what *pujian*? No
Ss: Certification
T: Ok, you have your resume and you need your cover letter. You cover letter is a complement for your resume. Means?
Ss: To support
T: Ah:: to support ↑your resume, ok? So, er (.) cover letter basically is ↑a summary. You have all:: the details in your resume, but the summary has to come up with your cover letter, ok? Are guys ok?
Ss: Yes--ok--
T: Means only right yeah-- ok
((Ss laughing))
T: Ok, so the objective of the cover letter is ↑to=
Ss: =Generate
T: Generate interest. So that:: ok, er the--your future boss might go through your cover letter and read your details, summary of your qualifications and everything before he move on to ↑your:: resume. So, you create the interest there. Maybe when he did--later or ok--this guy has all the things that I need but maybe I should look more details in his or her resume, ok? (0.3) Ok now we are going on with the tips, ok and how to write a:: perfect--perfect cover letter, ok? Tips no. one, ok? To address the right =
Ss: =[Person
T: =[Person because basically when you go online to Jobstreet for example ok, er you find the article or the job advertisement and the you want to know who should you send the letter to. So you go to the ↑company's:: website to know what actually the name of the person so that you can be specific in your letter. Basically, you want to show that you just actually have the effort to go online and search for ↑the specific person rather than stated there ah 'Dear Mr HR' that's it. Ok, make sure you try to be specific. Who is in-charge of ↑the=
Ss: =Interview
T: Job application. Ok, with that can we move on?
Ss: Yes
T: Ok, tips no. two, to make ↑your cover letter:: ?
Ss: Concise
T: Concise? Consist? Concise? So what does it mean?
Ss: Don't over-write
T: Don't over-write your cover letter. Make sure it's simple but all the information is there. Ok, don't write about four pages. Kindergarten I involved in playing football, and then primary school still football and then secondary still football, tertiary also football. Just make it short and simple. Include only the important information because the rest they can refer back to ↑your:: ? To ↑your=
Ss: =Resume
(05:00) T: ↓Resume. Ok, good. Ok, tips no. three, never::?
Ss: Re-use your cover letter
T: Your cover letter. Basically, er:: most of the students will have this habit. Ok, you are very lazy, ok *malas*--copy paste. That's it. Ah ah:: on top of that '*Persiaran Cempaka Sari*, I am *Mohd* bla bla bla having a Certificate in--Yours sincerely Azida'.
((Ss laughing))
T:Up there '*Mohd*', down ↑there '*Azida*'. Clearly show that you ↑copy and =
Ss: =Paste

T: ↓Paste. So you whenever you want to apply for a new job, make sure ↑you:: make a ↑new=
Ss: =[Cover letter
T: =[↓Cover Letter. Ok? Don't copy and paste. (.) Ok, so what are information that you include in paragraph ↑one? (.)
Ss: To tell::
T: To ↑tell? To HR how you learn about the:: company, which mean-- How do you know there is a job vacancy then?
Ss: ( )
T: Ok? And then, maybe you can say that I find-- I found your advertisement from a Jobstreet. And then, no. two:: it doesn't hurt to say something nice about the company. Ok? 'Through my research I find out you are one of the most leading company in Malaysia.' So, give them some compliments even though it's just a company *cap ayam*. Ok, give them some compliments so that ok this person is interested to ↑work:: with me, ok? (0.2) Ok, in paragraph 2:: include ↑your=
Ss: =[Education.
T: =[↓Education:: qualification. Ok, make sure you have to write your Diploma and then the second one you have to include ↑your=
Ss: =[Previous job accomplishment
T: =[Previous ↑job: ↓accomplishment for example maybe you have worked during your *L I* , er:: you have erm:: you've got er:: maybe er:: the best *L I* student there. You can also ↑include, in ↑the:: cover letter but don't include the--if you kicked out from the company because (.) =
((Ss laughing))
T: =you're sleeping during the-- your *L I* there, ok? Only include the bad-- the good things into your cover letter. (0.3) And then, you need to ↑highlight:: the qualities again, the personal qualities is very important because it shows who you are, ok? And then, ↑highlight relevant professional skills. Any professional skills that you have. Make sure you include that. And then, justify why you are perfect for the job. 'I think er-- I am a very punctual person that suitable for the job as you company starts at eight o'clock' for example, ok?
Ss: Yes
T: Identify--justify why you are the ↑perfect: ↓candidate and then how: you can contribute to ↑the (.) =
Ss: =company
T: Company. Not what company can give you, but what you can contribute to ↑the ↓company. (0.5) Ø Ok, and then leave ↑a positive lasting impression for you and your letter ok, to show that you are actually eager to join the company, ok? And the--and then, draw attention so that the person who read the cover letter, read your resume. Refer to your resume and then:: before you end the letter, don't forget to (tag) ↑the person who read the letter and then ask ↑for interview. Ok? So we have two types of letter. The first one is solicited ↑and unsolicited. What is the difference? (0.3) Kamarul!
Kamarul: Yes
T: Ha: what is the difference?
K: What is the difference?
T: Ha!
K: Solicited::it's clearly stated there
((All laughing))
T: It is!
K: Solicited states why you are writing the letter, the job you are applying for.

T: So, it is clearly started there. So what--what is the difference?
((Ss laughing))
T: Ha: take your time
(0.3)
K: Unsolicited ah:: you demanding on the position.
T: You demanding on the position. Solicited?
K: You ah:: ah:: ah::
((Ss laughing))
T: Anyone would like to help Kamarul?
Ss: Liyana!
T: Liyana, any idea Liyana?
Liyana: Er:: solicited is very simple.
T: Ah::
L:Unsolicited er:: we need--I know--I want the position.
T: Unsolicited you want the position?
L: Ah: it stated the position
(10:00) T: Agree?
Ss: Disagree
T: Disagree?
(   )
Ss: Fikri!
T: Ha: Fikri, do you agree with Liyana?
Fikri: Yes agree enough (but   )
T: Ha? Say again.
Ss: Not sure
T: Not sure? Er: can you give the differences?
(0.5)
Fikri: Ah: solicited er:: he just want to there--there to apply the position. He want er:: he want position in the company.
T: He wants the position? (.) Ah: ok, agree? Anyone has different answer? (0.3) Mus?
Ss: Mus!
(0.4)
Mus: You know about the (.) vacancy
T: You know about the vacancy?
M: Er: that you want to apply
T: That you want to apply, ok.
M: Solicited is (.) you don't--you don't know (what to apply)
T: ok, good. Did you guys get it?
Ss: Yes
T: What is it? Kamarul!
((Ss laughing))
T: Very confident, Yes!
((Ss continue laughing))
T: ah:: Farah?
Ss: *Sekali lagi Mus*
(0.2)
T: Ah: Arshad?
((Ss laughing))
Arshad: Firstly is you know about the vacancy in the company, but you want to know.

T: Ah: you don’t know but you want to apply--you want to ask if there is any vacancy there. Ok? Ok? Ha: so, in your case, you are going to write solicited or unsolicited?
(0.2)
Ss: Solicited…solicited
T: Ha? Solicited. Why?
( )
T: Why:
Ss: Because we don’t know
T: Because you don’t know?
Ss: People and company
T: You know which company and which er--what er: =
Ss: =the position [in the company
T: [Yes, position that you wanna apply, ok good.
Ss: Yes!
(0.3)
((T scrolling the power point slide))
T: Ok, basically it’s almost the same thing:: your highest academic, your relevant experience:: (0.2) same thing::. Ok (0.2) ok! Let’s see if you wanna choose er: what? You want someone who can cook. Which one do you prefer? The first one? Sals--Salsparila or Umi Kalsum? The first one would ↑be? ‘Dear Mr. Omaro, I definitely can cook.’ (.) ‘Dear Mr. Omaro, I enjoyed cooking very much in fact, I have won a cooking competition twice.’
((Ss laughing))
T: So which one you choose?
Ss: Number two
( )
T: Why?
( )
T: So you are interested in er:=
Ss: = cooking competition
T: Because of what?
( )
Ss: Simple
T: Simple? Weak also?
( )
T: So now it’s simple and weak also
Ss: It is good
T: Because of what--but which one that draws your attention? (0.2) Why?
Ss: Impress you
T: Impress you, which means when you write, you have to write to impress, ↑ok?
(0.3)
T: Ah: Rosalinda and Safura
((Ss reading on their own from the power point))
Ss: Rosalinda
T: Rosalinda? (.) Why?
((Ss discussing with friends))
Ss: Humble--humble
T: Humble? Ok, this I Rosalinda. Next one?
(0.3)
Ss: Jamal

(0.2)
T: Ah: (.) Encik Jamal. Another sender, which one?
Ss: Jamal--Jamal--Jamal
T: Jamal, why? (0.2) You have all the detail there, right so that you actually ↑a::? =
Ss: = combination (.) perfect
T: Hmm: a good student there. 'I am--I am smart.' That's it.
(15:00) ((Ss reading on their own from the power point slides -14:54 to 15:24))
T: The left or the right?
Ss: The left
T: The left? Left? Left? (.) Those who say the left, why is it?
Ss: Simple
T: The left one is simple, the right one?
Ss: Over write
T: Over write? So what can you do if you don't want to be too simple and you don't want to be: over write?
Ss: Short and perfect
T: Make it short ↑and?
Ss: Perfect
T: Perfect
Ss: Perfect--perfect, short and perfect. *Padat dapat kerja*.
((Ss laughing))
T: ok, I was active in several clubs. Ok, read. Which one?
((Ss reading on their own from the power point slides))
Ss: Left--left: left—left (0.2) Left
T: How about the right one?
Ss: Less valuable (.) experience (   ) experience.
T: What would be the differ--different between the left and the right one?
Ss: Right one just explain the experience (   ) than the left one
T: The left--left one would ↑be:: different. You just listing thing down (.) what are the things that you join. And then, the right one?
Ss: Er:: talking about experience from the::
T: Ah: between what are the things that you get. So which is better?
Ss: Right one er:: right one
((Ss reading on their own from the power point slides to find the answer and discuss with friends -16:48 to 17:17))
T: Left or right?
Ss: Right--right (0.2) experience
T: Ok. Erm:: (0.2) Sit with your partner (0.2) your partner. (0.3) Your partner? Where's your partner?
Ss: Here
((Ss doing discussion))
T: Jana again no partner.
((Ss continue theirs discussions – 17:36 to 18:16))
T: Result will be what you have. (0.2) Ok, look at erm:: ok background, Haziq.
(0.6)
T: Ok, look at the job advertisement in front there::. The job that you are going to apply is a ↑Land: Surveyor. What you have to do is you have to respond to ↑the job advertisement. (0.2) What do you need to respond? For example if it's stated there: Certificate, Diploma or Degree. So, let's say you want ↑to: write a letter. So how are you going to respond to this advertisement? Do you actually have--have this Certificate,

Diploma or Degree? Do you have the er:: work experience there: 1-2 years? Do you able to perform shift duties? Creative and flexible? Do you have all of these things?
Ss: Yes
T: So, try to respond to this job advertisement.
((T went around to discuss with Ss))
Ss: How to write =
T: =statement? I see you have the certificate?
Ss: Yeah
T: ‘I have the Certificate in bla bla bla:. I do have the certificate, but I don’t have the work experience.’ Make a statement.
(20:00) ((T went around to discuss with Ss -19:38 to 21:20))
(   )
T: Suhaili quick! So that people can see it.
(25:00) ((Ss’ group discussion - 21:23 to 26:37))
T: You have another 5 minutes!
((Ss continued with group discussion – 26:40 to 27:53))
S: *Penilaian apa ye*?
T: Presentation
S: Training presentation
(   )
T: Any experience. At least *yang ni* work experience.
(   )
T: Work at? (0.2) And I’ve ↑got?
Ss: A’s
T: Work Ace?
Ss: Got A
T: Oh::
Ss: A--A. A for the processes and procedures
T: I thought you got ‘a’ and then blank there. And--and what?
(30:00) ((Ss continued with group discussions and T moved to another group to check on their work - 28:55 to 34:57))
(35:00) T: Ok, done? Hah? (0.3) Done?
((Ss’ group discussion continued because some Ss have not finished - 35:02 to 36:26))
T: Ok, can I have er:: the first group to present? (0.3) Anyone would like to volunteer?
(   )
T: Ok--ok listen--listen to their response towards the er: job advertisement.
Ss: Oh no! Small. Astagfirullahalazim. Tak nampaklah.
Group 1: ‘I have a Diploma in Land Survey from Ungku Omar Polytechnic. My CGPA for 6th semester is 3.73.’
Ss: Ish! Fuh::
Group 1: Er: for the:: experience, ‘I don’t have any work experience but I gained through Industrial Training for six months at Shah & Co. Consultant and I’ve got A for the evaluation.’
T: And?
G1: As:: perform and (duty list) I: will be to perform shift duties in your company (.)Because then during the Industrial Training, I feel comfortable with that.’
T: And?
G1: The criteria and character. I may not a creative but I am a responsible person because I can do the sign on paper. I--I also can work for overtime if I couldn’t finish the work (   )

T: hm: ok, do you guys (   ) Ok. Let's look up--look back to the first one again. The Certificate--the Diploma. How did you guys erm:: respond to the:: first er:: (0.2).
G1: First one?
T: Yes, the first one.
G1: 'I have a Diploma in Land Survey'
T: Ok, so: you have one point there. Ok, you have a Diploma and then the second point?
G1: 'From Ungku Omar Polytechnic.'
T: Ok:
G1: Er: 'My CGPA for this semester is 3.73.
T: Ok, you have to prove to show that you have succeed in your Diploma. Ok, next one. Next point?
G1: Er: 'But I don't--doesn't have any work experience.'
T: Ah ok, listen to this. He doesn't have any work experience, right? Stated in the job advertisement, they want those with ↑a: minimum one to ↑two=
Ss: =years
T: Years. And then, what other things that (.) he wrote back to back up?
G1: 'But I gained through Industrial Training for six months.'
T: Ok, he stated there: he has been through ↑a: *L I* for six months. And then?
G1: 'At Shah & Co. Consultant and I've got A for the evaluation.'
T: And then he stated there, he got an A for ↑the *L I*. But maybe you can include there some of the work that you have done during your *L I* so that they can have the: ok basic thing ok. This guy have done this and this and then maybe he is suitable, although he doesn't have ↑the (40:00) ↓experience. Ok, next one?
G1: Er: ' I'll be able to perform shift duties.'
T: Ok
G1: 'Because then during the Industrial Training, I feel comfortable with that.'
T: See! There is a justification that why you are able to perform shift duties. And then, the last one?
G1: Er: 'I may not a creative person and I am a responsible person.'
T: Ok: Ok, good. Thank you. Next. Can I have next er:: (0.5) Ha: Iju?
Iju: *Mak eh!*
((Group 2 was getting ready to present in front))
∅
Group 2: (   ) Thank you for this opportunity. Er: 'I have Diploma Industry from Ungku Omar Polytechnic Ipoh.'
T: Ehmm:
G2: 'I don't have any work experience. However, I have some experience during my: practical--practical training for 6 months at the (   ) Company.
T: Ok.
G2: I can perform extra shift duties (.) during my work. I also have AutoCAD software and other computer skills.
T: Ok.
G2: Er:: then er: (0.2) ((coughing)) 'I am able to do er: many work such as er: at the--at usual or the site work or office work.'
T: Then? (.) Or to show that you are creative and flexible person? Ok, maybe you can er: you put more under your er: *L I*, ok? Write down what kind of job that you have done, ok? Who would like to go er: next? (0.2)
Ss: Khairul
T: Ah: ready?
((Group 3 was getting ready to present in front))

T: Ah: ok
(   )
Group 3: 'I recently graduate from Ungku Omar Polytechnic Ipoh where I joined industry in Ipoh. I don't think I have enough experience. I have gained a new experience from my Industrial Training at (   ).'
T: Ok.
G3: 'I am a very: flexible person. I can manage my time perfectly. So I have no problem if my shift is busy. Based on my creative skills, I am sure your company will be great with my site plan produce skills.'
T: Ok. (0.2) Just the next.
(0.8)
T: er: after this choose er: Haris and the group. They are excited to present.
((Group 4 was getting ready to present in front))
(0.5)
Group 4: 'I have graduated from Ungku Omar Polytechnic Ipoh and I have certificate in Diploma Industry for the past three years. I have work experience in my six-month Industrial Training at a (Netternet) Surveyor company and also make part-time job oversea. I'll be able to perform shift duties where I can arrange my time to work in day, night, weekend or weekday.'
T: Then?
G4: 'I am creative and flexible person and I am sure I could make a immediate and valuable (45:00) contribution to your company and I am also on time.'
T: Alright (.) the next group.
((Group 5 was getting ready to present in front))
Group 5: Er: "Previously I have a Diploma in Industry from Ungku Omar Polytechnic and my CGPA is 3.48 without (   ). Er: I don't have any work experience in any land survery in any company. However, I have--I have an experience in Industrial Training about 6 months.' Then?
((Ss laughing))
G5: 'I'm able to work in the shift day or night like I do in Industiral Training. I am creative but not a playful person. I can make a perfecting plan using AutoCAD overdue in terms of the plan.
T: Ok (.) and you can put more information regarding your *L I*, ok? One more group.
Ss: Irsyad
T: Ha: Irsyad
((Group 6 was getting ready to present in front))
(   )
Group 6: "I have a Diploma in Land Survey from Ungku Omar Polytechnic.'
T: Then?
G6: 'I have an experience in Industrial Training about 6 months. I am able to perform a shift duties at a day or night. I am an active person and flexible in any place no matter where or when.'
T: Ok. You can put more like---give him a list of things that you can do whether you can include more of the: proof to show that you are able to do that and more:: information regarding your *L I*. Ok guys now er: take our your module. So basically based on your presentation, I can see that you actually know how to respond to ↑the: ↓job advertisement. Ok now, er: module page (0.2)--take out your resume. ((T clearing throat)). Module page 83. (0.6) Ok, on page 83, are the example of er: the format of a resume, ok? (0.2) On top you have ↑the? Address of home. (0.2) The centre are the receiver?

Ss: The sender
T: The sender and the follow by date. And then, the address of ↑the?=
Ss: = The receiver
T: The receiver. After that you have ↑the?
Ss: The:::
T: 'Dear Miss Karen.' (0.2) What do you call that?
Ss: Er:
T: Salutation.
SS: Salutation.
T: You have salutation there: and then you have the title of ↑your:: letter
Ss: Letter
T: And then you have the first paragraph: as stated there what do you have to write in your first paragraph?
Ss: The::: certificate (0.4)
T: The first paragraph, what do you have to write there?
Ss: Certificate. (0.2) The first paragraph
T: The ↑first paragraph.
Ss: Ah: How to--how to write the:: recent--the job
T: How do you know the information then? You want--
Ss: Position
T: Yes:: good, the position that you ↑want: to apply
Ss: Oh: position
T: Ok and the second--second paragraph would be?
Ss: Er: on my certificate
T: On ↑your qualification:
Ss: Qualification
T: Ok, and then the third paragraph?
Ss: Experience
(50:00) T: Would be ↑a=
Ss: =Experience
T: Experience: the skills that you have, ok? And then: what we request for?
Ss: Interview. Ask for::
T: The closing: of the [letter, ask ↑for: [interview. At the end there you ↑have?
Ss: [Salary [interview 'Yours sincerely'
T: Ah: 'Yours sincerely' and ↑then: sign ↑and: write your name there, ok? (0.6) Ok next, look at the advertise--sorry letter on page 83 and letter on age 85. Ok look at the different there. Er: the first letter is end with 'Yours sincerely' and second letter ends with 'Yours faithfully'. What is the difference?
Ss: Er::
(0.2)
T: Can you detect the difference? What is the difference? (0.5) Look at the letter.
((Ss looking for answers))
T: Hmm:: (0.3) What?
Ss: (   )
T: Again louder so that everybody can here you. (0.2) Ah: listen--listen to your friend here.
Ss: 'Yours faithfully' we don't know who we send the letter.
(Ss laughing) (0.2)

T: ‘Yours sincerely’?
Ss: ‘Yours sincerely’ we know er: the person that we want to send the letter.
T: Ok? Ok. What is ok? Ok, ‘Yours sincerely’ is when you know::=
Ss: =the person
T: The person. ‘Yours faithfully’: you don’t know the name of the person, ok? Now, you have your resume with you?
Ss: Yes
(0.3)
T: Ok, er: (0.4). Ok, based on your resume, ok take out a piece of paper, try to do:: the cover letter for the job that you want to apply based on your cover letter--sorry your resume here. Ok?
Ss: Now?
T: Yes, now. (53:23)

Transcription of Interviews

Recording No.: 9
**Notes:*
A: Interviewer
B: Interviewee

A: Ok, Assalamualaikum and good afternoon.
B: Waalaikumussalam warahmatullahiwabarakatuh
A: Ok, thank you for agreeing to talk to me. The purpose for my research as you already know is to investigate the use of code-switching among the English Language lecturers in the Malaysian Premier Polytechnics. Ok, I just want to have your views on your own practices and the use of code-switching in this--in the classroom. Ok, so the content will be confidential and only be used for this research purposes. Ok, I’m sure you have heard of code-switching, right?
B: Yeap
A: So, what can you understand about code-switching?
B: Er:: basically from my understanding, code-switching would be a:: now they use a:: students’ first language in order to teach them English as a sedond language maybe in order to help them er:: to understand maybe difficult words in English that we need you know to give them the meanings or maybe in transfer information because er: basically, er: er:: in teaching English we need to use you know sometimes a simple language to match with the students are able to understand them:: the information that we want to. You know erm: the information that we want them to get. Er: but sometimes would be difficult er: for them to understand so we need to use some of the code-switching in order for them to get the information.
A: I see, Did you code-switch just now in the lesson? Did you notice?
B: I think a bit there
A: You did a few
B: Yeah a few there
B: So why did you--why did you it just now?
B: Basically the first part would be:: I’m giving the definition because some of them actually when I asked the meaning and some of the students give the meaning in Malay. Maybe would be good as er:: for some students to actually get what actually the meaning of the words.

A: Ok
B: And then some jokes actually.
A: Yeah. So was it a planned or unplanned one?
B: Er::: basically usually when it comes to joke, naturally er:: be in BM in a Malay as a:: you know, it's something natural.
A: I see, so apart from code-switching, do you have any other strategies for them to understand what you have taught.
B: Erm:: Maybe give other example instead of just giving the direct meaning of the word, we can give er: you know situations so that they can get the meaning of that words.
A: Ok, your first language would be:=
B: = Malay. Second language would be:: [English.

[English.
A: So the subjects that you are teaching would be?
B: Err:::: Communicative English 1, III and the:: English for Communication for deaf and mute students.
A: I see, ok. So I'm sure for the deaf and mute students, do you do code-switching?
B: Er:: yes, a lot!
(Both laughing)
A: Alright. So how long have you been teaching in this particular subject? AE501.
B: About three years.
A: Oh…ok. Do you like teaching it?
B: Yeah
A: Ok, Ah:: do you think your students would benefit from you doing code-switching in the class:
B: Yes. I think so.
A: Do you consider yourself as a proficient in English?
B: Er:: as a Malay speaker as a first language, I think there's a lot of things to be improved
A: Ok.
B: With language you know, it nevers going to be er: perfect in language.
A: Ok. What are you strengths as a lecturer?
B: As a lecturer I would say that er: I'm able to attract students' attention in order for them to enjoy the class.
A: What about weaknesses?
B: Weakness would be er:: sometimes I am the second speaker of course sometimes we also struggling the specific words to use even though we are--are--I'm a English lecturer.
A: So do you think that you code-switch, I think you only teach for how many years? Three years?
B: Three and a half.
A: So I cannot say that you code-switch before or now. Is there any difference from the first you teach and now?
B: Er::: I think the first year er:: er:: ermn no, the earliest in teaching would be straight English, because I think that No--no--no-- code-switching is not a good thing.
A: Not acceptable
B: Yes, not acceptable here but then experience--from my experience, then I know nothing wrong with code-switching.
A: Ok. So you have to use it
B: Yes

A: Moderately. Ok, the lest question, what do you think of English in general, is it improving, standard of English
B Ern:: in polytechnic?
A: It’s up to you.
B: I think in Malaysia itself, English has become one of the prominent language other than er::: er::: Malay. And I think it’s good because as we know English is an international language. So we should, you know, at least erm: even we are not fluent in it, maybe we have to actually know the language in order to survive at least.
((Both laughing))
A: Orait. Thank you so much.
B: Ok, see you.
A: Please do not hesitate to contact me if you have anything to add or clarify. Ok, er:: any questions, you can just come and ask me.
B: Ok.
A: Thank you.
B: Welcome. (05:05)

iv) PSA Lecturer A Transcriptions

Transcription of Classroom Observations

Recording No.: 10
Date: 17/9/2014 (Tuesday)
Time: 8.00-9.00am
Class: DPB 5C
No. of Ss: 24
Level: Mixed ability
Age range: 20-22 years old
Topic: Graphs and Charts (Adjective/Adverb)

**Notes:*

T:	Lecturer
Ss:	Students
(.)	short pause
(0.2)	2sec pause
[	overlapping speech occurs
↑	rise in intonation
↓	drop in intonation

--	change of the topic/rephrase/repeat
::	elongated speech/ stretched sound
=	latched speech, a continuation of talk
( )	words spoken here were too unclear to transcribe
(( ))	transcriber's description
Ø	no talk, ambient noise
U N	capital & space for acronyms
red	Malay words
' '	Sentences read from the board/book/reading materials
**Based on Jefferson, G. (2004)*

Transcription of Classroom Observations

((T took down Ss attendance - 00:00 to 01:45))
∅
T: Ok, we will continue today er: discussion on the words or vocabularies that we used in trends, movements in graphs and charts. Try to do come practice on adverbs, please share the handouts.
((T distributing the handouts))
T: Ok, I will give you about three minutes to fill out the handouts in front of us, then we'll discuss about it, adjectives and adverbs.
(05:00) ((Ss completed the handouts while T went around to check - 03:18 to 07:59))
∅
T: Ok, we are going to see how adjectives and adverbs are used in graphs and charts. Alright? (.) So basically we want to see what is adjective and what is adverb. (0.2) What is--what is adjective and what is adverb. So, you have to understand first, adjective qualifies: the noun. For example, 'There is a two copy', as for example in the handouts, right? 'Copy' ↑is a noun, a name, right. And 'two' ↑is:=
Ss: =adjective
T: Adjective. Describing the:?=
Ss: Noun
T: Ok, anything that describe ↑the: noun, we:
Ss: Adjective
T: Called them as adjective, right? What about adverb? Adverb qualifies adjective, noun or other adverbs, ok? We are looking at this example. Like for the example, 'Please speak slowly', right. 'Slowly' is modifying the verb, right? Verb *dia* is 'speak' and ((Ss coughing)) adverb is saying how the speed is being done, ok? It's being done slowly, right? And another formula that we can identify adverbs normally, we can see the: ending of 'ly', *ye*. 'ly' is adverb, ↑right? Ok, in graphs and charts, you can see your module, ok page ((Ss coughing)) 107 (0.2) is a very good example (0.2) ok.
Ss: 1 2?
(10:00) T: 1 0 7 (0.2). Ok, if you look at the page there (0.2) add the adverbs to be used. You can see two examples there. Alright, for example, 'unemployment rose dramatically', right. 'Rose' here is ↑the=
Ss: =Adverb
T: Verb, right? It shows an ↑action--an action that is going on in the graph, ok. So, it grows. Something grows. What grows? Unemployment, ok? And what is this 'unemployment' ((T clearing throat)) how does it grow, yeah. ((Ss coughing)) It grows 'dramatically.' So ↑here: is adverbs modify the=
Ss: =Verb

T: Verb, ok and here is the verb is ‘rose’, ok. (0.2) Alright, let us see:: adjective. Ok, ‘there was a dramatic rise.’ Here, ‘rise’ is a=
Ss: =Noun
T: Noun, ok. And adjective here ↑is ↓‘dramatic’. Alright? And why it is an adjective? Because ‘dramatic’ is something that (.) explains about the ‘rise’, about the noun, ok? So adjectives qualify:: noun, right? So, describe the noun. How was the rise? It was dramatic. Ok, so ‘dramatic rise.’ Ok? So we: have looked into examples of the adverbs and adjectives. Look at your:: module. Let us see how sometimes adjectives can be adverbs, ok? Look at the words the--the--the words that are listed there. Alright, look at: ‘dramatic’. (0.2) ‘Dramatic’ is (0.2) adjective, right? There’s no ‘ly’, (.) ok? And here adverb (.) becomes an adverb, when we put the ‘ly’, so ‘dramatically’, ok? ↑‘Sharp’: ↓‘sharply’: ok? ‘Terrible’: ‘terribly’: ‘steady’: ‘steadily’: (.) ‘slight’: ‘slightly’: ‘marginal’: ‘marginally’: ((Ss coughing)) (   ), ok? The meaning are all the: same, but how you write in: graphs and charts: it matters. As I said earlier, in graphs and charts, you have to--you can use adjective, you can use adverb, ok? Variety of use in: writing, in the--in the describing graphs and charts, it will makes--it will make your explanation better: and sound interesting: for ↑the reader, ok? (0.2) Any questions?
((Ss coughing))
(0.8)
T: Let us do come simple exercise: before we go further in the graphs. (0.6). Now try to do exercise:: I mean quietly, verb:, adjective:, noun and adverb, ok? (0.3) I think you need to share
Ss: We need to share
((Ss coughing))
(15:00-35:00) ((Ss completing the handout with partner and T walking around to check on their work - 14:17 to 36:00))
∅ ((Construction work & Ss’ discussion at the background))
T: How many of you have no dictionary yet? As I already said to you earlier, in the first class::: last week. (0.3)
((Ss coughing))
T: So because of that, some of you are having some troubles to find the answers. (0.6) You don’t just pick the answers from the sky *ye*!
Ss: Yeah
T: Ok, so for--for those who don’t understand, at least if you refer to the dictionary, you’ll understand, where’s the noun, where’s the verb, adjective and adverb. These are very basic. These what you have learnt in your secondary school (0.2) ok?
(40:00) ((Ss continued completing the handout with partner and T walking around to check on their work - 36:50 to 41:35))
T: Ok class, if you all ready (0.3) er:: with the: answering the: words, adjectives and adverbs. (0.2) Alright, can we just quickly, look at the answers? (0.6) Ok, no.1?
Ss: New material
T: Alright, no.1?
Ss: New material.
T: Alright. Ok, no.2?
Ss: Identify=
T: =Identifying in=
Ss: (   )
T: (   ). Alright, no.3?
Ss: Let it
T: Let ↑it=

Ss: Rise
T: Rise, ok. No. 4?
Ss: Eco--Economic
T: Economic. No. 5?
Ss: Individually
T: No. 6?
Ss: (   ) accurate
T: No.
Ss: Accu--
T: Accusition. Accu--=
Ss: =sition
T: No. 7?
Ss: Resourceful
T: [Resourceful
Ss: [Resourceful
T: Ok, no.8?
Ss: Traditionally--traditionally. Resourceful
T: No. 8 traditional=
Ss: =ly.
T: Traditionally. No.9?
Ss: Participate!
T: Participate, no.10?
Ss: Concept
T: Concept, 11?
Ss: (   )
T: Hard to decide, alright? 12?
Ss: Equally
T: Equally, no. 13: vaccination
Ss: Vaccination
T: 14: [sustain
Ss: [sustain
T: 15:?
Ss: Emergency
T: Emergency
Ss: *Belakang belum lagi!*
T: Sorry: 16? [Specify
Ss: [Specify
T: 17: Removal
Ss: Removal
T: 18: [Ethical
Ss: [Ethical
T: 19:?
Ss: Invitation
T: Invite
Ss: Invite:
T: 20?
Ss: Evolve
T: Evolve. 21? Flexible: (.) 21 flexible. 22? Relation=
Ss: =ship
T: 23: marginal and 24:

Ss: Persuade
T: Pursue
Ss: Pursue…yeah…s u e
T: Ok, right. Er: if you don't have any question, we are going to have the replacement class on Monday and next Tuesday, right?
Ss: Yes
T: At 9 to 10? Er: both days. Alright, Hartini will let you know where is the venue.
Ss: Tomorrow?
T: No no no. Next week. Ok? So, we'll continue the discussion on these: vocabularies, right, since of you are still: confused. Ok, alright. If you have any question, you can also come and: (0.2) Alright, I will meet you later. Thank you. (45:01)

Transcription of Interviews

Recording No.: 11
Notes:
A: Interviewer
B: Interviewee

A: Ok, Assalamualaikum & good morning. Thank you for agreeing to talk to me. So, the purpose for my research are--is to investigate the use of code-switching among English Language Lecturers in the Malaysian Premier Polytechnics. Therefore, I would to have some of your views regarding the code-switching in the classroom and our talk will be very confidential and only be used for this research purposes then. Ok, let's start.
B: Alright
A: Is that alright?
B: Yeah.
A: Have you heard of code-switching? I'm sure you know what is code-switching, right?
B: Yeah, little bit.
((Both laughing))
A: So what can you understand about code-switching?
B: Er:: I think the way that we try to translate from our mother-tongue
A: Ehm:
B: To another language
A: Ehm:
B: All--all happens in the brains so how do you manage to try to change the language.
A: I see
B: That is not used to us to another language as a medium of instruction.
A: Alright.
B: Yeah
A: Ok, so did you do any code-switching in your lesson?
B: Er: not really.
A: Not really yeah
B: Because er: I'm already used to: talk to the students in English. So I feel like I didn't do any cod--
A: [Code-switching
B: [Code-switching. Maybe 2, 3 per cent.
A: Yeah.
B: Perhaps.

A: I think when you talked to them personally in discussion
B: Yeah
A: That was the only time, right.
B: Yes
A: But general teaching I also did not see any--any code-switching in the lesson, right?
B: Yes
A: Was it a planned one was an unplanned one?
B: Er: I think we can say it is unplanned
A: Ehm:
B: Right, cause I never plan that er: I wanted to do some code-switching. It--it came naturally.
A: Ehm:
B: Yes
A: Ok, some lecturers said that er: they do code-switching in order--is part of the teaching strategies. Other than code-switching, what other strategies that you can use?
B: Er: strategies in terms of?
A: Er: [in order for students to understand what you have [taught.
B: [Delivery? [Ok. Er: normally what I do is that I try the very best to explain to them in English itself.
A: Ehm:
B: Unless the student is very very weak.
A: Ehm:
B: Then I need to mention the words or certain er: vocabularies in their mother-tongue.
A: Ok
B: In order to translate
A: Ehm:
B: Into English
A: I see.
B: But then that would be the last resort.
A: Alright. Ok. Alright, so what is your first and second language?
B: My first language is er: Malay, second language is English
A: English. So what subjects are you teaching here?
B: Here I'm teaching English
A: Ehm:
B: for the Semester 5 students, Semester 1 students and also I'm teaching Degree students.
A: I ↑see.
B: Yeah.
A: Ok, how long have you been teaching here?
B: About 10 years.
A: About 10 years, that's long ((laughing))
B: So, do you feel sometimes when you use your first language, do you feel guilty of suing it in the class or:: ok?
B: Frankly speaking, I: used the first language maybe less than 5 per cent.
A: I see.
B: See if--if can I will try to avoid using the first language.
A: Ehm:
B: As I said earlier, as the students' level is very very low, then if I cannot help it, I'll try to use the first language.
A: I see.

B: But then I will avoid using it.
A: Ok. Usually you use it for the weaker students, right?
B: Yes
A: I see. So, as a lecturer, what do you think are your strengths? In general.
B: My strength, ok. Erm: I tried to make the lessons er: not too rigid following the module
A: I see
B: Or syllabus. Sometimes I slot in some jokes.
A: Ehm:
B: To get the attention from the students.
A: Ehm:
B: Because the topic is not very interesting
A: I see ((laughing))
B: So, as a lecturer, my--the challenge that I‘m facing I need to think of er: a way to attract the students as my audience
A: Ehm:
B: So that they have fun learning language but at the same time they improve on their language.
A: What about weaknesses? Do you notice any weaknesses that you have?
B: Er: perhaps if I'm carried away with the explanation
A: ((laughing))
B: Maybe I--I--I can some of students: quite lost
A: I see
B: If I teach them er: in a fast pace.
A: Ok
B: So, I can see some students lost. So I need to repeat again, by using another er: way of explanation.
A: I see. Er: do you think that you would like to improve your teaching? Any ways that you would like to improve your teaching.
B: Yeah, I think day-to-day we have to improve our teaching There's--from my understanding, there's no is perfect. Even the lecturer also is learning. As me, as a language lecturer, English Language, is not my first language. So, for me, I am also learning. Perhaps in some period of time, I'm learning new words also from my students. So I think er: is two-way communication and is a two-way process of learning. The lecturer learns from the students, the students also learn from the lecturer.
A: Ok, that's good to hear ((laughing)). Ok, the last question. What do you think of English in general? Do you think it has increased students' proficiency? Do you think it is better now compared to the previous time when you started teaching because it seems that you have 10 years?
B: Yeah
A: Of experience. So do you see any differences or just the same?
B: I think er: it got a huge differences from the first year that I was teaching here. The students er: was er: mixed of--major of weak students rather than good students. But as er: as I can see now, even the intake for this--the polytechnic, the students are quite good. They are selecting students er: who are very good in certain subjects in their SPM. So I can see, if I'm comparing first semester students er: ↑then ten years back and now, I can see the Semester 1 students are quite fluent
A: Better
B: Or better in English compared to ten years back.
A: So do you think that you do more code-switching back then compared to now?

B: Ah yeah. Ten years [back I need to
A: [Because of the students
B: Yeah because there's a lot of weak students compared to now.
A: Ok. Alright, thank you very much for talking to me. So if you have anything else to add. You can just inform me or tell me later.
B: Alright.
A: Ok, erm: anything--any questions also you can ask me: afterwards, later.
B: Ok.
A: Thank you so much. (07:28)

v) PSA Lecturer B Transcriptions

Transcription of Classroom Observations

Recording No.: 12
Date: 18/9/2014 (Wednesday)
Time: 10.00-11.00am
Class: DPB 5B
No. of Ss: 31
Level: Mixed ability
Age range: 20-22 years old
Topic: Mini Project (Progress Report)

**Notes:*

T:	Lecturer
Ss:	Students
(.)	short pause
(0.2)	2sec pause
[	overlapping speech occurs
↑	rise in intonation
↓	drop in intonation
--	change of the topic/rephrase/repeat
::	elongated speech/ stretched sound
=	latched speech, a continuation of talk
( )	words spoken here were too unclear to transcribe
(( ))	transcriber's description
Ø	no talk, ambient noise
U N	capital & space for acronyms

red Malay words
' ' Sentences read from the board/book/reading materials
**Based on Jefferson, G. (2004)*

Transcription of Classroom Observations

((T was having small talk with the students and introduces the observer))
T: I just thought that I look first. Then you need some more time, then you take your time. Ok, I'll come back to you later. And we have with us my good friend, Puan Mazlin, alright.
Ss: Hi!
T: She's gonna be in the class today. We were very good when we were together at PUO, Politeknik Ungku Omar, English lecturer and now she's doing her PhD in New Zealand, right.
Ss: Wow:
T: So she's going to be here to see how ↑behave today ah: and the she's going to report to her Professor in New Zealand.
((Ss laughing))
T: Ok, Welcome Puan Mazlin.
M: Thank you so much. Ok, so, I'll be here just to watch how: ok, you behave, right.
((All laughing))
M: Tuan Syed as well in front here. So if you don't mind me, I'll be voice recording the whole session for about one hour. So there's no video recording, so no worry about touching up. You know I'm a bit sweaty because I walked from J P A just now
T: JPAM
M: I used to walk in New Zealand, not driving so I though oh: ok let's walk. Oh my God!
T: Ok, it's good
M: It's so hot!
((Laughing))
M: Alright, so I hope you can our full cooperation for this. ↑Just act as you are, normally at in the class. Alright?
Ss: Ok
M: Thank you so much. Continue.
((Lesson starts))
T: Alright, er: we call upon the first groups just now to er: er:: don't anything else look right.
Ss: No no no
T: ((Laughing)) Anita. You take the owner ah: to be the first group just to brief. Ok, the purpose today for our gathering here is to brief us on your project development or progress. So remember the Group Projects? Buy one. So you tell us how much progress you've made before our real presentation next week, remember?
Ss: Yes
T: Ok, you just refer--are just show the ground whatever, short report you have and let's look. Ah:: how much did you completed the programme. By now, I'm hoping that you almost complete your task, right? At least you give75-90% of around that figure. Ok, so that by next week, then your chart: ok? So, nothing much. (.) Ok, so let invite: the first group er: because the first group is supposed to be that group but you guys are not ready so we need some peopler to come, right? So I go back to you later, alright? Er: I'm sure I'll go back. (.) Ok, so er: ok now, Atikah, Zaitun, Yang Puteri and another new member,

Nur Fatihah. Alright? Can you please come forward? And--and let us know. Er: This is like the pre-presentation. (0.2) Nothing much, ok. Ah: it's not like the per--fect report. Of course a perfect report is exceed in number. It's just simple. Very simple ah:: presentation. Ok? So all of you members will just participate, ok. Just rehearse. Ok, if there's anything--anything I need to correct to--to improve, then I'll give, ok. We'll see. So that we can improve on your actual report next week. Ok? Ah: that is the purpose. That is the full idea. Ok? So, that is the first group, the second group: Nur Hanisah, right?
Ss: Yes!
T: Ah:: you'll be the second. Just get ready. Next ah.
((Laughing))
T: Get ready with your friends. Ok, first this group, next yours. Ok? Just bring along your whatever: A4 paper sketch, graph whatever, chart and the outline. And the rest of the group members, they can also comment, er: they can also anything about it. Alright? Overall report and strategy.
Ss: How: (   )
T: Yeap. What is it?
Ss: How long is the presentation?
T: Oh: only two minutes--two minutes only ah:: (0.2) Two minutes yeah. Ok, the third group is I supposed is Abdul Rahman. (0.2) Yes or not? Abdul Rahman is one. Er: Edri, right? If you are ready, maybe you can go first and maybe they come. Er: ok, let them go first. Clap!
((Ss clapping))
T: Yeah: good clap--good clap to them. After this, I'll get back to you guys. Er: yeah. ↑Eh! Eh! *Apa ni? Semua--semua--semua!* All the guys up.
Ss: Oh:
T: You cannot leave your big boss alone. If he's going to die, that's it. We won't allow that, yeah. Ok? So in the actual presentation: next week, ↑all of you have to come out speak. Remember, it's not just one responsibility to do. All of you. Ok? That's your--part of your (05:00) planning. So next all of you, participate and speak individually. Otherwise, how am I going to give marks. It is not fair, ok? Alright? This one er: just-just go first.
((Ss get ready to present))
Group 1: Assalamualaikum. Good morning.
T: Attention at the back, please. Ok, continue.
G1: Er: our title for this er: presentation would be the Internet and cyber-crime--cyber-crime in Malaysia. From 24--from January until er: Disem-- until:
T: Next week?
G1: September. First of all, I wanna talk about the pie chart on our progress. The first is er: from one September--one September er: until three weeks to identify the report--identify the report. The (0.2) the second week er: er: go for analysis of data. 1 week and above. And middle of this process, we will be analysing the data. (0.3) Now we are in collecting: network. We are in the report progress, doing report. And for next week, we'll be doing a presentation. And the *apa* handover--handover date will be that progression for this presentation. (0.2) The date about the last (0.6). The type of cyber-crime in Malaysia, we have find out about 9 types of cyber-crime. Our analysis on intro--intrusion bank, intrusion your (   ) cyber, express pump, contaminated pc pop (0.3). I will talk about (0.2) of the definition and examples types related for each type-for each type.

T: What about the other gang members that want to add in a bit er: maybe. Maybe er: in actual I think you have to speak up also. Alright? Ok, I think it is a good practice for you. (0.2) You are just scared huh with ther members? (0.10) Comment to Izuan we have talked about it already. You've finished everything? Is there anything else left to-- ok, Just simply brief us just give a very brief er:: you don't have to explain now because after if you are gonna explain now, then there'll be no fun until next week. You know, Just simply think about it. Just way that we have to make the graphs, probably the pie. Ok? But don't tell us about the data let's look at it next week, remember? So, you've done qualify. Then you can revise when you completed ↑the ↓chart. If you are using bar chart whatever just 'Bar Chart 1' eh, then you--that's all--that's all. And then, done. Ok, that's it. It's not a complete: er: detail ↓presentation, ↑ok?
G1: If you can see here, er: this is the: bar chart of--of our reported incident based on general signal specifying state 2014. So, as you can see:
T: No no no no: not reveal--don't reveal it yet. I don't give permission. That is really nice. ↑Remember, if you have given out such details there will be no more fun for you, no excitement whatsoever. So you leave it there for next week. Ok, just leave it there until you have completed, ok? I think that's all. Ok, alright thank you guys as he has already done with the language one & only. ((Ss clapping)) Ah:: very simple. Not need just say it all *ye*. Ok, (10:00) alright, thank you Izuan. So ladies, are you guys ready now? Ok, er:: (0.2) Fatihah, Atiqah, Zaitun, Yang Puteri. Yes? The ladies, please come. Thank you.
((Clapping))
T: We have er:: 2/3 ladies and 1 group guy in this class, right?
Ss: Yeah
T: Still the ladies are the majority, as you can see. Ah::it's er: overwhelming. The rates are overwhelming than the guys. A big force, ok show your strength, ladies show your strength that you are even strong. Alright, can we start now?
Group 2: Assalamualaikum & a very good morning.
T: Waalakumusslam
G2: Firstly, I want to say sorry that I'm not the print out the hard copy for the schedule
T: No need to be sorry. We are not here to say sorry. ((Laughing)) Just get on, just get on.
G2: Er: actually we would like to er: make a report about 'fotal disease'
T: What? Say that again.
G2: 'Fotal' disease
T: Fatal diseases in Malaysia.
G2: Fatal disease in Malaysia.
T: '--seases'
G2: '--seases'
T: In Malaysia
G2: In Malaysia 2010 to 2013
T: 2010
G2: 2009
T: 2009, 2013. That's about five years.
G2: Yeah
T: Alright
G2: And we make a pie chart of this--this diseases. Er: it's about (0.3) ten diseases that we have conduct. Most of the diseases are like this (0.4)
T: *Nanti* they remember, they can: get--they can have a leader, but don't reveal all *ye*! Ok, there'll be a lot of diseases just about 'A': alright, kind of diseases

G2: First, that we found: like (   ), pneumonia. Second, list of preventive system. Third, er: the need of the list to prevent.
T: Yeah, good. Ehm: (0.3) Then the types of er: statistics of what is it going to be. (0.2) Ha:: and then I think you tell the class why you are interested with this topic, you know. Why you choose this topic, disease. Because this is not your--your--your area *kan*. Your area is on: building. But why choose this topic and you want to explain that in the class. (0.2) Alright, that is an objective, one of the objectives in your report. Ok? Maybe that's how you wanna start to get there.
G2: Er: Just to [see
T   [yes, good
G2: That because these people erm: many type of diseases shows that Malaysian have er:: (0.2)
((Phone rang))
T: Continue
((Ss paused while T answered his phone call))
(0.12)
T: Yeah, have you given--have you told how you design the survey?
G2:Because the erm: usually people are er:: how you live in er: further diseases. 22 per cent. We might think that there are two type [of diseases when er: pastu kita kata because of that.
T: [Ah::
((Somebody came to class and T went to see her for a while - 13:46 to 14:26))
∅
T: Ok, alright. So I think that's quite clear already. So what type of statistics that you are going to use, bar, pie chart. Alright, I think that's ok. Any question? Q&A? Q&A? Any questions? Alright. Ok, I think that's all ah?
G2: That's all.
T: Ok, thank you very much. You can go now. Thank you. You can leave. Give a clap.
((Clapping))
(15:00) T: Ok, moving on: to: the next group. Er: Abdul Rahman, right? We go back to the guys. Sorry Sorry. At the back there, erm:: (0.5). ↑Akma! Akma, right? Ok, Akma, Izuan, Chu Li. ↑Ah: ok ye.
((Group 3 got ready to present in front))
T: The three groups already present. Ok, Akma, Izuan, Chu Li, Hafizi And Izzudin. Only three of you? (0.5) They'll come tomorrow. They come the day after the class. Ok *ye*, act--act naturally as possible. They should be involved with this project as well. They cannot simply: tumpang you know. (   ) Only the three of you doing. It's not fair isn't it? Some them will push their marks. That's all. *Biarkan*. Ok, continue please. I mean you can continue the project along. (0.2) So, all along, only the three of you are working on it? Pergh:: 99 per cent it's you. Just have to ride the bus, pack the bag and: you get off the bus, you know. Ok ok *lah*. So we are moving out of time. We have *dua otai* you know from Sem 6 or whatever. So they are not around. But whatever they have we shall see now. Er: ok.
Group 3: Our study is Accidental Factor of the Road Accident in Malaysia. We: have--we have the main point of types of accident in Malaysia, with a vehicle list the accident in Selangor.
T: In Selangor or Malaysia?
G3: Malaysia. Ok, we also have a cause of accident in Malaysia. Means the accidental factor.
T: Causes for that

G3: The causes for the accident.
T: That is what you are going to explain later on, right?
G3: Yeah
T: Causes of: why accident happened. Why? I mean why: the causes of accident *ah*. You don't have to reveal it now.
G3: We also have the--how to prevent from accident.
T: Oh yes. I like that. Ok? So that is going to be also explained next week?
G3: Yes, it is.
T: Aha: that's the way--how to avoid accident, right? That's the way to avoid accident, right?
G3: Yes
T: Alright
G3: Now we confirm that…next week--next week
((Laughing))
T: Ok, why did you choose this topic perhaps you can--you can tell ((laughing)) you can tell first why you decide to choose this topic? How is it important to you, be fast. You have to tell the class.
G3: Tell the class?
T: Yeah
G3: There is the precaution: for er: please be precaution for: prevent the accident.
T: Because we are stupid. Eh! Ah: *lebih berhati-hati* yeah
((Laughing))
T: *Saman* if you say *ye*! But then again yeah we have to be careful yeah while we are on the road, right? Er: *lebih berhati-hati* on the road, because of New Zealand *punya* voice *ye*. Ok, anything else?
G3: We have er: line graph
T: Ok, you have the line graph ok, You have five. Whose line?
G3: Physical er: facilities in Malaysia. We also have pie chart
T: Yes.
G3: For the accidental factor
T: Alright
G3: We also have the--this gantt chart for us.
T: Alright? Anything else? I think you already have enough. Ok, alright good luck. Thank you so much.
((Clapping))
T: And now *lebih berhati-hati ye*!
((Laughing))
T: Alright, who's next? Er: Ashikin, Alfian, Damatri, (   ) (0.3.) Anisah. Alright. (0.5) Anisah is going to lead is it? So you decide all these *ye*, in your actual participation next week, you have to decide who is going to say why and how long. You have to divide this task among your group members. So that, the person will have to go time to speak probably. So (20:00) that you can get a fair: er: marking ye. Of course, one persons who speak most of the time can--will going to get most of the marks. It's fair, right? If they speak less, it's their business, right? That is your internal: preparation lah. That's how you plan. Ok? Alright? That's fine.
Group 4: Asslammualaikum
Ss : Waalaikumussalam
T: Waalaikummusalam warahmatullah
G4: First of all, we want to er: make a correction for our title.
T: Oh, really. What is the title now?

G4: We went: a research for flooded areas in Ampang Jaya area.
T: Oh:: only specific in Ampang Jaya area. Oh:: why--why is why the changes?
G4: The : resource from…the data
T: Oh: difficult to get data.
G4: Ah: we compare only
T: Ok, you only managed to get er: statistics only in Ampang Jaya area?
G4: Yes
T: Ok, can you tell us where you get that information from?
G4: Website
T: Website or
G4: (   ) The father (   )
T: Oh:: it happens to be your father?
G4: Yes
((Laughing))
T: Make sure it's reliable, you know. Make sure you have the statistics
G4: Yes
T: Or the data. Not only hearsay. You don't hear from what you dad says to you, right? You need to back-up data with statistics. So you get the statistics from the fire department Ampang there? Alright, thanks. Alright, good.
G4: Er: the total for:: oil and business
T: Ladies, guys: excuse me
G4: For the January until July is
T: January July this year?
G4: Yeap
T: Ah:
G4:300 and
T: Eh! You don't reveal that yet. You do it afterwards. Put it back in the thing. Just tell us briefly *la* ok you did a research in Ampang Jaya area and why. Just now you explain because your father is attached to the fire department, right. People will get it from the ministry perhaps, right. You don't need to reveal that information. Just give us er: tell us why you are interested in this topic, ok? Alright? Tell the class.
G4: We interested for this topic because our subject we have learnt about fire
T: It's related to your subject la. Ah:: This is a fire--fire study. Is it fire study? Fire=
G4: [=Protectant Resistance
T: [=Protectant Resistance ha:: Yes. It is relevant to your project. Are going to be a pilot?
((Laughing))
T: Is it relevant to you? ((laughing)). Maybe not: Ok. What else? What are the statistics? (0.2) Is it pie chart--pie chart, bar graph, whatever.
G4: I will show you: the pie chart
T: Ok, it's going to be a pie chart, ok.
G4: Pie chart ((Clearing throat)). In the pie chart--pie chart, we have in--we have found about
T:Ah ah ah: (0.2) Don't reveal yet. That's going to be in the next, ok? So, just tell us that you are going to use pie: chart ek: for your: presentation, right? Only pie chart? What else? You have anything else?
G4: Pie chart
T: Pie chart
G4: Erm: bar graph
T: Bar graph and:

G4: Line graph
T: That's a lot of ↑graphs! Ah: I think you are the lord of the graph!
((Laughing))
T: Ok. Details: next week, ok? Er:: anything else? (0.2) Do you find anything unusual from your research company *lain* any particular: location maybe something unusual place: Ampang Jaya, Petaling Street, Bukit Aman, you know. Maybe there it--it is. You want to tell us now or later.
G4: Later
T: Ah:: is it for later. Ok, alright Thank you very much. You can leave. Thank you.
((Clapping))
T: We have fire ( ). Thank you. Ok, how many more groups left here guy? 1, 2, 3, 4: what about Magfirah? (0.2) Maghfirah, Diana, Nabila. Bella and the gang ah: Today Bella (25:00) come with *baju kurung*--*kebaya ye* wah: Fantastic. Give them a clap.
((Clapping))
T: Yellow some more. Fantastic! Applause applause. Ok Bella.
G5: Can we begin Sir?
T: You can make er: ( )
G5: *Cakap lah dulu*
T: ↑Aitt!:
((Laughing))
T: *Sapa boleh cakap ah: bincang.* So this is a good practice actually for you ye. To be able to be fun and to do a practice. You know it's a good practice. Don't be afraid. Eh?
G5: Er: assalammualaikum and good morning
T: Waalaikummusalam warahmatullah
G5: Er: the er: we did a research about the: crime race--crime rate in Selangor in (.) 2013
T: Crime rate in Selangor in ↓2013
G5: In our report, we'll be present about the percentage of crime, er: the type of crime and the reason the crime happens.
T: Why it happen eh:
G5: The type that we have made a research are murder, rape, robbery and
((Laughing))
G5: The type of crime has different meaning and it will be present it next week. We use:pie chart: in our report. Next, we make a table--we make a table like a type of crime and total of crimes in one year.
T: Ok. So that's one, right. Ok, and the purpose right, if you want to talk about purpose you choose this topic to the class.
G5: We choose this topic because the activity of criminal is getting more serious and active. Now, it's getting worse. (0.2)
T: So perhaps we can make precautions la ye. That is the purpose for this report ye. We take precaution, avoid mankind, right? Ah:: Most problem--the problem is sometimes describe and did by er::: what did you call it? PASTU was it? ↑PATI --PATI.
G5: *Pendatang asing*
T: Ah:: this illegal--illegal worker. Some of the workers Some--some of the people ( )
G5: Foreigners
T: Foreigners ya. Illegal workers *juga banyak sangat*. That's the problem. Ok, is that what you gonna write in the report as well?
G5: No
Ss: Oh::: too bad.

T: Ah: too bad. It's ok. Actually you don't have to identify these: people. You can find out. Then you can refer to the er: population you know. But, you can still talk about it and the analysis. Ok, anything else? That's all right? Ok, thank you very much. Bella and company. Yes:
((Clapping))
(0.4)
T: What's the time now?
Ss: 10.54
T: 10.54. Er:: Zulfakar. Ok, Zulfakar, Umar, Irwan, Mohd Rizal. Any:: *otai?*
Goup 6: No.
T: All original members are from this class, right?
G6: Yeah
T: So blame on Fadilah yeah. All are *otais*
Ss: *Otais*
T: DPB 5B. Yes, I know you *otai*. Ok.
G6: Assalamualaikum and good morning
T: Waalaikummussalam warahmatullah
G6: er: Our group will present about type of vehicles accident in Malaysia from 2005 until 2009.
T: Ehm:
G6: From our research, er: type of vehicle accident in Malaysia is motorcar and
((Laughing))
G6: Oh!
T: You keep forgetting: this guy. I keep telling you: but I guess you are too excited to share, right. So save it for next one, Ok. Just simply roughly tell us the picture--the big picture.
G6: Our bar graph will be our secret.
((Laughing))
T: Is it? Or *tidak*: this graph is going to be the secret ha:
((Laughing))
T: Maybe--maybe when they come out: it is blank page la. It's ok. You have--we guess you create it out. If you have it now, I cannot get. Hopefully you have enough. Now, perhaps then why you decide: to choose this topic? Perhaps you tell the class.(0.4) There must be a reason.
G6: Ok, we choose this topic because the fast type of vehicle is the main transportation for we use daily
T: [Yes, alright. Aha:
G6: [Also for holiday. So this research, may use--may even ask a person about the accident that is going to face later.
T: ↑Pergh:!
Ss: ↑Pergh:!
T: ↑Pergh:! Otai otai otai.
((Laughing))
(30:00) T: Ok, good. Alright, anything else yeah? (0.2) So hopefully, this report will give us some advice ye on how to prevent accident. Ok. Alright, thank you very ↑much.
((Clapping))
T: Er: (   ) Next? Ok that group--last group er: Majid. Er: you still have the remaining people out? Ok, three or four?
Group 7: Four

T: Four. Okh: (.) cun cun. Come. Eh the--the one from that class? (0.3) Er: Anis and Natrah, they are here. Er: Arif er: Arnizan, ok Syazwan, so Khairul Ridzuan here? Ah: Without them, you'll be one person. This is very simple, ok, please.
G7: Good morning er: our group er: will present er: the statistics of road accident in Selangor er: we did time er: in year 2013
T: 2 thousand?=
G7: =thirteen. One year
T: 12 months.
G7: Er: by just er:
T: Ok Nabil, I think it's good if you have er: better icon at the back, not your body ye. Alright. You always pick your nose all the time. Maybe you know, more eye contact with the audience as well. (0.3) Yes? Yeah? Any problems? Questions after this. Ha:
G7: It is because the number of accident er: in Selangor is maybe increasing and er: that means er: out of range
((Laughing))
T: Tell me, might was er: anybody here involve in road accident from members of this class? Lady or the previous few months?
Ss: Adam
T: Adam? Really?
Ss: Yeah
T: Motorbike is it? Wah: how was it? Not so bad lah otherwise you are not going to be around, isn't it? How long was that--how long ago? How long ago?
Adam: Hah?
T: How long ago?
Adam: A month
T: A month:
Adam: A month
T: A month ago? Oh: you survived. Alhamdullillah
((Laughing))
T: So, this is why you want to educate more of us ha: how to er:=
Ss: =Get awareness
T: Get awareness from the road and prevent accident again. The same topic by just now also ye. Alright, so: alright? Ok, I think that's all. Ah!: Question? I thought you want to ask question, no? Ok, can I have your phone no.? ((Laughing)) Ok guys, thank you. Give a big clap. I think that's all er: I think that's all ye. I think all presented already. I think that's all for today. Er: Ok, so I think you are on the right course to--to do er: your presentation next week. I think you are better off now eh. I think you are: even ready, right? I think today's presentation is good, right? So what happened to my previous classes, students just simply go straight ahead with the presentation. So, this is good because it helps you marks (   ). Alright so, it boasts your confidence--build up your confidence, er: you know polish up and you can foresee what is going to happen in the actual presentation next week. Alright? So if there's any error or need improvement, and you can practice more on that to improve, ok? And one of the--advice my students all the time, if you need to practice, you can practice among your group members, sometimes you can practice in front of the mirror. You know, you talk to the mirror. You know. Look in the mirror, lean over and you can see. And that's how you can ↓improve. ↑Ok, so prac--remember practice makes=
Ss: =perfect.
T: Ok. Alright, any question?
Ss: No.

T: So, er: I postpone the test Next week ah:
Ss: Presentation
T: Oh: next week presentation. Ah: next week Thursday's single period, perhaps. So the two periods on Tuesday, we present. So, Tuesday is going to be your presentation. I don't think you can finish all the presentations in 2 hours and depends *la*. Er: 101, 101 they have the LCD? 101? This one is only for during ((laughing)). It's not working. That's why Puan--Cik (35:00) Zalijah asked for change of class. Is this what for? Is to keep in the museum already. Premier premier! ((laughing)). Ok ye. So I think 101--001 we have LCD. So we can use it--use the LCD and present there. Make sure you bring your own laptop. Maybe one person bring the laptop and the rest of you use the same laptop. No need to bring all your laptop ye. Ok, to make it even better , you can do this before presentation: so all your L--er:: power points in one: laptop, alright? So all groups from: your programme is there, before we start the class, right? So during the presentation, it's already there. All you have to do is just click that file or folder, *kan* and nothing else. So you save a lot of time. If you just want er: going to: transfer the data: or file, it's a waste of time. Alright? So you do that before class. Is that clear guys?
Ss: Yes:
T: so, group--leader of this group, make sure that: you make arrangement with Izuan. Izuan, did you have--did you bring your laptop? Alright, so make sure laptop has all the anti-virus and all. Otherwise, it'll get infected. Alright? Ok, erm: (0.4) Alright, I think. (36:35)

Transcription of Interviews

Recording No.: 13
**Notes:*
A: Interviewer
B: Interviewee

A: Ok, assalammualaikum and good afternoon
B: Waalaikummusalam warahmatullah
A: Ok, thank you for agreeing to talk to me. The purpose of my research as you know is to investigate the use of code-switching among the English Language lecturers in Malaysian Premier Polytechnics. Therefore, I would like to know some of your practices and your view on code-switching. So, it would be very confidential and to be used for my research only. Ok?
B: Yeah
A: So, what can you understand about code-switching?
B: Ok, code-switching is like for--we are native--Bahasa is our native and English is our non-native language, right? So we are speaker--second speaker--second language speaker of English.
A: Ok
B: So what--what I understand is that we switch from our native to English from time to time. Meaning we speak er: Bahasa and we change to English. That is code-swtiching *lah*.
A: Ok, did you notice that you code-switch in the class just now?
B: Er:: sometimes yes but today I think is very--I keep it to mini--minimum, right.
A: Minimum, ok.
B: So, I--because I since you know that this is going on
A: Ehm:

B: so but sometimes yes and sometimes it gets up ye you can still find a few words that I do code-switching but er: today I think is very minimal.
A: Why--why do you think you do it?
B: Ok sometimes is er: is--is natural in you, right.
A: Ehm:
B: Because you--you're sometimes er: er: confident in your Bahasa you know, sometimes er: you think that sometimes you need to stress something and you--of the understanding that students will know what you are saying to them er: so some--that-that is what happened. You know, ah:
A: Ehm:
B: So that when you say something, students really understand what you are talking about. You know, explain further, then you see--the you see students face--the look er: you know, er: they don't understand. That is where I think I need to do further explaining in Bahasa. That's where the code-switching comes in.
A: I see. So, is it a planned or an unplanned one?
B: It's a--usually it's not planned, you know and sometimes it pops out like, you know. So you know the situations--depends on the situations.
A: Apart from code-switching, is there any other strategy that you can use: in teaching?
B: Er: ok. The issue here is to make things clearer
A: Yeah
B: How to make it--how to--how to make students understand=
A: Understand, yeah true.
B: =what you are talking about, what you are trying to tell them. Er: Usually that is the only thing lah, code-switching ah: when--when you really need to do it. Apart from that I don't think there is no other way to
A: ((Laughing))
B: you know, to code-switching
A: So, that is the easiest one.
B: Yes yes
A: So what would be your first and second languages?
B: Er: English
A: Ok
B: English
A: Is your second language?
B: Ehm:
A: Ok. So how long have you been teaching?
B: Ehm:
A: in English?
B: 25 years
A: Wow!
((Laughing))
B: It's quarter of a century already, you know.
A: Ok.
B: That's the only thing in my life to do ((Laughing))
A: Do you have any guilt if you don't use English all the time: in the class?
B: Er: I think not the matter of guilt or whatever, is like er: you trying to educate your students, right?
A: Ehm:

B: You do anything to--to make sure that they--they get what you are telling them. So it's more like er: er: making your lesson effective, alright. So, that's why sometimes you need to--to do that.
A: I see.
B: Out of the conscience that er: you need to explain further so that they really understand. And I remember back in Kelantan, many years back when I was teaching there, when use English like most of the time, is like you are talking to the wall. You talk to the wall, the wall comes back to you.
A: I see.
B: So then I--I come thinking what's the point if I use English er: 100 per cent in the class and you know that your students didn't get at all what you are saying about. So, I think you need to blend that situation, I think I need to blend, you know code-switching, when to switch and when not to switch, alright? Because otherwise if you stick to English=
A: They wouldn't
B: =They--they get zero.
A: I see.
B: That is what I think. That's my experience.
A: Do you think that you are proficient in English?
B: I think I am ((Laughing))
A: Ok. What are your strength as a lecturer in general?
B: I think er: I'm able to create enthusiasm among my students so that they become positive in English Language learning er: so I can push them so that they become eager to learn about the language. I tried to avoid constraint like English is difficult, English is a foreign language, you know, it's very difficult to learn. So I--that's what I'm trying to do. I create the enthusiasm first so that when next--all of them then, they become eager to learn the language. Ok, so that is what I do most of the time. And I think I'm very good at that area. ((Laughing))
A: What do you think about weaknesses? Do you have any weaknesses then?
B: Er: sometimes is not about class organisation, class control . Sometimes to let my students speak their mind.
A: Ok
B: So I allow them to interact in the class, participate because I love to do that all the time.
A: Ok
B: And then sometimes the class get a little bit noisy,
A: I see
B: a bit rowdy but that's ok to me. That is class to me.
A: In general, English in general do you see any improvement from the day that you start teaching until now. Do you do code-switching: days before than compared to now. The level--I mean the proficiency level of the students have increased or:?
B: Is--is hard to measure, you know
A: Ehm:
B: But if we keep getting the same level of students at polytechnic level, you know. They are--I must say that their competency is--is somewhere low
A: Ok
B: So year after year, you get to see same level of competency of low level of the competency level of the students .
A: Ehm:
B: You know, I don't see any [not much er:: improvement

A: [what about the latest--the latest batch
B: But I get this latest batch, I can see some improvement.
A: Ok.
B: Because I believe English level of err is higher, right? So, that's why I can sense that this lately of these few semesters back. But the earlier one er: is all the same, you know.
A: Yeah
B: So that's why we have to push them hard. To--to love the learning English. That's what we are doing.
A: Ok, thank you so much. That's all for now
B: Ok. Thank you for inviting me. It's my pleasure.
A: If you have any questions or anything to add, you can contact me later.
B: Oh yeah, I will. Thank you.
A: Thank you. (06:49)

vi) PIS Lecturer A Transcriptions

Transcription of Classroom Observations

Recording No.: 16
Date: 21/9/2014 (Sunday)
Time: 9.00-10.00.am
Class: DJK 5C
No. of Ss: 15
Level: Mixed ability
Age range: 20-22 years old
Topic: Job Hunting Mechanics (Cover Letter)

Notes:

T:	Lecturer
Ss:	Students
(.)	short pause
(0.2)	2sec pause
[	overlapping speech occurs
↑	rise in intonation
↓	drop in intonation
--	change of the topic/rephrase/repeat
::	elongated speech/ stretched sound
=	latched speech, a continuation of talk
( )	words spoken here were too unclear to transcribe
(( ))	transcriber's description
Ø	no talk, ambient noise
U N	capital & space for acronyms
red	Malay words
' '	Sentences read from the board/book/reading materials

**Based on Jefferson, G. (2004)*

Transcription of Classroom Observations

((T was setting up the LCD and getting ready for the lesson and take down Ss' attendance - 00:00 to 02:00))

T: Where is Sakinah?
Ss: *Belum sampai lagi*
(0.6)
T: Ok, while Amir help me to set up the socket ok, let's what we are going to do today. (0.2) Last week, I probably--I asked you guys to write me a cover letter, right?
Ss: Yeah
T: And some of you didn't give me on Friday.
Ss: Yes
T: Yes. Why not?
Ss: Friday? Today is Thursday.
T: Oh sorry, Thursday.
Ss: Holiday
T: So have you done your work?

Ss: Yes, of course
T: Do you have it: with you now?
Ss: Yes
T: Ok. Submit your letters after we've finished the lesson. First that er: you guys going to look at (0.2) job ↑interviews: skills on how to handling the answers. It's locked.
(0.7)
Ss: Go to Engineering Visual
T: Imaginary Visual?
Ss: Engineering Visual.
(0.5)
T: Any socket at the back?
Ss: *Ada ada*
T: Ye ah:
((Switching on the LCD and adjusting the power point display))
∅
T: Sorry, I excuse people.
Ss: Yes mam
(05:00) ((T went out to find a socket for the LCD - 3:50 to 5:10))
T: Bring me the socket just now. (0.6) Not there the Engineering Visual
Ss: Engineering Visual students
((T tried to set up the LCD again - 05:26 to 07:52))
T: Ok ok
Ss: *Kena amik view side ni*
((Laughing softly))
T: Sorry for all the trouble. Ok, we'll start the presentation.
(0.6)
T: Ok, boys and girls. So we are looking at: prepare for job interviews. You can also use your coursework: from page: hundred and seventeen. (0.2) We already make our resume and cover letter, right?
Ss: Yeah
T: So next up is how you are facing the interviews.
(0.20)
T: This is going to be a very long to go. ( ).
((T adjusting the screen view))
T: How to make it straight? (0.8) Yes! Still late Sakinah.
Sakinah: I'm sorry Miss. Not feeling very well.
(10:00) ((T continued adjusting the screen view))
T: Ok, while waiting for the computer: restarting on its own at the moment. All the technical difficulties: today. Ok, never mind. So, let's focus on the lecture. Yesterday we are looking on how to face the job interview. Ok, so the first thing that you have to be aware of is the documents that you should bring for the job in the description. So, last two weeks we have been visited with friends. Can you just tell me what are the documents that we should bring for the job interview?
Ss: Cover letter
T: So the cover letter is also enclosed together with your resume. Send in your resume, right? So, it's not just get two things. Previously I also reminded you to bring something else for your mock job interview.
Ss: Certificate
T: Yes, your certificate. And that certificate includes your activities ↑from:?=
Ss: = School

T: Yeah, school years and ↑also: polytechnic
Ss: Polytechnic.
T: Your certificate *dan segala ni*
Ss: Er:
Ss: Oh! The transcript
T: Yeah the. transcript, your exam results.
Ss: Oh:
T: Not just in polytechnic but ↑also:?=
Ss: In school
T: Ah yeah, the one that I need it, is called SPM Certificate, right? And ↑then:: what are you telling--reminds us of tell me this *L I*--*L I* one.
Ss: *L I*
T: Industrial Training, if you have ↑any:=
Ss: =Experience
T: Letter, right? So, you need that as well. So, not just yourself, all the documents should be prepared, ok? That is no. 1, the documents. So now, we are looking ↑at: the other things, other than the documents, ok? Er: we also have to prepare: yourself. From top to the bottom. Ok? Prepare yourself on how you actually dress. For example, today most of you are wearing a training shoes. Yeah, how are you supposed to use--what kind of shoes are you supposed to ↓use to attend an interview?
Ss: Flat, leather--leather black shoes.
T: Leather black shoes. What about the girls?
Ss: Clean shoes, Prada. ((laughing))
T: But then you guys are under engineering, right?
Ss: Yeah
T: So?
Ss: Safety boots
T: Safety boots?
Ss: Manly ((laughing)). Safety first.
T: What makes a different? Wearing a training shoes, leather shoes or a safety: boots?
Ss: The safety
T: Can the girls wear high heels?
Ss: No no!
T: Why not?
Ss: Because they might even fall….it's not strong
T: Because it's not suitable ↑in: the work Technician, right? Because you guys are under: engineering field and you work in the factory. So you need something that ↑is:=
Ss: =Flat
T: Flat. So that you guys can run around, ok? Other than that, not just shoes. The shoes come together ↑with?=
Ss: =Socks
T: Socks. How many of you wearing socks?
((Ss raised their hands))
T: You have not wearing socks because it's ↑a: casual shoes, right?
Ss: Yes
T: But when you wear safety boots, do you wear socks as well?
Ss: No: no
T: So for your interview, I would like to see=
Ss: *Tiru McDonald*

T: Not just leather shoes, er: it comes together ↑with: socks and please: try to wear: one coloured shoes because one coloured shoes shows ↑your=
Ss: =Formal
T: Yes, you are in the formal condition, ok? Sometimes your: shoes come with variety colours of erm:
Ss: *Tali*
T: What is it called?
Ss: *Macam*: rope--rope
T: Rope? *Tali kasut tu*?
Ss: Shoes--shoes lace
T: Ah: Shoes lace. Perhaps it comes: with lots of colours, right?
Ss: Shoes lace *tali kasut*
(15:00) T: So try to make it one-coloured shoes together with the shoes lace. (0.3) If you wear something white, it should ↑be:=
Ss: =Green
((Laughing))
T: Yes (.) Ok, so we are looking from the bottom, the shoes. Before moving up. So the girls? (0.2) Are they supposed to wear pants or *baju kurung*?
Ss: Pants, *baju kurung*--depends on situation
T: Depends on the situation, ok good point. What are the situations?
Ss: For the design--if the interview: may also have constructions
T: Ehm: than, they better wear?=
Ss: =better wear: safetyness
((Laughing))
T: They better wear--are they going to wear--they better wear: pants
Ss: Pants
T: Right? Because showing that you are prepared to do ↑the=
Ss: =Job
T: Yeah, the on-site job. If you wear *baju kurung*, then you are supposed to attend what kind of interview?
Ss: Interview of: secretary
T: Maybe, if you are er: ok, if you attend an interview for OSHA Officer--Security Officer, baju kurung or:?
Ss: *Baju kurung*
T: *Baju kurung*?
S: Interview for the bank. Bank--bank--bank. Safety
T: Are you going on-site or just in front of the computer doing report?
Ss: On-site--on-site.
T: So you better wear?=
Ss: =Pants
T: Any ek--working area or field around engineering that you think you should just wear: baju kurung? What was yours last time: er: during your industrial training?
Ss: A teacher
((Laughing))
T: Shakirah? What as yours last time?
Shakirah: What was mine last time. I wore pants because I go around the factory.
T: The two of you?
((Ss laughing))
T: Actually boys, there’s no issues, right?
Ss: Yeah

T: Yeah, the issues only comes when it--when they are because--they are-- No. 1 you want impress the interviewers for wearing something nice. But then, in engineering field, the nice term is totally different, right?
Ss: Yeah
T: What important is?
Ss: (   )
T: Er: what I want to see is wearing pants, but the ↑girls: for the mock job interview I think, *baju kurung* is suitable enough, ok? ↑Then: let's move up a little bit. Short sleeve or long sleeve?
Ss: Long--long
T: What makes the difference?
Ss: Short is for casual
T: Yes, so formally you should ↑wear?=
Ss: =Long sleeve
T: Then, it should come together ↑with?=
Ss: =Tie--tie
T: Ok. Can it be colourful?
Ss: No
T: Can you see what colour Hisham's is?
Ss: Green--blue
T: There you tell me ha:. Are you wearing it to come and: wearing your shirt? Yeah, it's long sleeve but what did he do?
Ss: He fold it
T: He folded it up, right?
Ss: Yeah
T: Yeah. Ok, you should wear your shirts together with tie, ok. And then, the girls they have another thing also which is?
Ss: *Baju tradisional*
T: No. Their (0.2) Hijab. Yeah, their scarfs. So, how you should you actually wear your scarfs?
Male students: Please answer
T: Sometimes people have it downwards yeah
Ss: Yeah
T: Some people have it=
Ss: *Lilit lilit*.((laughing)) Spin spin
T: So the most important in your is safety. So what is the safe way to wear it ↓around your scarfs?
Ss: *Semua ada*
T: Doesn't have a lot ↑of: accessories. That is point no.1. Talking about accessories, let's look at your hand Azura. Do you have anything accessories in your hand?
Ss: Blink blink ((laughing))
T: Do you think it is suitable to wear any?
Ss: No no. What if she engaged?
T: That is another usage. Ok, you can wear it but to be sure of who using them that you are particular about security, try to wear less: accessories, ok? Especially=
Ss: =Bangle
(20:00) T: Yeah, bangle, bracelet, watches so which we are the fortunate one, so dozen are not having so fortunate. But, not very straight of: (0.2) Ok. Moving on to the last slide is ↑your=
Ss: Hair

T: Hair. ((Laughing)). Ok, as an engineer or a technician, you shouldn't have a very ↑long=
Ss: ==Hair
T: So here where, need and tidy hair, ok? So we have to look at it from the bottom to the top. Is there any questions regarding this?
Ss: No
T: Let me check (0.8) Any of you have any experience attending interview before?
Ss: Nordin Nordin.
T: What was it for, Nordin?
Nordin: Aeon
T: Aeon? Cashier?
Nordin: Yeah
T: Did you get it then?
Nordin: (   ) Kantoi
(0.3)
T: No one else? You don't have to attend any: short interview for your Industrial Training before?
Ss: Yes
T: What are the questions about?
Ss: Ask about Math, he asks about the time, he asks about everything.
T: ↓Why does he
Ss: Usually he asks about time and extra time
T: Whether you can work extra hours or not?
Ss: Yes. And did you can come early or not, emergency call, or (   )
Another Ss: What did you respond?
Ss: I (   ) Hafizuddin here is:
T: Hafizuddin?
Ss: Attend--attend interview once
T: For?
Ss: Mobile Pacific
((T checking on the laptop since it was off suddenly - 22:02 to 23:17))
T: You know what's the problem just now? Because it's on output.
((Laughing))
T: Who access this thing for me just now?
((Laughing))
Ss: Hafizuddin:
T: Ok::
Ss: Yes
T: Now we have the video. Ok, yes. So, just now we have finished on dresses for interview, right?
Ss: Yeah
T: Ok, we discussed on how you should use: which dresses to use.
(0.5)
T: What about skirts? Can you girls wear skirts, Ezra?
Ss: No, I think no. Your mom is going:
T: I know you don't want to look at them wearing skirts, but some of them would like to wear skirts (.) to impress: their bosses.
Ss: [Yes
T: [So, what about wearing skirts? (0.3) It might not be s--suiting to your image. You rather be wearing pants? Ok ((Laughing)). Ok, so let's move on. Next step is regarding

grooming, ok? And: ok the first word--the first impression starts with proper grooming. So, I would like to ask again, what is impression?
Ss: Impression
T: Yes, impression.
Ss: Opinion
T: First opinion? Someone's opinion towards you when you'll be there at the first time, ok? So, what is your impression looking at me at the first time?
(25:00) Ss: You're very good
((Laughing))
T: Ok, thank you Mahegendran. Ok, let's move on to the step which is grooming. What is grooming?
Ss*: Cantik*
T: *Cantik? Lagi* louder. Yes, grooming.
Ss: Grooming *tu yang:*
T: You groom yourself
Ss: Oh: *bangga diri*
T: Ha: *bangga? Bangga* will be boast. Yes, what is the word just now?
Ss: *Hias*
T: Ha: *hias*. So, you prove yourself. Ok, so: the grooming aspect comes under is a--no.1 there is written there for you. Hairstyles. I've mentioned about this before--just now. And then avoid wearing colours or design. What does it means by wearing colours or design? Can you wear red when you enter or come for a job interview?
Ss: Red what?
T: Red--red shirt. Red shoes. Red scarf.
Ss: Fuh:
T: So what are your favourite colours?
Ss: White--white
T: White. Other than white?
Ss: Black
T: Can you wear black? What does black mean?
Ss: Bold
T: Bold, ok. Other than that?
Ss: Smart
T: Smart. What if you have a dandruff problem?
Ss: Don't wear that shirt
T: Then you shouldn't wear that shirt, right? Ok, why--other than that?
Ss: Yellow--duck!
((Laughing))
T: Or we should call it as: pastel colour, right? Pastel colour. Pastel
Ss: What is that?
T: They are creamy in colour. Er: blue, light blue colour.
Ss: Sky blue
T: Yeah, sky blue. What else?
Ss: ( )
T: ( ) is a song. Ok, and then green--light green. And then peach are also pastel colour.
Ss: Maroon--maroon
T: Maroon? Erm: are they red? Maroon colours would be: fluorescent colours. One of fluorescent colours.
Ss: Highlight--highlight range

T: Yeah, you are right then.
Ss: (   )
T: Ok, why do we have to choose pastel colour? Why not wearing glaring or red?
Ss: Is to atrract
T: If you wear red, is it means you want to attract? It causes attention, right?
Ss: Yeah
T: So itsn't it good?
Ss: No.
T: Why not?
Ss: Maybe is: silap colour
((Laughing))
T: Oh: it means that the interviewer forgets to ask you regarding that cause there are no (   )
Ss: If you: wearing red, it makes baby easier.
T: So next one is less jewellery. Ok. Like I said just now, not jewellery, it's accessory as well. Ok, a pair of polish--polished shoes (0.2) and female--female in you? Quite yummy? Er:: are they unique? Ok, sometimes people has this problem (0.2) which is they are: sweating or: so it makes you
Ss: body odour
T: Have what?
Ss: body odour, adrenalin
T: Lots of sweating has? So you have some smells, right?
Ss: Oh::
T: So, how can you overcome that? You may ↑wear:=
Ss: =Eyebrow ((Laughing))
T: Perfume. But, the problem is sometimes people wear too ↑much:=
Ss: =Perfume
T: In the field of engineering--technician, how you should smell?
Ss: Stinky ((laughing)) but *wangi.* Smell the carpet *la*
T: Should wear like er: a very: ok. Sometimes perfume have come with lots of er:
Ss: Alcohol
T: Fragrant like. Er: fruity
Ss: Lime
T: And then, potpourri, yeah
Ss: 99, lemon, (   )
T: As long as it's not ↑too:=
Ss: =Much
T: Strong.
Ss: Durian? ((laughing))
T: Durian? Is there any perfume? Durian flavours? Ok, can you read somewhere here?
Ss: Yes
T: ↑Use:=
Ss: =A little perfume.
(30:00) T: Er: and (.) you must also come: with a bright face--with a happy face
Ss: Oh:
T: And when are talking about face
Ss: About face?
T: The girls, they usually have ↑to: put some of:=
Ss: =Lipstick
T: Yeah, what do we call this is?

Ss: Make-up
T: Yeah, make-up.
Ss: Like blusher, make-up
T:So when: should you wear any make-up then?
Ss: No wear
T: Yes
Ss: No, usually no make-up
T: So, can you just say, engineering. So engineering don't use any make-up.
Ss: Yes
T: But then, wearing make-up is a key for a good first:=
Ss: =Impression
T: Impression, isn't it? When you go out with your boyfriend, I'm sure you put on some lipsticks, right?
Ss: Not enough ((laughing))
T: So, a very light make-up maybe needed for some of you. Especially the girls, but not to the boys. I mean you can put some: yeah, powder and such. But-but not a very strong one because still, it's regarding your field, right?
Ss: Yeah
T: It is engineering. Ok, what about students who study erm: Hotel and Management?
Ss: Make-up
T: Do you have friends in--under Hotel and Management Diploma, right?
Ss: Yes
T: You have friends under Fashion Design, right?
Ss: No--Yes
T: How do you--how do they should go for their interview?
Ss: Well-dress
T: Yeah, well-dress, right. You cannot just be wearing *baju kurung*, right?
Ss: Dia kena glamer sikit--not fashion
T: Yeah, not fashionable enough. In fact, they have to wear er: very bright colours to show what?
Ss: Bright--to attract people.
T: Yeah, to show them--reflect their fashion statement. What is your fashion statement (.) as a Technician? Wearing workshop, wearing overall.
Ss: Simple. Safety boots.
T: Safety boots. So your life is not fully comfort. How do you make it comfort?
(   ) ((laughing))
T: That's all falls under grooming, ok. Now, we are moving on to frequently asked questions. Ok. (0.5) So, if you are still remember last time I asked you to write me a short composition about yourself.
Ss: Yeah
T: Composition about yourself. How you increase strength and few weaknesses, right?
Ss: Yes
T: And that come under question no.1 which is=
Ss: =Talking about yourself.
T: I have mentioned this a few times, any er: interviews conducted are very--the first questions will always be talking about=
Ss: =Yourself
T: Yourself. So, you guys have already know how to answer it, right? Which is by talking about your strength and your=
Ss: =Weaknesses

T: Weaknesses. And talking about strengths, eh sorry, weaknesses, you even make it into ↑something: positive. Ok, so are you--here are another nine questions which is no.1 ‘why should we hire you?’ Ok, why do you need to work for us. So here again, I need you to answer it by telling them what you have gained so far or what are the skills that you have: to give. You find out the skills further. One job (0.5) Programming Technician, right? So what are the important skills come under this?
Ss: Software
T: Software. What software?
Ss: Software Tools, C++
T: C++, ok. Other than that?
Ss: AutoCAD
T: AutoCAD
Ss: Adobe flash, Mathlab
T: Mathlab
Ss: Cotier
T: Cotier
Ss: Velfire
T: Velfire
Ss: Beta
T: P C P I?
Ss: P C V, P C V Wizard
T: Louder again
Ss: P C V Wizard
T: P C V
Ss: Wizard--W I Z A R D
T: W I ((T writes on the board))
Ss: Z A R D
T: Z E R
Ss: Wizard--wizard
(35:00) T: Oh: Wizard
Ss: Yeah
T: Ok, P C V Wizard. So all these skills or what you learnt will become a good Programming=
Ss: [=Technician
T: [=Technician. So that is what you should answer. Under why we should hire you. Erm: if you are: (0.2) applying for OSHA Officer, so what is important under that: job requirement?
Ss: Green card
T: What you mean by--other than that?
Ss: Er: Security Certificate
T: Security or OSHA Certificate, ok. Erm:: (02.) Tell me what is the job advertisement that you are applying for?
Ss: (   )
T: Louder
Ss: Electronic Engineering
T: Elctronic
Ss: Engineering
T: Engineering. So, still needs all these skills, right? Other than that? (0.3) Other than that? (0.3) Amalina? Do you remember the job advertisement? The one--the one that you are applying.

Ss: Er:
T: Zaini, what's yours?
Ss: Electrical Technician
T: Electrical Technician. So more on maintenance, right? So you have to repair machine.
Ss: ↑Machine, wiring
T: Wiring. So, it's more on ( ). Any other skills? Wiring.
Ss: ( ) Willing to work long hours.
T: Position of?
Ss: Technician
T: Technician Engineering position. So wiring. (0.4) So you have to mention about that as well. Ok, clear?
Ss: Yes
T: The first one you talk about your strengths, second one you talk about what you have learnt so far, including the skills. Ok, now moving on to the next point. "Why do you want to work in this industry?" Yeah, why do you want to be:
Ss: ( ) because there's no option ((laughing)), experience
T: You want to get a new experience?
Ss: Gain experience, gain money
T: Ah:
((Laughing))
T: Very straight forward answer. What do you need money? Can you answer it that way?
Ss: No--no--yes
T: Yes?
Ss: You wanna show that you're a straight forward guy.
((Laughing))
T: Give just a few words, answer it that way or answer it--this way
Ss: Because people obviously work for money
T: Ok, can be honest. Actually when you are coming for an interview, no.1 you want to be: honest. Ok? So if you cannot promise them things that you're not: going to do for example, 'I promise I would come to work on Sunday'. If you ask me. Ha: in real life, will you come on Sunday?
Ss: No--no unless
T: So you have to ↑be: honest about that. No.1 just now, being honest saying that you want to make money. Any problem with that?
Ss: No
T: No, right? But, to make people like you, enough to have you: work there? Yeh, answer it professionally by saying that you would like to=
Ss: =Gain money, earn money
T: Yeah, earn money as well as learn a few new things and have=
Ss: new experience
T: Yeah, new experience. Ok? (0.20) And how do you think you can contribute to the company's productivity? This is more of talking about your skills as well, ok? And if you have er: join any innovation competition before. You did, right? In the Semester One?
Ss: Yeah
T: Maybe you can mention about that, ok? Er: Strengths again is regarding the three things, ok, whether you are patient, honest, responsible enough, ok. 'Do you work under pressure?' What does that mean?
Ss: ( )

(40:00) T: For example just now, when I wanna to start the class, I have some problems with=
Ss: =Technical
T: Yeah, technical problems. So, is it under pressure or no pressure?
Ss: Under pressure
T: Because I want to get it start working, right?
Ss: Yes
T: Ok, ↑so: ha?
Ss: But with patient
T: Ha: but with patient. So I waited until its working, right?
((Laughing))
T: Alright, so: will you be able to work under the pressure or not?
Ss: Yeap, Yes
T: For example maybe (0.3) you are: late to work yourself, traffic jam
Ss: Accident
T: Yeah, accident and then your boss keep on calling you 'are you coming?' 'Are you coming?' 'Are you coming?' Is it under pressure or no pressure?
Ss: Under pressure
T: So will still be able to come to work and start working like a normal person?
Ss: No
T: Or you may start throwing things?
((Laughing))
Ss: No, not really.
T: Any experience before: during your practical training? Aaron, when you are operating the tramp, right, any pressure?
Ss: Er: yeah, sometimes they asked us to stop the modification but then: it is--the engineers will be as I always see in that area. So, we have to like refresh the wrong application. So the tramp has gone malfunction.
T: So?
Ss: They have to reset it back because they have to close all, and this one have to come back
T: Any misunderstanding happened during that time?
Ss: Ah yeah, some misunderstanding, at first. But however, they informed me in meeting to accept it as responsibility.
T: What about your (   ) found only by (   )? Ha: Syukri, any questions?
Shukri: Yes, erm: the pressure is when er: the model is finished and packed. But there are some things that are already worn, so we must troubleshoot back.
T: So, (   ) So, what's the pressure: to make it work?
Ss: If --if there are 100, so 100 must be:=
Another Ss: =perfect
Ss: Ah:
Another Ss: *Wah, dahsyat la.*
T: So meaning that you cannot have any reject item?
Ss: That is, er: it can be work but some mistakes cannot be function. (   )
T: So, when it's perfect, it can work but after that it cannot work. So you have to be alert. So don't have to counted as reject item. Oh: So you have to start again?
Ss: Start again
T: How many in one day. How many that you have to troubleshoot?
Ss: One thousand, one hundred

T: One thousand five hundred. It must be troubleshoot on that day itself? You cannot go back because it's not done?
(   )
T: Seriously? I need a small speaker. So, are they patient enough?
((Laughing))
T: Syukri is asking for some favours there. Are you doing it alone there? Any Supervisor together? So Supervisor has to standby witness ye. Ok, so that is one kind of pressure. Sometimes it takes your time, right? Five hundred and you have to finish it on that day, when you are actually supposed to be at home.
Ss: Overtime
(44:07) T: Yeah. Ok, so next two questions. Er: 'where do you see yourself in five years time?'--'where do you see yourself in five years time?'
Ss: Project Engineer
T: Yes! how do you answer it again, Nurudin?
((Laughing))
T: Edy, at first you are here just as a Technician. In five years time, you are making yourself to be=
Ss: Project Engineer
T: Project Engineer or Senior=
Ss: Engineer.
T: Why--why do we have to have: an ambition to achieve in five years time? Why you cannot be a Technician for five years?
Ss: Because--look forward--look forward
T: Someone wants--everyone wants to move=
Ss: =Forward
(45:00) T: Forward. Not just stick at one place, right? You still remember why usually hear job and career? Aha: what you should look for? A job or a career? A career can make you=
Ss: =Stronger
T: Stronger. And moving forward and become something that you know what you are. Can you answer it this way, ok. 'I have money. In five years time, I would like to open my own company.' Should I answer it that way?
Ss: No
T: Why not
Ss: Yes
T: Because the company will feel so: *tergugat*
((Laughing))
T: The company will feel: *tergugat*. What is *tergugat*?
Ss: Challenging
T: Threaten by you
Ss: Ah:
T: So they don't want to have you working for them because you may take all their secrets.
Ss: Information
T: And later build your own company. See!
Ss: No because (   )
T: So you cannot be honest about that?
Ss: Cannot
T: Cannot. (0.5) Ok, I know that sometimes, company: the company will like to have you for ten years at the same er: job position, right? But, the best way to answer this

question is by showing that you have some motivation to move forward. Ok? So, answer it: with whatever ambition that you have: if you would like to work temporary in the company, then you ask er you already give signal that you are here for experience, right? So it's not a problem at all. Ok, understand?
Ss: Yes
T: Last question is 'how do you think your qualification is relevant to the job?' How do you think that your qualification, which is Diploma of Electronic, ok, confirming, supposed to be relevant with the job. For example, you get Programming Technician, how does it relevant? Because you have learnt all the=
Ss:=Software
T:Yeah, software and programme engineering. Wiring programming, right? So, that's why you think that you are suitable for the job. Other than that? Suhaimi?
Suhaimi: Certificate
T: Certificate, ok because we have the certificate. So if we ask you again, what makes you think that you qualified enough for the job? Ok, you showed them your: results. Your transcript results and then your achievements for that certain subject. Ok? Is it clear enough?
Ss: Yes
(0.6)
T: I think that's all for today. I have covered on: what you should wear, ok. That's no. 1. And then, you should groom yourself and the last one is: the questions and=
Ss: =Answers.
T: Ok, don't worry, later for your job interview, you check some other questions as well. It will be to prepare and interview later on. Any questions regarding the lesson for today? No?
Ss: I think we are fine.
T: I'm so sorry we have that little problem just now. Ok. So I think that's all for today, the lectures. So I think yes. (48:57)

Transcription of Interviews

Recording No.: 17
**Notes:*
A: Interviewer
B: Interviewee

A: Ok, good morning.
B: Yes.
A: Cik Hanna ye, Cik or Puan?
B: Cik
A: Cik Hana, ok. Thank you for agreeing to talk to me. Since you already my--the purpose of my research is to investigate how code-switching is used in the classroom and to get some views from you what you think about code-switching .
B: Ok.
A: And it will be very confidential and it's only for my research purposes only this recording.
B: Ok, I understand.
A: Ok, what can you understand about code-switching?
B: Er: using: native language in class

A: Ehm:
B: To explain certain things
A: Ok
B: But not all the time.
A: Did you use--did you code-switching in the class just now?
B: Yes, I did ((laughing))
A: I realised that they are some. Was it
B: Some words like ‘shoe lace’ because they
A: They said rope rope ((laughing))
B: Yeah and ‘*mengancam*’
A: Ok
B: So, threaten.
A: Ok, alright alright, yeah yeah. Was it a planned ot an unplanned: teaching?
B: Depends on how the students answer my questions. So I think it’s unplanned.
A: It’s more of unplanned one, right?
B: Ehm:
A: So do you think, what other strategies dia you actually can use apart from code-switching in order for your students to understand?
B: Erm:
A: Is it only code-switching most of the time? Or there are other strategies that you used?
B: In class?
A: Aha: (0.2) to make sure that they understand.
B: Showing them video is another way .
A: Yeah, ok. Good visual.
B: Yes
A: Yeah, what would be your first and second languages then?
B: First is Malay and second is English.
A: Ok, so how long have you been teaching English?
B: Four years
A: About four years? Ok.
B: Yeah, still in the process of learning ((laughing))
A: ok, don’t worry. You’ll get there soon. So do you like to teach English subjects?
B: I choose English. I’m actually a Science students
A: Ok
B: But I choose English because I don’t like to learn Mathematics
((Laughing))
B: Anymore at tertiary education
A: I see
B: So I think yeah: other than Science all, Science subjects have Mathematics. Some sort yeah. I choose English.
A: So you choose English. Ok, just now you were doing code-switching in the class, right? Did you somehow feel guilty of using your
B: No!
A: Mother tongue. No, right?
B: No, because if you look at the students’ faces, you would feel whether they understand you or not. So, I think I felt more guilty looking at their sad faces not able to understand the: teaching at all.
A: I see. So, do you think you consider yourself as a proficient in English?
B: User?

A: Yeah
B: Erm: I still need practice and some improvements.
A: What do you think would be your strength as a lecturer?
B: I think to stay calm
A: Ehm:
B: Other than that, I would like erm: observe students' faces most of the time.
((Laughing))
A: What about your weaknesses? Do you have any weaknesses?
B: Erm:: I think weakness would be my language ability.
A: Ehm:

((Laughing))
B: I don't really able to make jokes in English.
A: So you are learning on how to make jokes in English
B: Yes yes
A: That's a good thing to start off, right?
B: Aha:
A: To improve your teaching.
B: I'm not really good with that.
A: Yeah. Ok, so you think that if you use code-switching, you might help the students to understand better?
B: Yes, some.
A: Is it different from this--other classes? Do you use code-switching in certain classes?
B: Not just different classes, in fact in the class, there are students who are very proficient in English, right. I know that they might feel: (0.2) uncom--uncomfortable if I use Malay in the class. But then, their classmates are not able to understand it.
A:Ehm:
B: So, it might not look nice to that one proficient boy, but er:
A: Actually
B: I have to make it balance.
A: Yeah
B: And I have to make that students understand that I'm using Malay to cater to their friends.
A: Who are weaker
B: Yeah: than that person
A: I see. Ok, what do you see our standard of English so far in general. Is it getting better or?
B: Most of the students here when they took MUET, it's band 2 or 3.
A: I see.
B: So, er: it's proficient, right? It's just moderate English user. So I would like to state that yeah, they are moderate English user. They can understand you but to reply back in English would be a little bit of problem for some.
A: Ehm:
B: That's why just now I said that I code-switch depends on how they answer my questions.
A: I see. Do you see that--you have been teaching for how many years just now you said?
B: Four
A: About four years. Did you think that you code-switch: before when you first teaching or now you code-switching more? Or is it just the same?

B: I think it’s just the same from the first year--I think the first semester I have er: very less code-switching compared to now.
A: Why?
B: The first semester, because I haven’t understand the students yet. I don’t know their level yet.
A: I see
B: But now, of course you have been very sure of their level, right.
A: Yeah
B: In fact, you start asking previous lecturers what did you give to these classes, what are the average marks for their English exam.
A:Ok. Thank you very much. I think that’s all for today.
B: I hope it helps ((laughing))
A: Thank you so much. If there’s anything else you would like to add, you can contact me later. (06:05)

vii) PIS Lecturer B Transcriptions

Transcription of Classroom Observations

Recording No.: 18
Date: 21/9/2014 (Sunday)
Time: 10.15-11.15am
Class: DFP 5C
No. of Ss: 9
Level: Mixed ability
Age range: 20-22 years old
Topic: Graphs and Charts (Describing Graphs & Charts)

**Notes:*

T:	Lecturer
Ss:	Students
(.)	short pause
(0.2)	2sec pause
[	overlapping speech occurs
↑	rise in intonation
↓	drop in intonation
--	change of the topic/rephrase/repeat
::	elongated speech/ stretched sound
=	latched speech, a continuation of talk
( )	words spoken here were too unclear to transcribe
(( ))	transcriber's description
Ø	no talk, ambient noise
U N	capital & space for acronyms
red	Malay words
' '	Sentences read from the board/book/reading materials

**Based on Jefferson, G. (2004)*

T: Ok, er: we'll wait for your friends. As they come in, they can join our class.
((Waiting for the students to come - 00:10 to 00:30))
T: Ok. Right, we'll start off--good morning class.
Ss: Good morning
T: Ok, what we're going to do today is erm: description of graph, ok? Now, before that,
((Ss coughing))
T: We have done a few exercises--some exercises on graph--for graph on how to er: identify once a while, x-axis, y-axis, bar graph, production, body, conclusion, right? What we're going to do today is we're going to: got through it again but today we'll focus on vocabulary. The words that you need to know, the terms that you need to know in order for you to describe the graphs, you need that, ok? Now, what I want you to do is er: you can work in pairs: or be in a group, no problem. We have--we have about 4 groups, ok? 3 groups. So, get into pairs or be in a group. Ok, do it.
((Ss sat in pairs/groups))
T: Ok. Here we have erm: about 37 words here. So, each of you will get about--each group will get about 9.
((Ss laughing))

T: It's ok. Take your word. You'll get extra if you're lucky.
((T distributed words to each group))
T: Ok, now what I want you to do is: Ok, now. What I want you to do is you need to group them into five groups. Ok? Now let's see what are the groups that we need to look into. Ok, now first, some of the words (0.5) they share the same meaning. Ok. The second (   ).
((T wrote the word groups on the board - 2:55 to 3:20))
T: Ok, now, you need to put tem into: six--six groups. Going up, going down. No change, going up and down, small changes, big changes and low points. Ok, what I want you--I give you about five minutes, what you need to do is you need to put those words accordingly. Ok? The words that you have, you have to put them accordingly. Ok? Now, if you do not know the meaning, it's ok. Guess. Yes, guess. And then we will look into the meaning. To find how do they--to find out the meaning. Alright, start. (0.8) Do you have any questions? Any questions? So, ok?
(05:00) ((Ss doing their work in pairs/groups - 4:20 to 5:30))
T: Try not to look at the dictionary first. Guess--guess. Try to guess it. What do you think it's used. (   )
((Ss continued to do their work in pairs/groups - 5:55 to 6:15))
T: Ok, feel free to write on that piece of paper. You can write on that piece of paper. Ok, don't worry about it. It's yours. You can send it later when you've done.
((Ss continued to do their work in pairs/groups - 6:27 to 6:40))
T: Can we start? Ready?
Ø
T: Ok, we have--you're done?
Ss: Yes. Just guess.
T: Don't worry about it. I guess all the time. (0.10) Ok. (0.3). Thirty seconds more. (0.4) Ten seconds more according to my clock. Eight, six, four, two, zero. Ok. Now, (0.2) what I want you to do now is, ok, when--as I go from one group to another group or pairs, you need to tell me where the erm: each word do you think belongs to that group. Ok? So we have three, right? Ok, any volunteers to write down the--who ones to volunteer? Who would like to volunteer? (0.4)
((A student came to the front to write the answers))
T: Maybe all of you will take turn. Let's just--ok, we go er: one group at one time. You just stand here.
Ss: Ok.
T: Right. What's your words?
Group 1: Open up, 'increase'
T: Only 'increase'? And?
G1: 'Increase'
T: 'Increase', that's all?
G1:And 'grew'
T1: And 'grew'. Ok, anybody else? From--the rest of the group? Done? No words to indicate: the verb going up? No? None? Ok, going down. You have one? Sure? Ok, what's that word?
G1: 'Went up'
T: 'Went up'. Ok, please write down. Ok now, what we are going to do is I'm gonna stop ((T clapped her hand)) talking. You are going to nominate your friend over here and you are going to inform her wheter your--the words that you have fits in there. Can you do that?
Ss: Yes

T: Ok, right. Er: girls. You can er: join in with group that one. There's--there're two of them over there, another one over there. So:
Ss: You can break one one.
T: Ok? Three groups only. Ok, I'll stop talking, it's you turn.
Ss: Ok
(0.4)
T: You have to facilitate--you have to ask your friend, ok?
Ss at the Board1: Anything for going up?
(10:00) Ss: Next--any more
T: Speak a little louder
SsB1: So another group?
G2: 'Decline'
SsB1: Hah?
G2: 'Decline' (0.8) 'Decrease'
SsB1: 'Decrease'
SsB1: Anything else?
G2: No
SsB1: Anything else?
G2: No
SsB1: Let's go to the next
Ss: 'Changing'
T: Ok, before we go to the next, next group, why don't you nominate another friend? To replace your place.
SsB1: Ok (   ) I would like to request my
T: First thing about this, don't ask question
SsB1: Ok. Norasmah
((Ss clapping))
T: Ok
Ss at the Board2: Any word for no change?
Ss: Stay the same--stay the same
SsB2: [Stay the same
Ss: [Stay the same
(0.8)
Ss: That is in B M, 'stabilise' is *stabil*. Stabilise is *stabil*. E I, *betullah, betullah*. L I
SsB2: L I
Ss: S E D. 'Stabilised'
SsB2: Thank you for helping me. Up and down. Any words up and down?
Ss: For no change we have: er: 'remain constant'.
SsB2: 'Remain constant'. (0.6) Next?
Ss: 'Undulated'
SsB2: Un? 'Undulated'
SsB2: Er: Anything else for no change?
Ss: No--no
SsB2: Ok next, up and down
Ss: 'Zig-zag' (0.7) ok, (gradually)
SsB2: Under no change, right?
T: Ok darling. Pick another friend.
SsB2: Ok er:
((Ss laughing))

T: Ok, this time around, I do not want to point at your friend. I you to mention this 'I would like to choose'
SsB2: I would like to choose: Afna
((Ss laughing))
T: Ok Afna. To do what?
SsB2: To: take my place (   )
((Ss laughing)
T: Ok
Ss at the Board3: Ok, can we go to small change?
Ss: No.
((Ss discussing))
Ss: (   ) Suddenly
SsB3: Hah?
Ss: 'Suddenly'. S U--S U D--D E N
SsB3: Hah?
Ss: D E N L Y.
SsB3: Alright
Ss: 'Steeply'. S T E--E P L Y
((SsB3 writing the answers on the board))
SsB3: Er: small change?
(15:00) Ss: 'Gently'. G E N: T L Y--T L Y--T L Y. Steadily--steadily. S T E--A S D, S D? eh, E A D I L Y. 'Slightly': S L I G H T L No--No--No. H T: T L Y.
T: Pick another to replace you.
SsB3: I would choose: Syafiqah
((Ss laughing))
((Syafiqah (SsB4) getting ready to go to the front - 16:05 to 16:25))
SsB4: Er: (0.4) right, 'reached a low' (.) small change or big change?
Ss: Small change--small change. R E--R E A C--R E--
SsB4: R E
Ss: A C
SsB4: C
Ss: H
SsB4: H
Ss: E D a low
SsB4: A low--A L O W eh, bukan?
Ss: *Haiyo! So lembabs*
((Ss laughing))
SsB4: A L O
Ss: L O W
SsB4: Oh ok.
Ss: Er: grew
SsB4: Next, small change or big change?
Ss: 'Gradual'--'gradually'
SsB4: 'Gradually'?
Ss: G R A
SsB4: Spell it, right.
((Ss laughing))
Ss: G R A er:: double L (0.2) *pandai*! Sharply
SsB4: Sharply
((SsB4 writing the answers on the board))

SsB4: Next?
Ss: ‘Feel’--‘fell’. F E L L
SsB4: F E L L?
((SsB4 writing the answers on the board))
Ss: Dramatic
SsB4: ‘Dramatic’
Ss: Drama:
((SsB4 writing the answers on the board))
T: Ok, Syafiqah. Why don’t you choose another friend to replace your place.
SsB4: I will choose er: Nadirah to replace my place.
T: Nadirah, ok.
((Nadirah (SsB5) getting ready to go to the front))
SsB5: For er: big change? (0.2) Anymore? For low ones?
Ss: ‘Bottomed out’
SsB5: Bottom up?
Ss: ‘Bottomed’:
((SsB5 writing the answers on the board))
Ss: E D. (0.3) ‘Bottomed out’
SsB5: Ok ok. Other than that?
Ss: ‘Flacted’--‘fluttered’ (0.5) F L U
T: Say louder louder
Ss: E R D--double T--E R D
SsB5: Double T
(20:00) Ss: E R D: after T (   )
T: Ok
Ss: (   )
T: Ok, anymore words: that we have left out? All ok? Orait, thank you darling.
((Clapping))
T: Thank you for your cooperation. Now, let’s look at these words. What I would do now is that I’ll not mention to you whether--I will tell whether it’s wrong or right, ok. I will let you know whether it’s wrong or right. But I won’t give you the answer for the wrong er:: words. So, then we have to discuss where it might belong to if it’s not there. Ok, any question? Right, ok now let’s look here. Going up, increase, ↑going up? Ok fine. (0.2) Yes, ok. Went up?
Ss: Yes:
T: Ok fine. Going down, drop. This one? Decrease?
Ss: Yes
T: Ok fine. How about no change? Stay the same?
Ss: Yes
T: Ok good. ‘Stabilise’?
Ss: Yes
T: *Stabil* -- *stabil*. So: *stabil* so anyone what does *stabil* in BM? What is it mean? Stabilise--stable, what does it mean? (0.2) Same meaning?
Ss: Still the same
T: Nothing change?
Ss: Nothing change
T: Ok good. Very good. Stable. Remain?
Ss: ‘Remain Constant’, *stabil*
T: Ok, remain constant fine. Undulated. Do you know what’s the meaning? No?
Ss: No

T: You know what, I'll--I'll--I was er: a big blur when I saw that word as well.
((Laughing))
T: Then I was like oh yeah so: that's not belong to this group, ok? It belongs somewhere else. 'Zigzag'?
Ss: Yes. Up and down
T: Why do you say 'zigzag'?
Ss: It's up and down
T: Goes up and down, up and down, correct.
Ss: Yeah
T: Ok, 'dramatically'?
Ss: ↑Yes!
T: Because it's your answer!
((Laughing))
T: Are you sure? Ok. How about the rest? You aren't in their group. What do you think?
Ss: It belongs to big change
T: It belongs to big change. Yes, very good. 'dramatically rise' or 'dramatically fall'. Oh my God! I'm not supposed to give you the answer.
Ss: Ouh: ala:
T: It's ok , it's ok, fine. I'm good.
((Ss laughing))
Ss: But it can be up and down also
T: It can be?=
Ss: =Up and down
T: Yes, but up and down one thing if you look into 'dramatically' is that unique. 'Dramatically'. Ok it can be dramatically ah: er:: increase dramatically or decrease dramatically, right? You are right. But you look into the context, what does it mean. Ok? Ok? Good. Right, suddenly?
Ss: Yes, because it's our answer.
T: Because it's your answer.
((Ss laughing))
T: What about the rest? 'Suddenly'? ↑'Suddenly'! Is it stable? Compare it to the other--sorry, is it--is it go up and down?
Ss: No
T: Compare with the answer here. ↑No. what does 'suddenly' mean?
Ss: (   )
T: Yes, so what's (   ) mean?
Ss: Sorry, we don't know. They are many idea here take up or down.
T: Ok, when do you think it fits later then working to death. Don't dream.
((Ss laughing))
T: How about this, 'steeply'? (0.3) Still don't believe me?
((Ss laughing))
T: Anybody else? 'Steeply'--'steeply' ok. What does it mean?
Ss: Step
T: Step or steps. (0.3) No idea? Anybody else? (0.3) Don't worry, it doesn't belong here. Ok, 'gently', small changes? 'Gently'?
Ss: Yes
T: Yes ok. 'Steadily', small changes?
Ss: No
T: No? You're right. 'Slightly'? Small change? Agree? Ok. 'Reached to a low', small (25:00) changes? 'Reached a low'. Do you think something to--by small changes?

Ss: Yes
T: Something to do with?=
(0.3)
Ss: =Of going down
T: Being down, something: low, right? So: right, 'grew'? (0.5) 'Grew' means growing up.
Ss: Oh:
T: Right? Ok? Next, alright. Small changes can be up or down, it doesn't matter. It's just a change, we're talking about the chain. Ok, big changes gradually?
Ss: Yes
T: Big change? (0.5) gradually, big change. They asked me gradually. (0.4)
Ss: No.
T: How about the less? (0.2)
Ss: I don't know
T: I don't know. It's ok. No worries. I don't know it as well. Alright, 'sharply'?
Ss: Yes
T: Big change?
Ss: Yes
T: Ok, fell, big change? (0.2) Fell? Big change? Not big change?
Ss: No
T: No?
Ss: It's supposed to be going down
T: Supposed to be going down?
Ss: Yeah
T: Like your pants supposed to be going down. Maybe. Right, 'dramatically'?
Ss: Yes
T: Yes
Ss: Finally ((laughing))
T: 'Levelled off'? (0.4) 'levelled: off', ok. (0.2)
Ss: Yes
T: 'Levelled: off'. Low point. Levelled off is low point. Why? It doesn't matter. We'll know that later but it indicates big changes, 'levelled off'?
Ss: No
T: No? You don't think so? why you think ask me. 'Bottomed out'? Low points?
Ss: Yes
T: Yes, very good. Why? Why do you say so?
Ss: Low point, 'bottomed out'. ((Laughing))
T: Low point, 'bottomed out'. Yes, Erni. Such as?
Ss: Because it's our answer ((laughing))
T: Because it's you answer. 'Fluttered'? How about 'fluttered'? Do you know what it means? (0.4)
Ss: Don't know
T: What does 'fluttered' mean? Hm: what does it mean? No--no idea? No worries. We'll look into that later. Ok, now, look at all those words that I've marked with that--with the blue mark. 'Undulated, dramatically, suddenly, steeply'. Ok, since 'dramatically' is over there, so we understand there's a big change, right?
Ss: Yeah
T: Forget--forget about that. 'Suddenly, steeply, steadily, reached a low, dramatically, fell, levelled off'. Now, what am I going to do is, I'm gonna explain first what's going up means, going down means., no, change, up and down, big change, low point ↑again.

From there, try to guess again these words. Ok? Going up means it goes up. Ok? It goes up. Going down, it goes down. Right, now no change it means it does not go up or down. It's just stays the same. At one point it doesn't change. In Malay, can anyone tell me, a word that you--not same as the word--word that means does not change. It starts with the word 'M'. 'M' *jugak pun ah:*
Ss: *Mendatar*
T: *Mendatar*. Yes, *mendatar*. That means no change, right? Ok, good. Right, up and down, 'zig zag' it means it's not stable. Ok, it's not stable. And suddenly you gonna have a heart attack. Is your pulse stable? No, right? Goes up and down, up and down, up and down. So what if very loose? What happens to your pulse?
Ss: *Mendatar*.
T: Ok, *mendatar*. What do you call it in English? No: change, right? You know that beep? It goes up and down, up and down, up and down, you're still alive but not stable, right? But not stable. So, if it stops?
Ss: Teettt ((sounds of beep))
T: Teettt ((sounds of beep)). Are you still alive?
Ss: No.
T: No, but the line is? The same position, stays the same unless somebody revises you. Then it goes up and down, up and down. Ok? Same concept. Remember that. Ok? Now, small change that is increases or decreases, increase or decrease in a small number--amount. Ok, it (30:00) can increase slowly, little by little by little by little, it doesn't matter. It can increase a little or go down a little. No problem. Ok? Big changes. All of a sudden. Increases and there are also decreases. Ok? Now, er: low point. Low point is that it's a bottom ((T slammed hand on the table). Ok, like I guess to explain to you about erm: the melting point of ice. We learnt about that, right? Ok, melting point of ice is? Water--ice my--water--water. Boiling point of water is? 100. Freezing point of water is? 0. Room temperature water is?
Ss: 30
T: 35, 37. Right? Ok, what does that indicate? Once the water reaches: 100 per cent, is it go higher--100 degree, is it go higher? After 100, is it go higher?
Ss: No
T: No, right? Once it reaches 0, does it it go any lower? So, what is this? This is ↑a: peak. So, if this is a peak, this will be the? (0.4) If this is the peak, this should be the?
Ss: Water
T: Yes, water. Very good. (0.3) Ok, it doesn't change anymore. That's like the maximum, right? Ok, fine/ Low point is the lowest point. It doesn't go any lower than that. Ok? 'Fluttered'. What does that mean? (0.2) No idea? It's ok, fine. Now, I will allow you to look in your dictionary. You famous dictionary. Your iPhone's, your Samsung's, ok? Right. Check it, and then explain to me, tell me er: explain to me what it means and why it fits that group. Very quickly we've got 2 minutes only because we have something to help you all this. Not 2 minutes, 1 minute. Can you quick. 1 minute, 1 minute, 1 minute! Ok, if that the case, I will divide it. Erm: (0.2) Shafiqah (0.2) Shafiqah, 'undulated, suddenly'. Ok, er: Afna's group, Afna where are you? Afna's group sorry. Afna's group, 'steeply, steadily'. Right? And
Ss: Bella
T: Bella' s group, 'reached a low, gradually', ok? And then Shafiqah's group again, 'fell'. Afna, 'levelled off' and 'fluttered'. Bella, 'fluttered'. (0.4) Ok, you have to--each of you try to get the meaning.
((Ss looked for answers in the dictionary - 33:20 to 34:20))
T: Ok, done? (0.3) Done? No? Not yet? Ok.

(35:00) ((Ss continued looking for answers in the dictionary - 34:30 to 35:24))
T: Ok, right. ‘Undulated’. Shafiqah’s group. What do you think it means?
∅ ((Some students will continued discussing in their groups))
T: No--none? It’s ok. Don’t worry. I’ll help you with that.
Ss: Ok.
T: Ok, ‘suddenly’?
Ss: ‘Suddenly’
T: ‘Suddenly’ is?
Ss: Big changes
T: Suddenly is under big changes. Why do you say so? ‘Suddenly’ under big changes, why?
Ss: Tiba-tiba
T: Tiba-tiba? ‘Suddenly’?
Ss: Ah: ‘suddenly’, yeah.
T: So usually when--when there’s a sudden movement, it should be a big movement? It’s will change.
Ss: Yes, sudden movement.
T: Sudden movement.
Ss: Yeah
T: Right, (   )
Ss: Yeah
T: Yeah (*Romset?*)
Ss: Oh: ((Laughing)) Yeah, (*Romset*?)
T: (*Romset*) (   ). Ok?
Ss: Ok
T: Ok, fine, very good. Big changes, ‘suddenly’. Because it happens so suddenly, it’s a change, big change. If it happens little by little, we don’t notice, right. Small changes er: small changes. Suddenly tomorrow I come to class wearing a blue wig--a blue wig, right? Is it a small change or a sudden change?
Ss: Sudden change
T: Yes, because I hardly colour my hair. Suddenly, I come to class in blue wig. ‘Suddenly’, right? Ok, very good. So, big change. Then, how about er: ‘steeply’? No, you are not supposed to doing it, you are supposed to ‘fell’, right?
Ss: Yeah.
T: How about ‘fell’?
Ss: *Terjatuh*
T: *Jatuh*.
Ss: So, er: it’s supposed to be er: back to a point or going down.
T: Going down?
Ss: Yeah
T: Ok, what do you think? Ok, good. ‘Fell’ means? This, right?
((T dropped her pen on the floor))
T: Right? So, in those=
Ss: =Big jump
T: Sure?
Ss: Not sure--sure
T: (*Romset*). Ok, good, next group. ‘Steeply’ erm: we’ve done with a ‘undulated’, ‘suddenly’. ‘Steeply’--‘steeply’.
Ss: Er:
T: Yes

Ss: 'Steadily'
T: You wanna go 'steadily' first? It's ok. No problem. 'Steadily'.
Ss: Er: no change
T: No change. Why do you say no change?
Ss: It's steady:
T: Why? Why--why--why? Because it's steady? Steady?
Ss: Because it maintain
T: Maintain, very good. What do you say 'steady' in *B M*? 'Steady'? *Steady:* (0.5) Alright, ok. Similarly with word we learnt just now, *mendatar*. Alright. 'Steady', ok. Right, erm: (0.6) How about erm: you have 'steeply', 'steadily' and 'levelled off', right? What about 'levelled off'?
Ss: Er: (   )
T: Sorry?
Ss: Low point
T: Low point? (0.3) Sure? Lowest point? 'Levelled off'.
Ss: Going down
T: 'Levelled off'. What do you mean by that. 'Levelled off'? (0.4) What's levelled? What is level? Same level, different level, what is level?
Ss: [*Taraf*
T: [No change, right? *Taraf*? *Taraf*? Level? Ok, status--*taraf*. Level?
Ss: *Tahap*
T: *Tahap*. Ok, so you reach a level, 'levelled off'.
Ss: No changes
T: Very good. Ok, so 'levelled off', right? ((T wrote the word on the board in the right column)). Ok, good. (0.3) 'Fluttered'?
Ss: Desperate
(40:00) T: I give the easy one--easy one, 'reached a low'. What's 'reached a low'?
Ss: Supposed to be going down
T: Supposed to be going down? 'Reached a low'. What is the word 'reached' means? Reach me. Reach.
Ss: *Sampai*.
T: *Sampai*. 'Reached a low'. *Sampai* a low.
Ss: Low point
T: Yes, why? Because it reached: a: low: level. 'Reached a low'. It's not that much, is there. Guess the meaning first. Ok very good. So, it 'reached a low', it cannot go any lower, right? That's the meaning. 'Reached a low'. It reached a level that it cannot go any lower. Lowest, so it goes under: low: points. (0.5). Ok, 'reached a low'. 'Fluttered'.
Ss: 'Gradually'
T: Oh, 'gradually', sorry. 'Gradually'. (0.3) 'Gradually' yeah?
Ss: Going up
T: Going up, 'gradually': sure? I say no.
Ss: *Secara beransur-ansur*
T: Ah: what is gradually in Malay?
Ss: *Secara beransur-ansur*
T: Ok
Ss: Small change--small change
T: Why do you say so? Goes up by little by little. *Beransur-ansur*, that's very good. Where to--where?
((Ss laughing))

T: Erm: small change. (0.5) How did you grow up? Today 100cm tomorrow 400cm. No, right? Grow up gradually. Small changes. Right, ‘fluttered’. (0.7) ‘Fluttered’. Yes?
Ss: Up and down
T: Up and down. Why do you say so?
Ss: *Berdebar-debar*
T: *Ber--*?
Ss: *Berdebar-debar*. Fluttered hearts
T: *Berdebar-debar*?
((Ss laughing))
T: ‘Fluttered’ is *berdebar-debar*, your heart? You heart flutters. What else flutters?
Ss: Up and down
T: Goes up and down. Ok, find your heart let’s try and see and compare to butterfly. Butterfly has been seen fluttered. So you?
Ss: Up and down
T: Very good. Ok, compare it with your heart, find ‘de de de’ ((T making sound of heart beating)).
((Ss laughing))
T: Right, ‘fluttered’ is up and down. (0.4) Ok, how about ‘undulated’?
Ss: ‘Undulated’ I think is as small changes.
T: Aha:
Ss: Maybe it’s a smooth, a smooth wave
T: Smooth: wave?
Ss: Ha:
T: Ok, fine. Smooth--you say what is that again?
Ss: Wave
T: No, the answer.
Ss: A smooth
T: ‘Undulated’?
Ss: Small changes
T: Small changes. Ok, smooth, smooth wave. How does wave move? What is the wave like?
Ss: Up and down. Sorry, Oh ok, sorry:
((Laughing))
T: It’s ok you all. You are almost there. Fine
((Laughing))
T: Ok, so ‘undulated’ sailing, small wave, goes up and down. It can be smooth. Ok, compare--compare here ‘zig zag’ sharp. ‘Undulated’ is more smoother. Sorry, more smoother--a little smoother. Ok, right it’s smooth. Ok. Anymore--any questions? Ok, what other words we haven’t solved? Er: one more. All done. Sure? (0.4) ‘Steeply’? Who’s supposed to do? Anybody? Yeah, ‘steeply’.
Ss: We almost forgot
((Laughing))
T: ‘Steeply, steeply’. Yes?
Ss: Er: big change?
T: ‘Steeply’ big change? Why do you say so?
Ss: ‘Steeply’ means *curam*.
T: Ok, so *curam*. How does *curam* look like? I don’t know.
Ss: Like that
T: Like that? ((While showing hand signal)). Slanted. Sharp. Ok. Steep, right. We can also say ‘sharply’. Say steep. Ok, fine. Very good. Big change. See, how easy it is.

Ok? You're (45:00) right. Let's do the answers. (0.12). Yeah, yeah, yeah, ok. Ok? I'm so sorry. Ok. Going up, 'increase', 'grew', 'line up'. Ok, another word 'rose'. 'Rose' is the past tense of 'rise': R I S E. 'Rose' is the past tense ↑of: 'rise'. Not rice that we eat and flour that we see. Ok? 'Rise', S E not C E. They grow, almost same spelling different meaning. Going down. 'Decrease', 'fell', 'drop', 'decline'. Ok, we're good. Good to go! Stay the same. Stay the same or staying the same?
Ss: Stay the same
T: Stay the same, ok. 'Stabilise', right? 'Remain constant', ok. 'Steadily' and 'levelled off'. Why 'levelled off' is not--Why 'steadily' is not under no change?
((T bangs the table to get Ss' attention))
Ss: Ouh: *mak*:
T: Why 'steadily' is not under no change?
Ss: Small change.
T: It's under small change. Why?
Ss: Just feel like--maybe going up and down.
T: Maybe going up and down.
Ss: Maybe it's just: calmly.
T: Calmly?
Ss: Yes
T: Slowly?
Ss: Slowly sometimes
T: So small change? So we write here. Are we? Are we right? Ok, 'steadily', small change. Because 'steadily' you still increase, you still decrease. Right? It does not just stop, no more. Still decrease--decrease, but in a small level--small amount. Ok? Can? Right, good. Now, let's look at no change--up and down. 'Fluctuated', 'zig zag', 'fluttered', 'undulated'. 1, 2, 3. (0.3) 'Undulated', 'fluttered', 'zig zag', one more? 'Fluctuated'! Ok, 'fluctuated' another way, ok. Small change, 'gently', 'gradually', 'slightly', 'steadily'. Ok, good to go. Big changes, 'suddenly', 'sharply', 'steeply', 'variety', and other word not here, 'a lot'. Ok? Low point, 'bottomed out' and 'reached a low'. Any question?
Ss: No
T: Ok now, I've given you an exercise here. Ok. In this exercise, what I want you to do is you go home, practice on this, answer the questions and we come to next class, we going to use these, whatever that you have done here, you're gonna practice and if they are mistakes. And then, we gonna use the same concept to do a few more exercises, ok? I'll give you a few graphs which has all these lines and movements. So you'll have to describe them. So you try out this worksheet first. See whether you can do it. Right? And then we'll look into it. Any questions? So, we're ok with the lesson today? Good to go? Alright, I'll see you next week. Have a nice day. Goodbye.
Ss: Goodbye.
T: If you wanna--you can take a photo what I've written here. Snap a photograph you keep or can ask me the list later, if you want to. Ok? Fine, thank you. Bye bye. (49:00)

Transcription of Interviews

Recording No.: 19
Notes:
A: Interviewer
B: Interviewee

A: Ok, good: afternoon
B: Yes
A: Thank you for agreeing to talk to me.
B: You're welcome.
A: I'm sure you already know that my research is to investigate the use code-switching in the classroom.
B: Ah:
A: I just want to get some views from you regarding code-switching
B: Ok
A: In teaching English in the classroom. So therefore our talk will be confidential and only be used for my research purposes, right?
B: Ok
A: Ok, so er: I'm sure you know what is code-switching?
B: Yes
A: Do you have any idea what code-switching is?
B: Code-switching is when you switch languages when you teach. So it can be a word, it can phrases.
A: Alright, so did you do any code-switching just now in the lesson?
B: Yeah, I did.
A: Ehm: was it a planned or was it an unplanned one?
B: Er: it is an unplanned.
A: Why did you think you do that?
B: Ok, erm: when we code-switch--when I code-switch--code-switch usually I will speak in English but I'll my best to--to er: in order for me to transfer the meaning,
A: Ehm:
B: I need to get them--I need to find out whether they could identify the words ot not, or if they would like to er: say out the word--the meaning of the word or--of that particular word that they do not know how to say it out but they know in their language--their native language. So, I'm able to the message out and the I'll tell them. This is actually the meaning of it in English and then it facilitate their learning.
A: I see. So apart from code-switching, is there any other strategy that you can use in order for them to understand?
B: Yes, more er::Other than code-switching, we have er:: I encourage them to: ask their friends for help like a while ago. What we did was, they try to guess first
A: Yeap
B: And then from guessing, they are trying to identify the meaning. And then to explanation. Last, look--only then they will look into dictionary. They look up the word in the dictionary.
A: Ok. So what would be your first and second languages then?
B: Sorry?
A: Your first language?
B: Er:
A: Your mother tongue?
B: My mother tongue is Tamil.

A: Alright, so your language--second language will be B M or English?
B: English
A: English ((laughing)). Alright, so the subjects that you are teaching here?
B: Er: three subjects. I'm teaching M U E T--MUET, and I'm teaching AE101 Communicative English
A: I see.
B: And Semester 5 (AE 501): Communicative English Skills.
A: How long have you been teaching here.
B: Here, four years
A: About four years, so alright. Do like teaching English?
S: Yes, I do. I'm enjoying teaching English.
A: Ok, sometimes people said they feel a bit guilty when they used B M in the class, do you feel bad:
B: No. If I use *B M* continuously through out the lesson, of course. If I encourage my students to answer in *B M*, yes of course. But if I encourage them to thin in B M, but tried their best to speak in English, I don't think so.
A: Alright, do you consider yourself to be proficient in English?
B: Err: as proficient as a native speaker, no. but proficient yes. Teach yes!
((Laughing))
A: So what are your strengths as a lecturer?
B: As a lecturer, I think I have classroom control
A: Ehm:
B: And I tried my best to er: when I teach my students, I do not want other factor to influence like er: students' er: what do you call them, whether they comfortable in class with me, whether they feel like too fierce or I speak too fast, so I try to elevate that type of problems, so my class I focus on understanding whether they make mistakes or not, it's not a problem, then we can fix it.
A: Do you have any weaknesses?
B: Weaknesses? Yes. At times when I'm too excited, I speak too fast and I have problem with time control. Yeap, and I can't manage my time now--at times.
((Laughing))
A: Is there any part of your teaching that you would like to improve?
B: Well, erm:: the use of technology and also the variety.
A: Ehm:
B: Like we have three main topics that we need to teach and then we have three hours--three contact hours per week. The problem is even though we have gone through the topic, after you repeat the topic, about the or 4 even a month time, the students' tend to forget what they have learnt.
A: I see.
B: So the reinforcement is not there. The continuous needs for them to know and learn that particular language is not there. So they just study for that particular course and then they--they don't study anymore. So, I--I encourage my students to learn how to learn the language.
A: I see.
B: So if it's like in the future, you'll still know how to learn the language on your own.
A: Ehm:
B: So I teach explicit grammar in the class which is not in the syllabus but I tried to encourage them that if you do not know the sentence structure, what do you do to try--or what can you do to improve on your own not with me around.
A: Yeah, they don't depend on us so much, right. They should know.

B: Yes. I tried to be the facilitator not only in the class, but in their learning to help--to understand that they need to know and how to actually--by right most of them, actually most of them, they are good. The thing is that if even though they want to improve, their second language acquisition, they have problem because they are--they do not know how to. Like us we have through B.Ed TESL and know how to.
A: Yeah
B: They do not know--they do not know the basic. So we encourage them--I encourage them to let them learn the language on their own. So code-switching, is in a way important for us.
A: Yes, it helps
B: It does help somehow.
A: Ok, so the last question, what do you think of English language in general, perhaps the students' proficiency is it better compares to the first year you are teaching until now or is it?
B: Er: it's improvement in time, but it also depends on the students' syllabus when they went they went--during their O-level, like some of them they did Science and Math in English.
A: Ok.
B: Their exposure to language is more.
A: Yeah
B: So when they come or--when they graduate and they enter polytechnic, their proficiency is a bit better.
A: I see
B: Compared to those are--who have less exposure
A: Experience. Actually see the difference [right, between this intake
B: [Yes, I can see whether they have improved or whether the proficiency have decreased. Like this semester, these students Semester 5, Semester 3, 2 yes, but the latest Semester 1 are not so good.
A: Oh:
B: Not so, not all of them.
A: I see.
B: They still have problem in speaking.
A: I see
B: And the language acquisition. You can see a slight bit but not--not that
A: Because is it of the change of P P S M T I thing?
B: Yes, it does effect. It does.
A: Ok, thank you so much for talking to me. So, if you have anything else to add, you can contact me later.
B: No problem.
A: Alright. [Thank you.
B [Thank you very much. (07:27)

viii) PIS Lecturer C Transcriptions

Transcription of Classroom Observations

Recording No.: 20
Date: 22/9/2014 (Monday)
Time: 11.15am-12.15pm
Class: DEP 5B
No. of Ss: 19
Level: Mixed ability
Age range: 20-22 years old
Topic: Job Interview

**Notes:*
T: Lecturer
Ss: Students
(.) short pause
(0.2) 2sec pause
[overlapping speech occurs
↑ rise in intonation
↓ drop in intonation
-- change of the topic/rephrase/repeat
:: elongated speech/ stretched sound
= latched speech, a continuation of talk
( ) words spoken here were too unclear to transcribe
(( )) transcriber's description
Ø no talk, ambient noise
U N capital & space for acronyms
red Malay words
‘ ’ Sentences read from the board/book/reading materials
**Based on Jefferson, G. (2004)*

T: Assalamualaikum and good morning everyone.
Ss: Waalaikumussalam
T: And please say hi to Puan Mazlin
Ss: Hi
T: So, he also asked me, don't worry. Alright, so before we proceed with our lesson, let's recap what we have learnt in previous class. Ok? So, er: first we have learnt about the resume, right?
Ss: Yes
T: Ok, good. Then, we'll--we learnt about
Ss: Cover letter--cover letter
T: Cover letter. So have you done with the draft?
Ss: Yes.
T: Ok, so later we'll look at your draft. Ok, so today we're going to look at the job interview. The preparation of the job interview. Ok?
Ss: Yes
(0.6)
T: Alright, so have you applied or attended a job interview before?
Ss: Yes

T: How many of you have attended any job interview?
((Ss raised their hands))
T: Ok, you. (0.4) Ok, Hafizul. Ok, do you have any personal preparation?
Hafizul: No.
T: What do you mean by no?
((Ss laughing)
T: I mean personal preparation like it's about grooming, if you find a beautiful--nice er: shirt, ok. So have that preparation.
(   )
T: Ok, how about you Ashari?. How about you?
Ashari: Yes
T: What do you wear for your job interview
Ashari: Er:
T: Ha: personal preparation, what did you wear?
Ashari: T-shirt with a shirt
T: Shirt, ok
Ashari: Like this, like this.
T: Oh ok. Just t-shirt?
Ashari: Ha:
T: Ok, what kind of job that you apply?
Ashari: Er: Sales Assistant
T: Where?
Ashari: Giant Hypermarket
T: For how long?
Ashari: 7 months
T: 7 months. That's quite some time. How about girls?
Ss: No
(   )
T: How about scholarship interview?
Ss: Just apply
T: (   ) Scholarship no? Ok, alright, never mind. So personal preparation like you'll go cut your hair: and you have something nice. Ok, er: nice clothes, nice *baju kurung*, ok? Personal preparation. Yeah. Are you, ok? Ok, so: what other preparation that you make for the interview based on your understanding of the company? Alright, so: Ashari, You applied Sales Assistant at Giant, right? So what do you know about Giant? Ah: Giant Hypermarket. So, what do you about Giant Hypermarket?
Ashari: Giant is:: an international company
T: An international company, ok.
Ashari: Erm:
T: What do they do?
Ashari: They sells--sold--sale
((Ss laughing))
T: They sell, ok. What kind of item that they sell?
Ashari: (0.3) Er: grocery
T: Do you know what is grocery?
Ss: Yes--yes
T: Beside groceries?
Ashari: Er: frozen
T: Frozen food, ok.
Ashari: Erm:

(03:50) T: Frozen food, canned food, as well as toys.
Ss: Don't buy anything cheap today. *Suka makan yang ini*? Ok?
T: I'll set the date for them to come, maybe you can get your friend also.
((Ss laughing))
T: So, what is the purpose of having job interview? This we are--till we are exchange for information. What kind of information?
Ss: Excellent
T: Personal information about whatever. Ok, what else?
Ss: Company background.
T: Company background maybe you want to ask location about the company background. Besides that?
Ss: Salary? Ay yes, besides salary. Mission and missions for holiday.
T: What did you do for? Ok, the job responsibilities.
Ss: Yes
T: Ok, job responsibilities. If you are applying for a Technician then you should know is just (05:00) opportunity of the technicians. For example--for example of responsibilities of a Technician. Ss: (   ) or program.
T: Did he say why is he like that? Besides that? Trace the (   ). Ok ye. Never mind. Alright, in an exchange of information . Exchange means er: to exchange information, to er: a person attending and interview, a person attending an interview, which is interviewee and the person asking questions in an interview, the interviewer.so, let's you apply for the job then you are ↑the?=
Ss: =Interviewee, the candidate
T: The candidate, beside candidate you can call it as?
Ss: Interviewee.
T: Interviewee. Ok? *Orang yang sedang* interview. Ok? *Dan* interviewer is the person who ask you ↑the?=
Ss: =Questions
T: Ok. Now can differentiate between interviewee and interviewer, right? Let's say I come for the job interview and you are the?
Ss: Interviewees
T: I come for the job interview then you are the? Interviewer, right? So, first we answer the questions post by the interviewer, so we need to answer the questions by the interviewer in order to impress the interviewer. Impress means?
Ss: *Menarik*--wow, impress
T: Yeah impress
Ss: To make it wow
T: This interviewee is really er:
Ss: Wow wow
T: What do you call that, er: have potential to be a staff, ok? And as the interviewee, ask question to obtain relevant and sufficient information. So, as your part, you can ask also ask some questions that are related to the job that you applied and to get sufficient information such as what kind of questions you can ask?
Ss: Salary
T: Why do want to ask the salary?
Ss: But
T: Because normally I think in the job advertisement, right, they have the job scope. Er: salary range is based on your qualification and also experience. Ok?
Ss: Benefit?
T: Yeah, benefits. Ok.

Ss: Join the company
T: Benefits of joining the company. Alright. Next one. Let's look at the appearance when you want to attend for a job interview.
((Ss making noise))
T: Ok, so do you think that interviewee is the proper way in the job interview?
Ss: No
T: Why?
Ss: Because not wearing a proper clothes
T: Not wearing a proper clothes. Ok, in your definition, what is a proper clothes?
Ss: Formal--formal
T: Formal such as?
Ss: With a tie, long sleeve ((Too many speakers at one time answering the questions))
T: Wearing tie. Is this appropriate ((While showing a few pictures to the students)). Is this appropriate?
Ss: No, too many, rock *kapak*, too much.
T: This is for what kind of job?
Ss: Modelling
T: Yeah, but if you are applying for a model then it would be appropriate, right?
Ss: Yeah
T: because you want to show how to dress
Ss: *Stylo*
T: being *sai-tylo*
Ss: *Sai-tylo*?
(0.6)
T: So, how about these two people?
Ss: Yes
T: Is it appropriate?
Ss: Yes. The lady is not appropriate.
T: Why do you say it's not appropriate? Can you give me some reasons?
Ss: Erm: ((Laughing)). Wearing short skirts.
Another Ss: Short skirts?
T: Ok, let's see the description. Ok, first hair. Comb and style neatly. Ok? So, boys, ok. You need to make sure er: your hair short, and you style and comb the hair neatly.
Ss: *Botak*?
(10:00) T: *Bolehlah*, ok. How about makeup or accessories? You can still wear makeup for girls. Boys=
Ss: You don't have to wear makeup.
T: Hm: ok. Ok, cheekbone minimal ok, which is not too much ok, not too much of: blusher, not too much of --what else do you wear?
Ss: Makeup, eyeshadow
T: Makeup, yeah. Eye shadow *kiri kanan*, blusher too red, too pink ok?. Ok, men. Clear moustache and sideburn. Maintain a clean shaver or trim through. Ok, let's say this fellow, make sure you clean a bit to make it more =
Ss: = safety, *kemas*, smooth--smooth
T: Smooth? Or ( ) between your massage. Clear succulent, ok? Ok, men could wear a two--a formal clothing, with and jacket while woman who dress formal and wear a dress. Ok, so men can wear like this, like this ((while showing pictures)). Men, ok. Can wear tie, if you have jacket then you can wear, ok? And leather shows. Will go to this shoes--leather shoes later. And girls, ok, er: dress formal and wear a dress but ok, for Muslim, you can--you can still wear your hijab, your hijab of course you are wearing.

You wear hijab, right so you wear nicely and then er: but the way you do your hijab, nowadays we have a lot hijab styles
Ss: Hijabster
T: So make it er: minimal ok? Not too much of flower, ok? And how about the colour of the shirt? Or the *Baju Kurung*?
Ss: Not too bright
T: Not too bright, yes, not too bright, ok? Hah: not too what?
Ss: Many flowers
T: Not too many flowers? Not wearing contrast or too striking colour maybe. Just wear a very soft tone, blue maybe, purple, light blue, peach. Not hot pink, too striking, orange. Normal is to you but last time when I attend my interview, I wear blue, (   ) which is not so bright and not so dark, just nice. Just nice. Ok, woman who look presentable wear a knee-length or acceptable length. So man, of course *baju kurung* is very long, right? Ok, so man who look presentable in a pair of palette pants or trousers, ok? Pants or slacks. You can buy these slacks at Padini, Factory Outlet, buy one free one. Ok? RM69 you can get free er: extra one pants. And shoes. Wear proper--sorry, wear a proper formal shoes not too casual, such as sneakers or sandals. Alright, so you should wear something like this!
((Ss laughing))
T: Do you have one?
Ss: Selvi
T: Just like Selvi, ok. How about the girls? Do you have any ladies' shoes? A very formal. Which er: it should have at least 1 inch heels, at least. So, 6 inches of high heels then, you will *jatuh*. Ok?
(0.8)
T: No sneakers yeah. Ok? So, during our assessment later, I hope you have leather shoes. Nice one. Polish.
Ss: Selvi, selvi
(15:00) T: Ok, no sandals right girls, no sandals. Keep the ladies from the straps from shoes like this one. Next one, how to ace the job interview? How to ace the job interview? Successful in your job interview. Ok, first thing is gather as much information for the job you are applying. Ok make sure you study about the job position, position of the company's offer. Ok? What do they do? What is the er: responsibility of the job. It needs--gather as much information about the job that you are applying, ok? Maybe you can start from the website. That's why I asked to come up with a er: company's profile, right? Ok, make some research of the particular company. Ok, next one, look into the company. look towards their visions, missions, their products, ok. For example like Panasonic there are here in Pasir Gudang it provide some digital model, right? Revise your resume and be acquaint yourself with job experiences. Ok, you need to revise your resume, right? Ok, if you are applying for that particular job, so make sure the job position that you apply is: is there. Ok? Objective. Ok? Everything except your experience. I believe you have your Industrial Training, *L I, Latihan Industri*, right? Ok, so make sure you remember what you have done in your er: *L I*. Ok, so you can share your experience towards the interviewer. Ok, so they may know that this particular person can do this, can do that. Ok, so if they find the perfect post, er: shop can just use for their new staff. Ok, rehearse being interview. Rehearse, rehearse, rehearse. Ok, I know some of you when you are doing job interview, you are nervous, right?
Ss: Yes
T: Do you consult, rehearse already.
Ss: Yes--yes

T: Ok, where? In front of the mirror?
Ss: Yes
T: What kind of rehearsal that you do?
Ss: In front of friends.
T: Hah?
Ss: In front of friends.
T: Say louder, I cannot hear you.
Ss: In front of friends.
T: Inform your friends?
Ss: Yes
T: Oh: In front of your friends: ok, I thought inform your friend. In front of your friends. Ok, they will d some here also, ok? Use strong word and declare in your interview. So, provide text so that you are ready to come. Of course the gardener enjoy the Deepavali, right? What if you come late to the job interview?
Ss: Prepare yourself
((Ss laughing))
T: How's the impression?
Ss: ((Not clear of students' responses)) Not punctual.
T: Not punctual. He said that about audition is that, this—is not be punctual ok, what else? Come early, punctual . What else that can you do?
Ss: Proper etiquette?
T: Yeah I great them, ok, besides that? Wear nice shirts, make sure you change your nails, stocking, about your short makeup, ok? Ok, next one. Build a rapport with your interviewer in the beginning. Build rapport. Rapport means? Like er: rapport, what is rapport? Not report card
Ss: ((Ss laughing)) Rapper
T: Rapport is like you just you build relationship. You build a relationship a brief, no need er: details. Besides, the introduction is to the interviewer. You *kenalkan diri* you. So, to make your interview session like er: oh: you have dispense our connection with a surfer, ok? Born, relationship. So you can shake the hand ok, do some handshake. Er: man if you can shake (20:00) with a man then, you are er:: if women is the panel, it's up to you. It's sufficient choice, ok. Thanks Wan! Ask patient to interviewing work if you've got an opportunity. Ok, you stay here and ask questions. So what kind of questions do you think suitable we ask in an interview? Do you think asking salary is:: appropriate?
Ss: No--no--no
T: So what kind of questions?
Ss: Working hours.
T: It's already stated in the job advertisement. Try to avoid something that is already stated in company's website and in a job advertisement. Maybe can ask, is it possible if you being to re-locate to some other venue or some other (   ) you need to do in the company, do you need to do that? Because I also don't know when I was actually my interview, they were asking what if I send you to Sabah and Sarawak? Well, I replied, Oh it's ok, I'm still er: er: single. I can be there: cheh: at that time.
((Laughing))
T: Now, here I'm in Pasir Gudang, Johor nearby my hometown then. Subordinate. I need to be there. Ok? Alright. (0.5) Next one is who is going back to Malaysia can sake for the venue, ok. Always bring back questions to ask. Be prepared. How many should you think?
Ss: 5--2!

T: At least 2 ok. Ok, we have samples here to make judgement about grouping--about you ask this or the questions you asked. Ok, new judgement.
Ss: *Takde* pun?
T: Always be there. I usually leave there sometimes. But sometimes, you just keep it in your heart, right? Ok. How many questions to ask? Only two. And make sure you have done your homework. If you ask me to much questions, then they might think that, does this person do any research before they come for the interview? Ok? You should have done your homework; I mean the research of the company with the job that you apply. Ok, (0.5) know the nature of organisation and computer technology. Ok? The nature--the culture of the company. How do they work? For example like a Japanese company, right? You have early morning assembly, right?
Ss: Yes
TL Yes, you still remember in your life? In the interview. And then you will do your exercise. Ok. Do you like it?
Ss: No
T: But it's healthy what. Hah? Sometimes. And then 10 o'clock you are still you morning break, right? Yes, and *then susah sikit*? (0.5) So, that's an example of how they work in the company. (0.6) Ok, that's why some of the page actually answer in the company's website. So, I have asked you to get the company's profile. Ok, so er: we will asking for the division and mission, so we all already have in your mind. Mission and vision of the company, their products they have obtained, the service they offer to the clients, because we have default that company. So all the information should be in your hand. Ok? So, this one is landscaping, preparation and preparing small certs. Ok, so, anticipate, what is anticipate, anticipating. Hm:? Anticipate is before you parting, anticipate is?
Ss: Antiparticipate
((Ss laughing))
T: Not antiparticipate. Anticipate is like you are facing oh, this particular question is going to be asked in the interview. It means that you *agak, teka,* guess, predict. The conversation that you have. Ok, so always ask about the personal background, right? For example, question? The famous question.
(25:00) Ss: 'What's your name?', work experience
T: 'Tell me about yourself'. Your personal background. 'Tell me about yourself.'
Ss: ( ) new sign for patient.
T: Ok. Next one, qualification and experience. Ok. Er: 'we are in average wide field'
Ss: Hah?
T: 'Electrical Engineering in Communication, right?' and then your experience. Especially when we go *L I*, Industrial Training. Ok, then you'll get--so got ideas from the responses earlier, so I know what to reply when they asked questions or answering er: post questions. Ok? Next one the company and the job: applied. 'What the company is like, what do you know about our company?" Er: 'What is our future action of our company?' you already know because you have already make some research. Ok, othe questions, such as?
Ss: Hm:
T: Other questions, that you can ask?
(0.9)
T: Your strengths and weaknesses, er: your future
Ss: Future talk?
T: Yeah , future talk about five years from now, ten years from now. Er: or question like 'do you intend to pursue your study?' Pursue--continues your study. The interviewer

might ask question, 'do you intend to further your study'. Pursue--continue your study. For example like now you have a Diploma then you want to do your degree or Master. (0.5) Yeah, that's all. So, what I showed you today is something that you need to prepare for our assessment later. Ok please ye during the assessment, mock interview, you wear nice shirt, slack. Ok? Since you are going for interview, you *tak pakai baju tu masa inerview*! Alright? *Bukan macam sekarang*. You have your purple shirt and all that, ok? So you need to wear purple shirt. I think the shirt is ok but only the shoes. Make sure that you come with proper belt. Shoes. Lots of shoes. *Penat Limah*. ((Laughing)). ( ).
Ss: Yes
T: The first time you do research in Form 3?
Ss: Er: a school kat Bangsar. Shoes shop.
Other Ss: The shoes tip-top.
((Ss laughing))
T: Oh, maybe you can go home from your class, er: I'll pass this to your friends, ok? Do you have any problem?
Ss: No--no
T: No right. I think girls have lots of stocks of shoes, right, at your house. Ok, I think one or two are not enough for girls' shoes. Ok.
Ss: Can we wear safety boots?
T: What?
Ss: Safety boots.
((Ss laughing))
T: Ok, see you guys later. (29:05)

Transcription of Interviews

Recording No.: 21
**Notes:*
A: Interviewer
B: Interviewee

A: Alright, Assalamualaikum and good afternoon.
B: Waalaikummussalam, good afternoon.
A: Thank you for agreeing to talk to me. As you know my research is on code-switching, so I just want to get some views from you about code-switching to be used in the English Language classroom. So, this talk will be confidential and only be used for my research purpose, alright?
B: Alright.
A: So, what do you know about code-switching?
B: Erm: in my opinion, code-switching is very--code here means the language. So, for example during the lesson, so we switch the code to change for example now Malay language and English. So, sometimes we do code-switching to help the students to understand on certain terms, or instructions. Ok, it depends on circumstances. Ok, that's how I used code-switching in my lesson.
A: Alright, so did you do any code-switching just now in the lesson?
B: Yeah.
A: Quite a number, I noticed. Was it a planned or unplanned code-switching?

B: Erm: no it's not a planned.
A: It's not a planned
B: Because sometimes it's just
A: It's just come out
B: It's just come out.
A: Ok, is there any other strategies that you may use in order for your students to understand your teaching?
B: Ok, besides using code-switching, I may use--I like to give them a visual about something. So I will describe in a simple language.
A: I see.
B: In a simple language and something that voab--vocabulary something that they are familiarised with. So they would not have problem in understand still. Term or the instructions, ok?
A: Ok. What would be your first and second languages?
B: My first language is Malay language ad second is English, ok.
A: So how long have you been teaching English?
B: Erm: I think about 2 years
A: 2 years. So, you're still new, right?
B: Yeah, still new. A novice teacher.
((Laughing))
A: So how do you feel teaching during your first year here compared to the second year? Second year, any difference?
B: Er: during the first year a little bit nervous because I don't know about the--how's polytechnic--environment in the polytechnic, the syllabus for example because sometimes er: the syllabus is, I may not be familiar with the syllabus in polytechnic. And--and before that er: I did my practical in school so the syllabus is different. So when I came to polytechnic, I have, oh my God, I have to know about this kind of field, hospitality, engineering terms and all that. So I need to familiarise myself
A: Yeah. True.
B: with that. Ok
((Laughing))
A: Do you feel guilty or not of not using English all the time?
B: Yes.
A: Ok.
B: Because if I'm not using English all the times because in the classroom I always feel guilty because I'm teaching English, why should I speak in Malay?
A: I see.
B: But sometimes in certain circumstances, I will use Malay to help my students.
A: I see. So that is to use code-switching in order for them to understand, right?
B: Yes
A: Ok. Do you consider yourself to be proficient in English?
B: I don't think so because I need to learn some more because when I'm here in Polytechnic, I used a lot of Malay, I think, compared to in my university year because all my classmates are using English and here sometimes--we speak more with the students. And then the students cannot 'hah' cannot understand what I say then I have to *macam*, like tolerate with them in term of the language.
A: Yeah.
B: Because of the language barrier.
A: First thing that you always think that the students know English
B: Yeah

A: But later on you go down a bit, right?
B: Yeah
A: So what are your strength as a lecturer?
B: A strength as a lecturer, I think, I like to crack some jokes in class. ((Laughing))
A: Ok.
B: Ok, I think that's er: will make the classroom lively.
A: Yeah, true. I can see that just now.
B: Because er: because if you don't make jokes and then you always, for example like I heard some of the students said, this particular lecturer always er: focus on the lesson and didn't want to crack some joke. And it makes us boring because I feel bored in class. Ok? So I think that maybe I should do something, especially crack some jokes, create some stories and y experience with them, that is how I make the classroom interesting.
A: I see, that's good. What about weakness? Do you notice any weaknesses in you, as a lecturer?
B: Yeah, as a lecturer, sometimes I started in class. And then I try to--sometimes I cannot get the right word to say.
((Laughing))
B: Especially in English sometimes because--or even Malay, itself. Because maybe it's the lack of usage the particular language. Ok.
A: So, the last question would be what do you think of English language in general especially in polytechnic? How do find the level of English? Is it getting better? Is it at what it is expected for them to achieve when they graduated. What do you think?
B: Ok, at first when I first arrive here, I'll my first class would be like--the students my semester 3, ok. I notice that they are very week, ok. They are very week. Ok, and then after next semester, we received a very good students, freshie, SPM, ok. And these students are being fitted by the UPU and all that. So we received a very good one.
A: I see
B: And then, I noticed that they are good in English. However, when it comes to written, they are very ok with that. Written English ok, but in spoken, in oral, they are not so what I expected.
A: I see
B: Ok, when it comes--because in Communicative English, we do a lot of presentations, erm: role-paly and all that. I can see that their lacking off. Ok? I think maybe
A: Work on speaking skills more
B: Yeah--yeah. Speaking skills.
A: Alright, thank you so much for talking to me. So if you have anything else to add, you can contact me later. Alright.
B: Alright.
A: Thank you.
B: Assalamualaikum. (06:19)

ix) PIS Lecturer D Transcriptions

Transcription of Classroom Observations

Recording No.: 22
Date: 23/9/2014 (Tuesday)
Time: 8.00-9.00am
Class: DRI 5B
No. of Ss: 18
Level: Mixed ability
Age range: 20-22 years old
Topic: Job Hunting Mechanics (Job Advertisement)

**Notes:*
T: Lecturer
Ss: Students
(.) short pause
(0.2) 2sec pause
[overlapping speech occurs
↑ rise in intonation
↓ drop in intonation
-- change of the topic/rephrase/repeat
:: elongated speech/ stretched sound
= latched speech, a continuation of talk
( ) words spoken here were too unclear to transcribe
(( )) transcriber's description
Ø no talk, ambient noise
U N capital & space for acronyms
red Malay words
' ' Sentences read from the board/book/reading materials
**Based on Jefferson, G. (2004)*

T: Er: we start with a little bit of recap, a little bit of reflection on what we have done er: on Sunday as well as previous week. When we started talking about Job Hunting Skills or Job Hunting Mechanics, so we were talking about cover letter. We discussed at length *macam ada buat juga kan*? Ok, we were talking about the format: official letters. We were alking about the compulsory items (.) on the other hand, we were also discussing optional items like reference number optional and so on. And we ↑did discuss about preparing the C V, Curriculum Vitae or Resume. Ok. What is another (.) sound for this spelling? *Kalau kita kata* re(.)sume. Continue, ok. But this one is Resume: and I told you Resume is not fully-dependent on one particular format. And he compared, if I prepare Resume after working 30+ years, my resume will be rather difficult, *kan* than what you are preparing. And we identified the important items that you all as a new applicants for jobs (.) soon (.) we will have. Ok, we ended up setting you simple task.(.) Putting you in groups and asking you to collect three advertisements (.) for each group. Remember that?
Ss: Yes
T: Alright. So, we are moving on to discuss the advertisements that you have collected in groups. We will try and see if those advertisements fall under technical field or

business field or community field, language and communication field, tourism and hospitality or others that we can crunch later. So, you would have to tell me, the advertisements that you would have collected so far. (0.2) Shall we start from--you all one group, right? We start with you. What are the advertisements that you have collected? If you don't know how to put it into a headings, just try and explain what is there in the advertisements, and how have you collected that.
Ss: Hm:
T: The source--*sumber*. The source that you have collected the advertisement. *Dari mana, sumber mana*.
Ss: Internet
((T wrote on the board))
T: Ok, internet is one source.
Ss: Newspaper
T: Newspaper is another source.
Ss: Magazine
T: Magazine is another source.
Ss: WhatsApp
T: WhatsApp, ok.
((Laughing))
T: Oh you don't have anything to WhatsApp you apply for a job *ah:* Ok, alright. If that happens, we accept that. Some more?
Ss: The advertisement pasted on flyers
T: Flyers *ya* flyers. Six? You are walking around shopping parade, shopping complex
Ss: Billboard
T: Billboard, ok, billboard. (0.3) Billboard. Ok, when you see billboard it can be billboard outside the premise of an agency or it can be at the door of an agency and so on. Ok, we have eight sources which is good. Ok, enough for that. Er: we come back to your own group. *Dari mana* and *apa dia iklan itu yang anda telah cari*. Ok, so we start from you group. The first advertisement is?
Group 1: Hospitality
T: What is it about? What's the job?
G1: (   )
T: Come again, the job title.
G1: Kitchen Manager
T: Kitchen Manager, ok, Kitchen Manager. Hospitality we have one. (0.3) No.2?
G1: Graphic Designer
T: Graphic Design. What's the post? What's the job?
G1: Er: Excuetive Designer
T: Executive Designer. So where do you think is that? (.) What company is that?
(05:00) G1: Business
T: Ok, business, alright. Ok, next group. Your group?
G2: Business
T: Business, ok, what's the title?
G2: Applying job at Burger King
T: Applying Job ↑at?=
G2: =Burger King,
T: Ok, Burger King, business. Your group?
G3: Business
T:Business. Job title?
G3: (0.5) Graphic Designer

T: Graphic Designer, Business, Company name?
G3: (0.4) Urban
T: Urban, ok. Business. Right, next group? Same. The other group?
G4: Graphic Artist
T: Graphic Artist, which company?
G4: Artist Station
T: Where do you want to put that in? Business?
G4: Hospitality
T: Hospitality. Alright, second round. The next advertisement. Three advertisements, right? Ok, next one? Yours? (0.3) Yours? Second?
((Coughing))
G1: Management
T: Management, ok Management. Er: which field? *Bidang mana? Pengurusan bidang mana?* Which field? So where do you think you wanna put that? Or you want another one under this, management?
G1: Hm:
T: Ok. (0.2) Alright?
G2: Magazine Editor
T: Editor--Magazine Editor, probably communication. Probably communication. Just throw like this, you don't have to go group by group.
Ss: Flight Attendant
T: Flight Attendant. So, shall we put that under tourism?
Ss: Hospital--Service
T: Service, service sector, ok service. Service sector, ok. Good. Some more?
Ss: Photographer
T: Photographer, ok. Company name?
Ss: Photolicious ((Giggle))
T: So that would be probably?
Ss: Business
T: Business, ok. Business. Give me one or two more.
Ss: Public Service
T:Public Service, ok. Communication or public service. Two more.
Ss: Graphis Designer Assistant
T: Graphic Designer Assistant where?
((Ss discussion))
Ss: Service
T: KPJ, Nurse? We want to put there under service?
Ss: Under service?
T: Ok, Nurse where would you put? Or you wanna put at something else?
((Ss discussion)
T: Health (.) Health Service. Health: Service. Ok, enough. I believe there are because I said three per group, but automatically there will be some overlap. So, when we discuss or when we: try to find out what you have, what kind of placement, where is you source, what company, what's the job title, or name of the job, then you'll some overlaps hopefully. So no problem with that. Ok, we move on with our input. Whenever you look at or whenever you come across job advertisement, you will have some requirements (.) from you who is the future of the potential applicant. *Pemohon--bakal pemohon*, ok. You have certain requirements by that particular company in the advertisement. *Keperluan-keperluan dari diri yang akan memohon nanti. Jelas? Senyum sikit kalau* clear.

((Ss grinning))
T: Ok, *lagi ramai senyum*. Ok clear. So, I give you 10 minutes, not more than 10 minute ah: in (10:00) groups. Select one of the job advertisement. Select one, because after that you are going to tell me what advertisement, what's the job title, which company, and what are the requirements. So do that. I will go around. I'll go around and you can ask me question, if you want to. (0.5) One advertisement, then you will tell us--this class, the source, company name, job title, and the departments. You are considered Diploma holders ah:, dah--dah habis belajar. So you're applying, you're searching for a job.
((Ss' discussion with group members))
T: Discuss in English. Discuss in English. That has never happened. *Kalau* discuss *je*, *meletup Bahasa Melayu*.
((Ss laughing))
((Ss' discussion with group members while T went around to check Ss work))
T: If you come across these words, for example, er: Supervisor cum--delivery boy C U M. So in this phase or this contact, 'cum' here means dah 2 in 1. You are--when you--when you're taken--given the job, you'll be the Supervisor as well as the Delivery Boy. That's the meaning of 'cum'.
((Ss' discussion with group members while T went around to check Ss work))
Ss: Sir, our advertisement is that there are filthy.
T: Filthy, no problem. Ok, but definitely there'll be some requirements, right? Ok, in your case if you don't have any requirements written there, you invent, *agak-agaknya keperluan nya macam mana*. You try to be creative and think about, because you are going to apply, right? So you should know yourself. For this job, although the requirements are not stated, but what do you think should be your requirement, or should be your speciality as I told we look forward to by the title. So, *kreatif sikitlah*.
((Ss' discussion with group members while T went around to check Ss work - 13:10 to 13:20))
T: Make some notes so that you can tell each other
((Ss' discussion with group members while T went around to check Ss work - 13:23 to 14:50))
T: Start making notes. Five minutes more. Just start scribbling whatever you came across. (15:00) Expected requirements by the firm or agency (.) or the company. (0.5) Because you gonna put everything together, right. So that's better. (0.15)
T: Passed around a piece of paper for attendance. *Ramai takde ni*.
((Ss' discussion with group members while T went around to check Ss work - 15:48 to 17:05))
T: Ok, 2 minutes
Ss: ↑Hah
((Ss' discussion with group members while T went around to check Ss work - 17:07 to 18:15))
T: Ok, give a few from each group. Then we will pull everything together. A few from (.) each group. (0.4) Ok, first requirement?
Ss: SPM or Diploma
T: So what do you called that? What would be written in the advertisement? You said SPM and? Diploma. SPM or Diploma, isn't it? So, what do you call that? In the advertisement, it will be written as?
Ss: Education--education
T: *Ya, semua* mention. Qualification. Qualifi(.)cation, ok. Alright. So qualification is supposed to be requirement. Almost every advertisement that prefer, every company will state the required qualification or the expected qualification. Some company will

put S P M / S T P M / Diploma / Degree so that means the salary will match with the qualifications. Alright, next. Not from you. Next group. We'll come back to you.
Ss: Nationality
T: Speciality
Ss: Nationality
T: Nationality, ok. Certain--in certain cases yes. (0.4) Nationality. (0.2) *Bahasa Melayu apa ni*?
Ss: *Kewarganegaraan*
T: *Kewarganegaraan*. Ok. Because certain post, want to make sure that (.) you are either (20:00) citizen, otherwise they will put extra (.) requirements for foreigners. So they want to know. Ok. Speciality, what--what is written there?
Ss: Knowledgeable
T: Extra knowledge or speciality. ((T wrote on the board)) Extra knowledge or speciality because they have that in their advertisement. Ok, next.
Ss: Able to speak English and *B M*.
T: Able to speak, ok extra knowledge and skills. ((T wrote on the board)) We add skills here. Extra knowledge and skills. Able to speak Mandarin and English. Ok, your group.
Ss: Experience
T: Experience ((T wrote on the board)). Ok, experience. Another one from you. (0.6) Ok, tell you.
Ss: Personality
T: Personality. Ok, yes. Certain post do have (.) personality. Ok, what is required under special er: personality? What is written?
Ss: Self-motivated
T: Self-motivated:
Ss: Hardworking
T: Hardworking
Ss: Creative
T: Creative. Ok, fine, very good. (0.4)
Ss: Punctual
T: Puncuality under personality, yes. Alright, your group another one.
Ss: Fresh graduate
T: Fresh graduate. Er: how is it written? Fresh graduates are encouraged to apply. Alright. So ((T wrote on the board)) open for fresh grads, ok. Give me two more.
Ss: Height and weight
T: Height and weight? *Ada*? (0.5) Oh, because yours is the Flight Steward *ye*. Alright. ((T wrote on the board)) Height: weight. *Dia nk yang berat-berat ke? Itulah kapal terbang banyak jatuh ye*. ((Laughing)). *Berat-berat dia pilih*. Yeah, you see *ah* certain cases *ah,* because we're talking about Flight Attendent. Ok, good. Some more. (0.2) *Ada lagi yang satu dua perkara yang penting*. (0.6) Salary. In fact, many will ask for expected salary. (0.5) Give me one more.
Ss: Age
T: ↑Age. Ok, 9.Age. Last one. (0.2) Personal details, ok, of course.
Ss: Gender
T: Gender, alright. Age and gender I will put it here. We still have one more. *Takde yang minta gambar ye? Ada tak advertisement yang mintak gambar?*
S: Ada
T: So that's another (.) ok. ((T wrote on the board)) Of course, different firms could have mould what we have written. Could have. Just for discussion sake, ok. We have identified advertisement requiring the applicant to state the qualification, nationality,

extra knowledge, skills, experience, personality, stated as open for fresh graduate, encouragement, height and weight for certain cases, certain jobs, expected salary, age, gender, all those. (.) Ok, although some of these are found in the Resume, some of these are found in the Resume, but before that *ah*, not every job will require everything that we have written. Among the requirements, *di antara keperluan*, so all the requirements. Ok, we come back to--when these requirements or among the--these requirements are stated, some of these will be stated in your CV,
(25:00) Resume. Some of them may not (0.2) be there in the Resume. But most have *lah*. So, 2 days ago we spoke about writing a cover letter. it is strongly advised that write out in sentence (0.3) responses to the requirements. *Tulis dalam surat. Surat iringan atau surat kita memohon kerja. Respon kita, tindakbalas kita dari sudut ayat* (.) *dalam surat*. Write it in the cover letter. Ok, make a quick and see what we can write of. Qualification is quite easy to state. 'I am S P M holder, S T P M holder, Diploma holder'. Or 'I have Diploma in Graphic Design'. Ok, it stated that way. Nationality is 'I'm Malaysian'. Ok, 'I'm a Somalian', for example. Ok, extra knowledge. What are ((Ss coughing)) (   ) that you will probably write for extra knowledge?
(0.3) You *ada sebut satu tadi*. Somebody. Ok, language. 'I can speak Malay, English and Chinese'. (0.4) I maybe have, for example. ((Laughing)) Ok, or you can say 'I am good in command of 3 languages; English , BM and Bahasa Melayu and Mandarin or Tamil or Punja whatever'. Ok? (0.2) That's for language. What are the sentences could you or would you write for extra knowledge? What other thinks are considered as extra knowledge? (0.5) Knowledge in?=
Ss: =computer
T: Computer field?
Ss: Designer
T: Designer, ok. Whatever that is not stated as the requirement, bukan keperluan, but you feel that it will help you, it will give you more strength in application, extra knowledge *la*. Extra skills *la tu*. *Kan?* (.) *Betul? Macam tak faham je.*
Ss: *Faham*
T: *Kalau faham kena senyum, kalau tak faham kena kerutkan kening.*
((Laughing))
T: So, *kita kena fahamlah sebab senyum ye. Kan.* Ok, what sentence can it be for experience?
Ss: The experience in
T: 'I have'=
Ss: = experience
T: '2 years of experience working with--' Ok. 'I have experience in the er: washing and cleaning aeroplane'. *Mestlah, nak jadi Stewardess*.
((Laughing))
T: Ok, so we state the experience yeah. Ok, still on experience. In certain cases, although advertisement require you to state experience, but you don't have experience. What can you write or what should you write?
Ss: 'I am fast learner'.
T: 'I am fast learner'. 'I don't have experience but I am a fast learner'. Ok, 'I don't have experience but I am willing to learn and pick up the job requirements fast'. I always tell you *ayat bebas*. But, in here, the point is that *ayat* is up to you as long as it is correct. Alright, we (29:20) move on to personality. *Ada satu* word--one word we use for personality. It's start with P L E A
Ss: S A N T
T: Yang lain tunggulah

((Laughing))
T: P L E A S
Ss: A N T
T: Pleasant personality (0.4). I have a pleasant personality. Esok 10 sen, minggu depan 10 sen. Tak bawa syiling
((Laughing))
(30:00) T: Ok. A pleasant personality and then you said *tu yang* pleasant *tu maksudnya apa?* What do you mean by that pleasant personality? Pleasant personality *lah*. Ok, offer for fresh graduate. 'I'm a fresh graduate (.) but I come with extra knowldege it seems', for example. Height and weight af course nothing much to discuss. Expected salary. (0.5) See what firm you are applying for the job at. Firm *macam mana*? There are two opinions in the expected salary. Some peepler say just put minimum salary because easy to get the job but the other group says put a little bit of more expected salary because you are showing them that you have some extra knowledge to use and you can work for that normal job money. *Ayat kedua tak faham ke? Ok, ada orang kata letak yang less punya sebab mudah dapat (.) tapi* not always *ye*. You *letak minimum salary* certain firm will say 'why is she asking for so little? She has no confidence in her'. *Tak ada keyakinan dalam diri kita. Gaji katakanlah gaji biasa* nine hundred. *Dia kata tak apalah kasi empat ratus tiga puluh cukuplah.* Little confidence. Put a little bit higher. Ok? There are cases where these are experience focus *lah.* Er: moving from one job to another, applying for new job. So there are cases where the panel asked what is the expected salary, 'I'm expecting sixty thousand', for example. So the next question they asked you is 'what can you give the company for sixty thousand?' To justify the sixty thousand. You justify, you get it. You got the job, you got the salary. So you *kena* weigh things up. Balance up. *Jangan terlalu rendah atau tinggi*. Ok, you have to state your expected salary. If you are already working, the current salary are drawing, *tengok ye* drawing. Let's say three thousand five hundred. I'm expecting a salary of four thousand two hundred. Minimum. So *kita* state *macam itulah*. Ok, gender, nothing much. Photo. (0.3) Photo. What would be written in the advertisement if they want you to send your photo? Any advertisement having the sentence for photo requirement? What is it stated?
Ss: Two latest passport size
T: Latest passport size (0.2) Two latest passport size photo. Any advertisement having the word 'n.r.'? 'n.r.' ada tak? Under photo. Three passport size photo, dia tulis 'n.r.'. No, ok. Anybody heard of that word? 'n.r.' (0.2) non-returnable. (0.2) *Photo itu tidak dikembalikan. Jangan hantar yang paling sayanglah. Susah nak ambil.* Update the latest *sikit-sikit, hantar.* If it's written non-returnable, normally even though it is not writtem, even it is on return but normally the photos are not returnable. Ok, any question? (0.3) Any question? Anything that you didn't understand so far. Simple lesson, checking your scollection of advertisement, and then try to distribute the requirement by advertisements, pull them together. At least they found ten, could be more, ok? And we have briefly looked at a few sentences in respond to the requirements. So that's what we've done. Question? No question? They don't pass around the paper for attendance. Maybe one for here, one for there. *Baru ambil* attendance, well, 2, 4, 6, 8, 10, 12, 14, 16, 18. All documentary seller. Silaturrahim. (0.5) All--if someone can write down another 11 names, which is easier. Ok, start passing around the paper. One group (35:00) *satu paper senang, cepat.* Ok, let me set the next task for you. Now I want each group to select one advertisement, among the three that you have collected, by only select one advertisement. Try and select the one that has more requirement, easy for you. Easy for you, more requirement. *Sebab banyak benda yang* you *kena* written. Try to produce

sentences in response ↑to the requirements. So we come back, *salin tak bersalin kita terus ye.* (   ) So, Tuesday. Next Tuesday *kalau macam ni I taknak pergi kelas.*
Ss: Next Tuesday is public holiday. Because the trip is until 30th
T: Ok, Until?
Ss: 30th night only come back. Ok, when I see you on the 9th, I want to hear from you, your requirements with the advertisement that you have selected plus your responses. And nearly after that, we will set the assignment task, cover letter, resume, and then the following week, we will do the mock interview. So that will bring you 10 + 10 + 10, 30 marks. 50 per cent. You want to in-charge? Ok ok. When I see you the next week you said will be the following Sunday, next week *takde. Boleh balik*. So, replcement I will discuss later. When I see you on Sunday, you will come back with selecting one advertisement per group (0.6) *tapi hari Raya Haji!*
Ss: Yes.
T: *Tutuplah semester ni tak ada dah.*
((Laughing))
T: Ok, whenever that I see you guys, ok. *Kalau perlu* replacement, *kita akan* replace. So I will see you having selected one advertisement, write out the requirements, provide responses for the requirements then we will discuss it. Immediately after that, you will work out your cover letter, resume and then mock interview. Ok. So, the next thing I want you to do is prepare the resume, prepare the cover letter, X Block which we will check, so that immediately after that I give you today, *dah siap dah*. Draft. Ok? Any last question? No. Thank you very much. That's all for today. (38:06)

Transcription of Interviews

Recording No.: 23
**Notes:*
A: Interviewer
B: Interviewee

A: Assalamualaikum [and good morning
B: [waalaikumussalam
A: Thank you for agreeing to talk to me. As you know my research is on code-switching
B: Yeah
A: And to see what is the perception about code-switching in your own practices in English classroom. Therefore, the content of the talk will be confidential and also only be used for my research purposes.
B: Ok
A: Ok, what can you understand about code-switching?
B: Code-switching is er: switching between two languages
A: Ehm:
B: Usually between first language or mother tongue and the second language.
A: Ehm:
B: In whatever area whatever place. So in the Malaysian case would be usually between Malay and English.

A: Ok, did you notice that you do any code-switching in the class just now?
B: I did.
A: Yeah. Quite a number of it.
B: Yes.
A: Was it a planned or an unplanned one?
B: No--no.
A: It wasn't a planned one.
B: It wasn't plan. Depending on how good-how students responded to certain things, deliver in English.
A: Ok.
B: So if I found that they were a bit blur about understanding that English part, then I: switched to Malay.
A: So the reason is to make sure that the students' understand, right?
B: Yes--yes
A: Apart from code-switching, is there any other strategies that you use in order for the students to understand?
B: Sometimes I do explain words and phrases and sentences in English itself.
A: Yeah
B: And then test them whether they understand or not. If they do then I just leave it without any code-switching. If they don't, then I still go back to code-switching.
A: Alright. So what would be your first and second languages then?
B: First language will be in English class will definitely be English. Second will be Malay.
A: Alright, good. Ok, so I'm sure you are experience in teaching English. How long would that be? How many years?
B: 34 years
A: 34 years, ok. So did you feel guilty or not of not using English all the time in the classroom?
B: No.
A: No. so it's ok for you--for you to use.
B: I feel sometimes, we have to use.
A: Yeah
B: Because I handle, throughout my career, I handle secondary school, and then *Maktab* and certain universities and so on. I fell that the foundation in English is not strong.
A: I see
B: So when they come up to us, no use completely speaking English throughout the lesson, and finally when you asked them did you understand, they'll say no, we don't understand. So defeat the purpose.
A: Sometimes they said they understand but they keep quiet, right?
B: They keep quiet.
((Laughing))
B: They dare not.
A: They dare not say they don't understand. So as a lecturer do you think you are proficient in English?
B: I am.
A: You are. So what would be your: er: sorry your strength as a lecturer?
B: As an English lecturer?
A: Yes, as English lecturer.
B: My strength is firstly my proficiency in English, Secondly, will be I do a lot of readings on self-development. So keep up with er: research and so on.

A: Ok.
B: So, that'the strength.
A: Do you have any weaknesses?
B: My weakness is, I dare not comply with certain principles and policies that government makes like English Class you must use English. So, I can't!
A: Ok.
B: I'm against it. Because as I said some students definitely need er: code-switching into Malay.
A: Ok, so how do you see the level of English language throughout the years, I mean you have been teaching quite years you see that it is became better the students' proficiency or is it just as normal as before?
B: I think worse I suppose.
A: Worse? Ok, why would you say that?
B: Because er: when they suddenly come up to university level *ah:*, some universities, most of universities, they say they must be proficient in English and so on. Minus the TESL and English subjects, we see that intake for universities and even polytechnic, English is not given a:=
A: =Priorty.
B; Priority. It's not a firm stamp on. You must pass English or score credit so on. So er: they are not bothered about you know, patching up all those problems in English. Students are not bothered about improving your English because they feel that even without English, they can move into many subjects. Many areas of study in Malaysia. So, I don't feel guilty because they come with little stock off English. So we can't deliver using everything in English. So, I stll believe that something is--something is strongly wrong with our policies, we are talking about English.
A: Alright, I think that's all.
B: Ok
A: Thank you for now. If you have anything else to add, you can contact me later.
B: Ok
A: Alright, thank you.
B: Thank you very much. (04:44)
