# Supplementary material for: Dataset from Code-switching between English and Malay Languages in Malaysian Premier Polytechnics ESL Classrooms
Source: Data Brief. 2022 Oct 29;45:108709. doi: 10.1016/j.dib.2022.108709 (PMC9679697; doi:10.1016/j.dib.2022.108709)
Supplement: Supplementary file 2 [file mmc2.pdf]

Students' Questionnaire

Title: Patterns and Beliefs of Lecturers' Code-Switching: An Inquiry into Malaysian Polytechnics English Language Classrooms

This questionnaire consists of three (3) sections. All information given will be kept confidential and will only be used for the sole purpose of this survey. Check your answers carefully and see if you have answered all the given questions. Please answer honestly and thank you in advance for your cooperation.

SECTION A: DEMOGRAPHIC PROFILE

Instruction: Please (√) tick the appropriate box(s) for each part.

1	Gender: Male ☐<br>Female ☐	
2	Race: Malay ☐<br>Chinese ☐<br>Indian ☐<br>Other ☐	
3	Age:	
4	SPM English results:	
5	Previous semester English course result:	
6	CGPA:	

SECTION B: LANGUAGE USE

7. Which language do you speak/hear most at home?

A. English B. Malay C. Cantonese D. Mandarin E. Tamil F. Other: _______

8. Which language does your parents consider most important for you?

____

9. In your family, who speak to you in English? _________________ How often?

A. Always B. Often C. Sometimes D. Never

10. How often do you listen to/watch programs in your native language (mother-tongue)?

A. Always B. Often C. Sometimes D. Never

11. How frequently do you use English language in your daily life?

1= Very Frequently 2 = Frequently 3 = Not Frequently 4 = Not Used At All

	1	2	3	4
a. Listen to radio station/s that use English.				
b. Watch movies or shows shown on television.				
c. Speak with your friends/family.				
d. Use internet to either email or do homework/assignments.				
e. Use word processor using programmes such as Word, Excel to do homework/ assignments.				
f. Presentation for classroom assignments.				
g. Read magazines/story books during your free time.				
h. Read books related to your homework/assignments.				
i. Write in the form of e.g. memoranda or reports etc.				
j. Ordering and buying food and drinks				

12. How satisfied are you with your ability to use English at Polytechnic? Tick (√) by responding to the scale of 1 to 4 as shown below:

1= Very Satisfied 2 = Satisfied 3 = Not Satisfied 4 = Not Satisfied At All

	1	2	3	4
a. Communicate orally with teachers and friends.				
b. Read and understand reference books related to assignments/homework.				
c. Presentation related to assignments/homework.				
d. Practice the language during practical lessons (ex: in the kitchen, housekeeping etc.)				
e. Write in the form of e.g. preparing reports and memoranda etc.				

13. It is common that students use both English and Malay Languages when they speak to friends, families or lecturers. Why do you think that you used both languages?

1= Strongly Agree 2 = Agree 3 = Disagree 4 = Strongly Disagree

	1	2	3	4
a. I know both English and Malay Languages very well.				
b. Just to show off that I know both English and Malay Languages.				
c. To show some western value/status in myself.				
d. To create closeness among my friends.				

e. To lower the language barrier that might have between myself and the others.				
f. To respect others who are not fluent in either languages.				
g. To cover up my weaknesses in English language.				
h. I use more English language in the Polytechnic.				
i. I use more Malay language at home.				
j. To transfer the intended meaning in order to avoid misunderstanding.				
k. To reinforce, emphasize or clarify messages that might not be understood.				
l. I use both languages equally either at the Polytechnic or at home.				

SECTION C: LEARNING PREFERENCES

14. Do you like your lecturer to teach in English or Malay language or both languages?

A. English language only B. Malay language only C. Both languages

Why?

____

____

15. Do your lecturers like to make jokes? A. Yes B. No

If yes, in what language does he/she always make jokes? ______________________

16. Can you understand your lecturers' jokes?

A. Always B. Often C. Sometimes D. Never

17. Are you satisfied with the lesson just now? A. Yes B. No

Why?

____

____

____

18. What improvement would you like your lecturer do in this particular subject?

____

____

____

____

____

- THANK YOU -

Lecturers' Questionnaire

Dear Colleague,

Thank you for your time and cooperation in answering this inventory on *'Patterns and Beliefs of Lecturers' Code-Switching: An Inquiry into Malaysian Polytechnics English Language Classrooms.'* The questionnaire is designed to identify the ways and reasons for lecturers to code-switch when they teach technical subjects using the English language. This study is carried out in fulfilling the requirement of completing my PhD. The survey can be completed in approximately 15 minutes or less. All the information gathered for the study will be kept completely confidential, and your identity and personal information will not be revealed. Your participation is greatly appreciated.

Yours Sincerely,

Mazlin Mohamed Mokhtar
Doctoral Researcher
Faculty of Education
University of Auckland
New Zealand

SECTION A: DEMOGRAPHIC PROFILE

Instruction: Please (√) tick the appropriate box(s) for each part.

1	Gender:	Male<br>Female	☐<br>☐
2	Race:	Malay<br>Chinese<br>Indian<br>Other	☐<br>☐<br>☐<br>☐
3	Age:		
4	Academic Qualification(s):		
5	Years of Teaching Experience:		
6	Course/Subject:		
7	Topic of lesson observed:		

SECTION B: LANGUAGE USE

8. Which language do you speak/hear most at home?

A. English B. Malay C. Cantonese D. Mandarin E. Tamil F. Other: _______

9. Which language do you hear most at your workplace?

A. English B. Malay C. Cantonese D. Mandarin E. Tamil F. Other: _______

10. How often do you listen to/watch programs in your native language (mother-tongue)?

A. Always B. Often C. Sometimes D. Never

11. How frequently do you use English language in your daily life?

1= Very Frequently 2 = Frequently 3 = Not Frequently 4 = Not Used At All

	1	2	3	4
a. Listen to radio station/s that use English.				
b. Watch movies or shows shown on television.				
c. Speak with my friends/family.				
d. Use internet to either email or do work.				
e. Use word processor using programmes such as Word, Excel to do work.				
f. Teaching in the classroom.				
g. Read magazines/story books during your free time.				
h. Read books related to my research/course.				
i. Write in the form of e.g. memoranda or reports etc.				
j. Ordering and buying food and drinks.				

12. How satisfied are you with your ability to use English at Polytechnic? Tick (√) by responding to the scale of 1 to 4 as shown below:

1= Very Satisfied 2 = Satisfied 3 = Not Satisfied 4 = Not Satisfied At All

	1	2	3	4
a. Communicate orally with colleagues and students.				
b. Read and understand reference books related to research/course.				
c. Teaching in the classroom.				
d. During meetings with superior or colleagues.				
e. Write in the form of e.g. preparing reports and memoranda etc.				

13. It is common that most people who are bilinguals (speak more than one language) use both English and Malay Languages when they speak to friends, families or colleagues. Why do you think that you used both languages?

1= Strongly Agree 2 = Agree 3 = Disagree 4 = Strongly Disagree

	1	2	3	4
a. I know both English and Malay Languages very well.				
b. Just to show off that I know both English and Malay Languages.				
c. To show some western value/status in myself.				
d. To create closeness among my colleagues and students.				
e. To signal the change in topic and to get students' attention.				
f. To build solidarity and intimate relations with the students.				
g. To lower the language barrier that might have between myself and the others.				
h. To transfer the necessary knowledge to the students for clarity and comprehension.				
i. To get students' attention and trust before proceeding to the teaching of concepts or theories, especially to those students who are weak.				
j. To cover up my weaknesses in English language.				
k. To transfer the intended meaning in order to avoid misunderstanding.				
l. To reinforce, emphasize or clarify messages that might not be understood.				
m. To respect others who are not fluent in either languages.				

n. I use more English language in the workplace.				
o. I use more Malay language at home.				
p. I use both languages equally either at the workplace or at home.				

SECTION C: TEACHING PREFERENCES

14. Do you like teaching in English or Malay language or both languages?

A. English language only B. Malay language only C. Both languages

Why?

____

____

15. Do you like to make jokes in the classroom? A. Yes B. No

If yes, in what language do you always make jokes?

____

Why?

____

____

____

16. Do you think your students will understand you better in English or Malay?

Why?

____

____

____

17. What do you do to ensure students' understanding if you are using English in your lesson?

____

____

18. Do you achieve your objective(s) in your lesson just now?

____

____

19. What improvement would you like to do in this particular subject?

____

____

____

____

____

- THANK YOU –
