# Supplementary material for: Dataset from Code-switching between English and Malay Languages in Malaysian Premier Polytechnics ESL Classrooms
Source: Data Brief. 2022 Oct 29;45:108709. doi: 10.1016/j.dib.2022.108709 (PMC9679697; doi:10.1016/j.dib.2022.108709)
Supplement: Supplementary file 5 [file mmc5.doc]

Frequencies

[DataSet1] C:\Users\Mazlin\Desktop\Students' survey.sav


Statistics	
		Polytechnic	How often?	
N	Valid	183	182	
	Missing	0	1	


Frequency Table


How often?	
		Frequency	Percent	Valid Percent	Cumulative Percent	
Valid	Always	5	2.7	2.7	2.7	
	Often	22	12.0	12.1	14.8	
	Sometimes	137	74.9	75.3	90.1	
	Never	18	9.8	9.9	100.0	
	Total	182	99.5	100.0		
Missing	Missing data	1	.5			
	Total	183	100.0			


[DataSet1] C:\Users\Mazlin\Desktop\Students' survey.sav


Case Processing Summary	
	Cases	
	Valid	Missing	Total	
	N	Percent	N	Percent	N	Percent	
Use English: Listen to radio station/s that use English * Polytechnic	183	100.0%	0	.0%	183	100.0%	
Use English: Listen to radio station/s that use English * Gender	183	100.0%	0	.0%	183	100.0%	
Watch movies or shows shown on tv * Polytechnic	183	100.0%	0	.0%	183	100.0%	
Watch movies or shows shown on tv * Gender	183	100.0%	0	.0%	183	100.0%	
Speak with your friends/family * Polytechnic	183	100.0%	0	.0%	183	100.0%	
Speak with your friends/family * Gender	183	100.0%	0	.0%	183	100.0%	
Use internet to email/do homework/assignments * Polytechnic	183	100.0%	0	.0%	183	100.0%	
Use internet to email/do homework/assignments * Gender	183	100.0%	0	.0%	183	100.0%	
Use Word/Excel to do homework/assignments * Polytechnic	183	100.0%	0	.0%	183	100.0%	
Use Word/Excel to do homework/assignments * Gender	183	100.0%	0	.0%	183	100.0%	
Presentation for classroom assignments * Polytechnic	183	100.0%	0	.0%	183	100.0%	
Presentation for classroom assignments * Gender	183	100.0%	0	.0%	183	100.0%	
Read magazines/story books during free time * Polytechnic	183	100.0%	0	.0%	183	100.0%	
Read magazines/story books during free time * Gender	183	100.0%	0	.0%	183	100.0%	
Read books related to homework/assignments * Polytechnic	182	99.5%	1	.5%	183	100.0%	
Read books related to homework/assignments * Gender	182	99.5%	1	.5%	183	100.0%	
Write memo, reports, etc. * Polytechnic	183	100.0%	0	.0%	183	100.0%	
Write memo, reports, etc. * Gender	183	100.0%	0	.0%	183	100.0%	
Ordering & buying food & drinks * Polytechnic	183	100.0%	0	.0%	183	100.0%	
Ordering & buying food & drinks * Gender	183	100.0%	0	.0%	183	100.0%	


Polytechnic	
		Frequency	Percent	Valid Percent	Cumulative Percent	
Valid	PUO	67	36.6	36.6	36.6	
	PSA	55	30.1	30.1	66.7	
	PIS	61	33.3	33.3	100.0	
	Total	183	100.0	100.0		


Use English: Listen to radio station/s that use English * Polytechnic Crosstabulation	
Count	
		Polytechnic		
		PUO	PSA	PIS	Total	
Use English: Listen to radio station/s that use English	Very frequently	14	9	11	34	
	Frequently	30	26	32	88	
	Not frequently	22	18	17	57	
	Not used at all	1	2	1	4	
	Total	67	55	61	183	


Use English: Listen to radio station/s that use English * Gender Crosstabulation	
Count	
		Gender		
		Male	Female	Total	
Use English: Listen to radio station/s that use English	Very frequently	20	14	34	
	Frequently	48	40	88	
	Not frequently	28	29	57	
	Not used at all	2	2	4	
	Total	98	85	183	


Watch movies or shows shown on tv * Polytechnic Crosstabulation	
Count	
		Polytechnic		
		PUO	PSA	PIS	Total	
Watch movies or shows shown on tv	Very frequently	30	17	27	74	
	Frequently	36	30	33	99	
	Not frequently	1	8	1	10	
	Total	67	55	61	183	


Watch movies or shows shown on tv * Gender Crosstabulation	
Count	
		Gender		
		Male	Female	Total	
Watch movies or shows shown on tv	Very frequently	42	32	74	
	Frequently	51	48	99	
	Not frequently	5	5	10	
	Total	98	85	183	


Speak with your friends/family * Polytechnic Crosstabulation	
Count	
		Polytechnic		
		PUO	PSA	PIS	Total	
Speak with your friends/family	Very frequently	1	0	2	3	
	Frequently	20	20	17	57	
	Not frequently	46	30	37	113	
	Not used at all	0	5	5	10	
	Total	67	55	61	183	


Speak with your friends/family * Gender Crosstabulation	
Count	
		Gender		
		Male	Female	Total	
Speak with your friends/family	Very frequently	1	2	3	
	Frequently	29	28	57	
	Not frequently	61	52	113	
	Not used at all	7	3	10	
	Total	98	85	183	


Use internet to email/do homework/assignments * Polytechnic Crosstabulation	
Count	
		Polytechnic		
		PUO	PSA	PIS	Total	
Use internet to email/do homework/assignments	Very frequently	36	19	39	94	
	Frequently	31	27	18	76	
	Not frequently	0	8	3	11	
	Not used at all	0	1	1	2	
	Total	67	55	61	183	


Use internet to email/do homework/assignments * Gender Crosstabulation	
Count	
		Gender		
		Male	Female	Total	
Use internet to email/do homework/assignments	Very frequently	51	43	94	
	Frequently	41	35	76	
	Not frequently	5	6	11	
	Not used at all	1	1	2	
	Total	98	85	183	


Use Word/Excel to do homework/assignments * Polytechnic Crosstabulation	
Count	
		Polytechnic		
		PUO	PSA	PIS	Total	
Use Word/Excel to do homework/assignments	Very frequently	43	21	36	100	
	Frequently	22	25	23	70	
	Not frequently	2	8	1	11	
	Not used at all	0	1	1	2	
	Total	67	55	61	183	


Use Word/Excel to do homework/assignments * Gender Crosstabulation	
Count	
		Gender		
		Male	Female	Total	
Use Word/Excel to do homework/assignments	Very frequently	53	47	100	
	Frequently	39	31	70	
	Not frequently	4	7	11	
	Not used at all	2	0	2	
	Total	98	85	183	


Presentation for classroom assignments * Polytechnic Crosstabulation	
Count	
		Polytechnic		
		PUO	PSA	PIS	Total	
Presentation for classroom assignments	Very frequently	35	13	20	68	
	Frequently	30	30	30	90	
	Not frequently	2	12	9	23	
	Not used at all	0	0	2	2	
	Total	67	55	61	183	


Presentation for classroom assignments * Gender Crosstabulation	
Count	
		Gender		
		Male	Female	Total	
Presentation for classroom assignments	Very frequently	40	28	68	
	Frequently	49	41	90	
	Not frequently	8	15	23	
	Not used at all	1	1	2	
	Total	98	85	183	


Read magazines/story books during free time * Polytechnic Crosstabulation	
Count	
		Polytechnic		
		PUO	PSA	PIS	Total	
Read magazines/story books during free time	Very frequently	3	4	7	14	
	Frequently	24	21	23	68	
	Not frequently	35	25	25	85	
	Not used at all	5	4	6	15	
	13.00	0	1	0	1	
	Total	67	55	61	183	


Read magazines/story books during free time * Gender Crosstabulation	
Count	
		Gender		
		Male	Female	Total	
Read magazines/story books during free time	Very frequently	5	9	14	
	Frequently	34	34	68	
	Not frequently	48	37	85	
	Not used at all	10	5	15	
	13.00	1	0	1	
	Total	98	85	183	


Read books related to homework/assignments * Polytechnic Crosstabulation	
Count	
		Polytechnic		
		PUO	PSA	PIS	Total	
Read books related to homework/assignments	Very frequently	13	8	7	28	
	Frequently	38	33	31	102	
	Not frequently	14	10	19	43	
	Not used at all	2	4	3	9	
	Total	67	55	60	182	


Read books related to homework/assignments * Gender Crosstabulation	
Count	
		Gender		
		Male	Female	Total	
Read books related to homework/assignments	Very frequently	16	12	28	
	Frequently	55	47	102	
	Not frequently	19	24	43	
	Not used at all	7	2	9	
	Total	97	85	182	


Write memo, reports, etc. * Polytechnic Crosstabulation	
Count	
		Polytechnic		
		PUO	PSA	PIS	Total	
Write memo, reports, etc.	Very frequently	13	3	12	28	
	Frequently	28	23	29	80	
	Not frequently	22	24	19	65	
	Not used at all	4	5	1	10	
	Total	67	55	61	183	


Write memo, reports, etc. * Gender Crosstabulation	
Count	
		Gender		
		Male	Female	Total	
Write memo, reports, etc.	Very frequently	16	12	28	
	Frequently	42	38	80	
	Not frequently	33	32	65	
	Not used at all	7	3	10	
	Total	98	85	183	


Ordering & buying food & drinks * Polytechnic Crosstabulation	
Count	
		Polytechnic		
		PUO	PSA	PIS	Total	
Ordering & buying food & drinks	Very frequently	4	5	4	13	
	Frequently	17	17	15	49	
	Not frequently	40	26	33	99	
	Not used at all	6	7	9	22	
	Total	67	55	61	183	


Ordering & buying food & drinks * Gender Crosstabulation	
Count	
		Gender		
		Male	Female	Total	
Ordering & buying food & drinks	Very frequently	7	6	13	
	Frequently	26	23	49	
	Not frequently	52	47	99	
	Not used at all	13	9	22	
	Total	98	85	183	


CROSSTABS   /TABLES=C5 BY Polytechnic Gender   /FORMAT=AVALUE TABLES   /CELLS=COUNT   /COUNT ROUND CELL.


[DataSet1] C:\Users\Mazlin\Desktop\Students' survey.sav


Case Processing Summary	
	Cases	
	Valid	Missing	Total	
	N	Percent	N	Percent	N	Percent	
What improvement? * Polytechnic	144	78.7%	39	21.3%	183	100.0%	
What improvement? * Gender	144	78.7%	39	21.3%	183	100.0%	


What improvement? * Polytechnic Crosstabulation	
Count	
		Polytechnic		
		PUO	PSA	PIS	Total	
What improvement?	Teach more on English languages	1	0	0	1	
	Use more methods in teaching and to keep our interest of English	1	0	0	1	
	Make more jokes	9	3	4	16	
	More classroom activities/games to attract Ss' interests/to improve English	4	10	9	23	
	Learn outside the classroom and mix with other classes	1	0	1	2	
	Use more LCD	1	0	0	1	
	Not too serious/be funny/friendly	3	2	2	7	
	Help students more	1	1	0	2	
	Use Malay if Ss' don't understand	1	0	0	1	
	More sporting & cheerful	1	0	0	1	
	Lecturer gives more information/explanations	2	0	2	4	
	More group discussions	3	0	0	3	
	More presentations/role plays to give more confident	3	1	2	6	
	Make the topic more interesting	2	0	0	2	
	No improvement needed	8	4	5	17	
	Give more assessments/assignments	3	1	0	4	
	Use both languages for better understanding	1	2	4	7	
	Always give new word in English	1	0	1	2	
	Use more video	1	1	1	3	
	Lecturers gives more chance to speak in English/to improve	6	4	5	15	
	Lecturer to use more English (in other situation too)/English only	3	1	4	8	
	More visual aids	1	0	1	2	
	Teach to communicate with foreigners	1	0	1	2	
	Extra classes for English	1	0	0	1	
	More creative	0	1	0	1	
	More active	0	1	0	1	
	Give more explanation	0	1	0	1	
	Other	0	1	1	2	
	Use simple languages	0	0	2	2	
	Improve communication skills between lecturer and students	0	0	1	1	
	Add more interesting topics	0	0	1	1	
	Write important things on the board	0	0	1	1	
	Closer to students	0	0	1	1	
	Ask more questions to students	0	0	1	1	
	Less teacher-talk	0	0	1	1	
	Total	59	34	51	144	


What improvement? * Gender Crosstabulation	
Count	
		Gender		
		Male	Female	Total	
What improvement?	Teach more on English languages	1	0	1	
	Use more methods in teaching and to keep our interest of English	1	0	1	
	Make more jokes	13	3	16	
	More classroom activities/games to attract Ss' interests/to improve English	9	14	23	
	Learn outside the classroom and mix with other classes	2	0	2	
	Use more LCD	1	0	1	
	Not too serious/be funny/friendly	5	2	7	
	Help students more	2	0	2	
	Use Malay if Ss' don't understand	1	0	1	
	More sporting & cheerful	1	0	1	
	Lecturer gives more information/explanations	2	2	4	
	More group discussions	3	0	3	
	More presentations/role plays to give more confident	2	4	6	
	Make the topic more interesting	1	1	2	
	No improvement needed	13	4	17	
	Give more assessments/assignments	1	3	4	
	Use both languages for better understanding	4	3	7	
	Always give new word in English	1	1	2	
	Use more video	2	1	3	
	Lecturers gives more chance to speak in English/to improve	4	11	15	
	Lecturer to use more English (in other situation too)/English only	5	3	8	
	More visual aids	1	1	2	
	Teach to communicate with foreigners	1	1	2	
	Extra classes for English	0	1	1	
	More creative	1	0	1	
	More active	1	0	1	
	Give more explanation	1	0	1	
	Other	2	0	2	
	Use simple languages	1	1	2	
	Improve communication skills between lecturer and students	1	0	1	
	Add more interesting topics	0	1	1	
	Write important things on the board	0	1	1	
	Closer to students	1	0	1	
	Ask more questions to students	0	1	1	
	Less teacher-talk	0	1	1	
	Total	84	60	144	
