# Supplementary material for: Dataset from Code-switching between English and Malay Languages in Malaysian Premier Polytechnics ESL Classrooms
Source: Data Brief. 2022 Oct 29;45:108709. doi: 10.1016/j.dib.2022.108709 (PMC9679697; doi:10.1016/j.dib.2022.108709)
Supplement: Supplementary file 6 [file mmc6.doc]

[DataSet1] C:\Users\Mazlin\Desktop\Lecturers' survey.sav


Case Processing Summary	
	Cases	
	Valid	Missing	Total	
	N	Percent	N	Percent	N	Percent	
Polytechnic * Use English: Listen to radio station/s that use English	9	100.0%	0	.0%	9	100.0%	
Polytechnic * Watch movies or shows shown on tv	9	100.0%	0	.0%	9	100.0%	
Polytechnic * Speak with your friends/family	9	100.0%	0	.0%	9	100.0%	
Polytechnic * Use internet to email/do work	9	100.0%	0	.0%	9	100.0%	
Polytechnic * Use Word/Excel to do work	9	100.0%	0	.0%	9	100.0%	
Polytechnic * Teaching in the classroom	9	100.0%	0	.0%	9	100.0%	
Polytechnic * Read magazines/story books during free time	9	100.0%	0	.0%	9	100.0%	
Polytechnic * Read books related to my research/course	9	100.0%	0	.0%	9	100.0%	
Polytechnic * Write memo, reports, etc.	9	100.0%	0	.0%	9	100.0%	
Polytechnic * Ordering & buying food & drinks	9	100.0%	0	.0%	9	100.0%	
Gender * Use English: Listen to radio station/s that use English	9	100.0%	0	.0%	9	100.0%	
Gender * Watch movies or shows shown on tv	9	100.0%	0	.0%	9	100.0%	
Gender * Speak with your friends/family	9	100.0%	0	.0%	9	100.0%	
Gender * Use internet to email/do work	9	100.0%	0	.0%	9	100.0%	
Gender * Use Word/Excel to do work	9	100.0%	0	.0%	9	100.0%	
Gender * Teaching in the classroom	9	100.0%	0	.0%	9	100.0%	
Gender * Read magazines/story books during free time	9	100.0%	0	.0%	9	100.0%	
Gender * Read books related to my research/course	9	100.0%	0	.0%	9	100.0%	
Gender * Write memo, reports, etc.	9	100.0%	0	.0%	9	100.0%	
Gender * Ordering & buying food & drinks	9	100.0%	0	.0%	9	100.0%	
Teaching experience * Use English: Listen to radio station/s that use English	9	100.0%	0	.0%	9	100.0%	
Teaching experience * Watch movies or shows shown on tv	9	100.0%	0	.0%	9	100.0%	
Teaching experience * Speak with your friends/family	9	100.0%	0	.0%	9	100.0%	
Teaching experience * Use internet to email/do work	9	100.0%	0	.0%	9	100.0%	
Teaching experience * Use Word/Excel to do work	9	100.0%	0	.0%	9	100.0%	
Teaching experience * Teaching in the classroom	9	100.0%	0	.0%	9	100.0%	
Teaching experience * Read magazines/story books during free time	9	100.0%	0	.0%	9	100.0%	
Teaching experience * Read books related to my research/course	9	100.0%	0	.0%	9	100.0%	
Teaching experience * Write memo, reports, etc.	9	100.0%	0	.0%	9	100.0%	
Teaching experience * Ordering & buying food & drinks	9	100.0%	0	.0%	9	100.0%	


Polytechnic * Use English: Listen to radio station/s that use English Crosstabulation	
Count	
		Use English: Listen to radio station/s that use English		
		Very frequently	Frequently	Not frequently	Total	
Polytechnic	PUO	3	0	0	3	
	PSA	0	2	0	2	
	PIS	2	1	1	4	
	Total	5	3	1	9	


Polytechnic * Watch movies or shows shown on tv Crosstabulation	
Count	
		Watch movies or shows shown on tv		
		Very frequently	Frequently	Not frequently	Total	
Polytechnic	PUO	2	1	0	3	
	PSA	1	1	0	2	
	PIS	2	1	1	4	
	Total	5	3	1	9	


Polytechnic * Speak with your friends/family Crosstabulation	
Count	
		Speak with your friends/family		
		Very frequently	Frequently	Not frequently	Total	
Polytechnic	PUO	1	1	1	3	
	PSA	0	1	1	2	
	PIS	0	2	2	4	
	Total	1	4	4	9	


Polytechnic * Use internet to email/do work Crosstabulation	
Count	
		Use internet to email/do work		
		Very frequently	Frequently	Total	
Polytechnic	PUO	3	0	3	
	PSA	2	0	2	
	PIS	3	1	4	
	Total	8	1	9	


Polytechnic * Use Word/Excel to do work Crosstabulation	
Count	
		Use Word/Excel to do work		
		Very frequently	Frequently	Total	
Polytechnic	PUO	3	0	3	
	PSA	1	1	2	
	PIS	3	1	4	
	Total	7	2	9	


Polytechnic * Teaching in the classroom Crosstabulation	
Count	
		Teaching in the classroom		
		Very frequently	Frequently	Total	
Polytechnic	PUO	3	0	3	
	PSA	1	1	2	
	PIS	2	2	4	
	Total	6	3	9	


Polytechnic * Read magazines/story books during free time Crosstabulation	
Count	
		Read magazines/story books during free time		
		Very frequently	Frequently	Not frequently	Total	
Polytechnic	PUO	3	0	0	3	
	PSA	1	0	1	2	
	PIS	2	1	1	4	
	Total	6	1	2	9	


Polytechnic * Read books related to my research/course Crosstabulation	
Count	
		Read books related to my research/course		
		Very frequently	Frequently	Not frequently	Total	
Polytechnic	PUO	3	0	0	3	
	PSA	1	0	1	2	
	PIS	1	3	0	4	
	Total	5	3	1	9	


Polytechnic * Write memo, reports, etc. Crosstabulation	
Count	
		Write memo, reports, etc.		
		Very frequently	Frequently	Total	
Polytechnic	PUO	3	0	3	
	PSA	1	1	2	
	PIS	2	2	4	
	Total	6	3	9	


Polytechnic * Ordering & buying food & drinks Crosstabulation	
Count	
		Ordering & buying food & drinks		
		Frequently	Not frequently	Total	
Polytechnic	PUO	2	1	3	
	PSA	1	1	2	
	PIS	3	1	4	
	Total	6	3	9	


Gender * Use English: Listen to radio station/s that use English Crosstabulation	
Count	
		Use English: Listen to radio station/s that use English		
		Very frequently	Frequently	Not frequently	Total	
Gender	Male	1	2	0	3	
	Female	4	1	1	6	
	Total	5	3	1	9	


Gender * Watch movies or shows shown on tv Crosstabulation	
Count	
		Watch movies or shows shown on tv		
		Very frequently	Frequently	Not frequently	Total	
Gender	Male	1	2	0	3	
	Female	4	1	1	6	
	Total	5	3	1	9	


Gender * Speak with your friends/family Crosstabulation	
Count	
		Speak with your friends/family		
		Very frequently	Frequently	Not frequently	Total	
Gender	Male	0	0	3	3	
	Female	1	4	1	6	
	Total	1	4	4	9	


Gender * Use internet to email/do work Crosstabulation	
Count	
		Use internet to email/do work		
		Very frequently	Frequently	Total	
Gender	Male	2	1	3	
	Female	6	0	6	
	Total	8	1	9	


Gender * Use Word/Excel to do work Crosstabulation	
Count	
		Use Word/Excel to do work		
		Very frequently	Frequently	Total	
Gender	Male	1	2	3	
	Female	6	0	6	
	Total	7	2	9	


Gender * Teaching in the classroom Crosstabulation	
Count	
		Teaching in the classroom		
		Very frequently	Frequently	Total	
Gender	Male	0	3	3	
	Female	6	0	6	
	Total	6	3	9	


Gender * Read magazines/story books during free time Crosstabulation	
Count	
		Read magazines/story books during free time		
		Very frequently	Frequently	Not frequently	Total	
Gender	Male	1	1	1	3	
	Female	5	0	1	6	
	Total	6	1	2	9	


Gender * Read books related to my research/course Crosstabulation	
Count	
		Read books related to my research/course		
		Very frequently	Frequently	Not frequently	Total	
Gender	Male	0	2	1	3	
	Female	5	1	0	6	
	Total	5	3	1	9	


Gender * Write memo, reports, etc. Crosstabulation	
Count	
		Write memo, reports, etc.		
		Very frequently	Frequently	Total	
Gender	Male	0	3	3	
	Female	6	0	6	
	Total	6	3	9	


Gender * Ordering & buying food & drinks Crosstabulation	
Count	
		Ordering & buying food & drinks		
		Frequently	Not frequently	Total	
Gender	Male	1	2	3	
	Female	5	1	6	
	Total	6	3	9	


Teaching experience * Use English: Listen to radio station/s that use English Crosstabulation	
Count	
		Use English: Listen to radio station/s that use English		
		Very frequently	Frequently	Not frequently	Total	
Teaching experience	0-5	3	0	1	4	
	6-10	0	1	0	1	
	11-15	1	0	0	1	
	21-25	0	1	0	1	
	26-30	1	0	0	1	
	31-35	0	1	0	1	
	Total	5	3	1	9	


Teaching experience * Watch movies or shows shown on tv Crosstabulation	
Count	
		Watch movies or shows shown on tv		
		Very frequently	Frequently	Not frequently	Total	
Teaching experience	0-5	3	0	1	4	
	6-10	1	0	0	1	
	11-15	0	1	0	1	
	21-25	0	1	0	1	
	26-30	1	0	0	1	
	31-35	0	1	0	1	
	Total	5	3	1	9	


Teaching experience * Speak with your friends/family Crosstabulation	
Count	
		Speak with your friends/family		
		Very frequently	Frequently	Not frequently	Total	
Teaching experience	0-5	0	2	2	4	
	6-10	0	1	0	1	
	11-15	0	1	0	1	
	21-25	0	0	1	1	
	26-30	1	0	0	1	
	31-35	0	0	1	1	
	Total	1	4	4	9	


Teaching experience * Use internet to email/do work Crosstabulation	
Count	
		Use internet to email/do work		
		Very frequently	Frequently	Total	
Teaching experience	0-5	4	0	4	
	6-10	1	0	1	
	11-15	1	0	1	
	21-25	1	0	1	
	26-30	1	0	1	
	31-35	0	1	1	
	Total	8	1	9	


Teaching experience * Use Word/Excel to do work Crosstabulation	
Count	
		Use Word/Excel to do work		
		Very frequently	Frequently	Total	
Teaching experience	0-5	4	0	4	
	6-10	1	0	1	
	11-15	1	0	1	
	21-25	0	1	1	
	26-30	1	0	1	
	31-35	0	1	1	
	Total	7	2	9	


Teaching experience * Teaching in the classroom Crosstabulation	
Count	
		Teaching in the classroom		
		Very frequently	Frequently	Total	
Teaching experience	0-5	3	1	4	
	6-10	1	0	1	
	11-15	1	0	1	
	21-25	0	1	1	
	26-30	1	0	1	
	31-35	0	1	1	
	Total	6	3	9	


Teaching experience * Read magazines/story books during free time Crosstabulation	
Count	
		Read magazines/story books during free time		
		Very frequently	Frequently	Not frequently	Total	
Teaching experience	0-5	3	0	1	4	
	6-10	1	0	0	1	
	11-15	1	0	0	1	
	21-25	0	0	1	1	
	26-30	1	0	0	1	
	31-35	0	1	0	1	
	Total	6	1	2	9	


Teaching experience * Read books related to my research/course Crosstabulation	
Count	
		Read books related to my research/course		
		Very frequently	Frequently	Not frequently	Total	
Teaching experience	0-5	2	2	0	4	
	6-10	1	0	0	1	
	11-15	1	0	0	1	
	21-25	0	0	1	1	
	26-30	1	0	0	1	
	31-35	0	1	0	1	
	Total	5	3	1	9	


Teaching experience * Write memo, reports, etc. Crosstabulation	
Count	
		Write memo, reports, etc.		
		Very frequently	Frequently	Total	
Teaching experience	0-5	3	1	4	
	6-10	1	0	1	
	11-15	1	0	1	
	21-25	0	1	1	
	26-30	1	0	1	
	31-35	0	1	1	
	Total	6	3	9	


Teaching experience * Ordering & buying food & drinks Crosstabulation	
Count	
		Ordering & buying food & drinks		
		Frequently	Not frequently	Total	
Teaching experience	0-5	3	1	4	
	6-10	1	0	1	
	11-15	1	0	1	
	21-25	0	1	1	
	26-30	1	0	1	
	31-35	0	1	1	
	Total	6	3	9	


CROSSTABS   /TABLES=Polytechnic Gender Years BY B5a B5b B5c B5d B5e   /FORMAT=AVALUE TABLES   /CELLS=COUNT   /COUNT ROUND CELL.


[DataSet1] C:\Users\Mazlin\Desktop\Lecturers' survey.sav


Case Processing Summary	
	Cases	
	Valid	Missing	Total	
	N	Percent	N	Percent	N	Percent	
Polytechnic * Satisfied with ability to use English: Communicate orally with colleagues & Ss	9	100.0%	0	.0%	9	100.0%	
Polytechnic * Read & understand reference books related to research/course	9	100.0%	0	.0%	9	100.0%	
Polytechnic * Teaching in the classroom	9	100.0%	0	.0%	9	100.0%	
Polytechnic * During meetings with superior or colleagues	9	100.0%	0	.0%	9	100.0%	
Polytechnic * Write in the form e.g. reports, memo, etc.	9	100.0%	0	.0%	9	100.0%	
Gender * Satisfied with ability to use English: Communicate orally with colleagues & Ss	9	100.0%	0	.0%	9	100.0%	
Gender * Read & understand reference books related to research/course	9	100.0%	0	.0%	9	100.0%	
Gender * Teaching in the classroom	9	100.0%	0	.0%	9	100.0%	
Gender * During meetings with superior or colleagues	9	100.0%	0	.0%	9	100.0%	
Gender * Write in the form e.g. reports, memo, etc.	9	100.0%	0	.0%	9	100.0%	
Teaching experience * Satisfied with ability to use English: Communicate orally with colleagues & Ss	9	100.0%	0	.0%	9	100.0%	
Teaching experience * Read & understand reference books related to research/course	9	100.0%	0	.0%	9	100.0%	
Teaching experience * Teaching in the classroom	9	100.0%	0	.0%	9	100.0%	
Teaching experience * During meetings with superior or colleagues	9	100.0%	0	.0%	9	100.0%	
Teaching experience * Write in the form e.g. reports, memo, etc.	9	100.0%	0	.0%	9	100.0%	


Polytechnic * Satisfied with ability to use English: Communicate orally with colleagues & Ss Crosstabulation	
Count	
		Satisfied with ability to use English: Communicate orally with colleagues & Ss		
		Very satisfied	Satisfied	Not satisfied	Total	
Polytechnic	PUO	3	0	0	3	
	PSA	1	1	0	2	
	PIS	0	3	1	4	
	Total	4	4	1	9	


Polytechnic * Read & understand reference books related to research/course Crosstabulation	
Count	
		Read & understand reference books related to research/course		
		Very satisfied	Satisfied	Not satisfied	Total	
Polytechnic	PUO	3	0	0	3	
	PSA	2	0	0	2	
	PIS	2	1	1	4	
	Total	7	1	1	9	


Polytechnic * Teaching in the classroom Crosstabulation	
Count	
		Teaching in the classroom		
		Very satisfied	Satisfied	Total	
Polytechnic	PUO	3	0	3	
	PSA	2	0	2	
	PIS	1	3	4	
	Total	6	3	9	


Polytechnic * During meetings with superior or colleagues Crosstabulation	
Count	
		During meetings with superior or colleagues		
		Very satisfied	Satisfied	Not satisfied	Total	
Polytechnic	PUO	3	0	0	3	
	PSA	0	2	0	2	
	PIS	1	2	1	4	
	Total	4	4	1	9	


Polytechnic * Write in the form e.g. reports, memo, etc. Crosstabulation	
Count	
		Write in the form e.g. reports, memo, etc.		
		Very satisfied	Satisfied	Total	
Polytechnic	PUO	2	1	3	
	PSA	1	1	2	
	PIS	1	3	4	
	Total	4	5	9	


Gender * Satisfied with ability to use English: Communicate orally with colleagues & Ss Crosstabulation	
Count	
		Satisfied with ability to use English: Communicate orally with colleagues & Ss		
		Very satisfied	Satisfied	Not satisfied	Total	
Gender	Male	1	2	0	3	
	Female	3	2	1	6	
	Total	4	4	1	9	


Gender * Read & understand reference books related to research/course Crosstabulation	
Count	
		Read & understand reference books related to research/course		
		Very satisfied	Satisfied	Not satisfied	Total	
Gender	Male	2	1	0	3	
	Female	5	0	1	6	
	Total	7	1	1	9	


Gender * Teaching in the classroom Crosstabulation	
Count	
		Teaching in the classroom		
		Very satisfied	Satisfied	Total	
Gender	Male	2	1	3	
	Female	4	2	6	
	Total	6	3	9	


Gender * During meetings with superior or colleagues Crosstabulation	
Count	
		During meetings with superior or colleagues		
		Very satisfied	Satisfied	Not satisfied	Total	
Gender	Male	0	3	0	3	
	Female	4	1	1	6	
	Total	4	4	1	9	


Gender * Write in the form e.g. reports, memo, etc. Crosstabulation	
Count	
		Write in the form e.g. reports, memo, etc.		
		Very satisfied	Satisfied	Total	
Gender	Male	0	3	3	
	Female	4	2	6	
	Total	4	5	9	


Teaching experience * Satisfied with ability to use English: Communicate orally with colleagues & Ss Crosstabulation	
Count	
		Satisfied with ability to use English: Communicate orally with colleagues & Ss		
		Very satisfied	Satisfied	Not satisfied	Total	
Teaching experience	0-5	1	2	1	4	
	6-10	0	1	0	1	
	11-15	1	0	0	1	
	21-25	1	0	0	1	
	26-30	1	0	0	1	
	31-35	0	1	0	1	
	Total	4	4	1	9	


Teaching experience * Read & understand reference books related to research/course Crosstabulation	
Count	
		Read & understand reference books related to research/course		
		Very satisfied	Satisfied	Not satisfied	Total	
Teaching experience	0-5	3	0	1	4	
	6-10	1	0	0	1	
	11-15	1	0	0	1	
	21-25	1	0	0	1	
	26-30	1	0	0	1	
	31-35	0	1	0	1	
	Total	7	1	1	9	


Teaching experience * Teaching in the classroom Crosstabulation	
Count	
		Teaching in the classroom		
		Very satisfied	Satisfied	Total	
Teaching experience	0-5	1	3	4	
	6-10	1	0	1	
	11-15	1	0	1	
	21-25	1	0	1	
	26-30	1	0	1	
	31-35	1	0	1	
	Total	6	3	9	


Teaching experience * During meetings with superior or colleagues Crosstabulation	
Count	
		During meetings with superior or colleagues		
		Very satisfied	Satisfied	Not satisfied	Total	
Teaching experience	0-5	2	1	1	4	
	6-10	0	1	0	1	
	11-15	1	0	0	1	
	21-25	0	1	0	1	
	26-30	1	0	0	1	
	31-35	0	1	0	1	
	Total	4	4	1	9	


Teaching experience * Write in the form e.g. reports, memo, etc. Crosstabulation	
Count	
		Write in the form e.g. reports, memo, etc.		
		Very satisfied	Satisfied	Total	
Teaching experience	0-5	1	3	4	
	6-10	1	0	1	
	11-15	1	0	1	
	21-25	0	1	1	
	26-30	1	0	1	
	31-35	0	1	1	
	Total	4	5	9	


[DataSet1] C:\Users\Mazlin\Desktop\Lecturers' survey.sav


Case Processing Summary	
	Cases	
	Valid	Missing	Total	
	N	Percent	N	Percent	N	Percent	
Polytechnic * Why used both languages: I know both languages very well	9	100.0%	0	.0%	9	100.0%	
Polytechnic * Just to show off that I know both languages	9	100.0%	0	.0%	9	100.0%	
Polytechnic * To show some western value/status in myself	9	100.0%	0	.0%	9	100.0%	
Polytechnic * To create closeness among my colleagues and students	9	100.0%	0	.0%	9	100.0%	
Polytechnic * To signal the change in topic and to get Ss' attention	9	100.0%	0	.0%	9	100.0%	
Polytechnic * To build solidarity and intimate relations with the students	8	88.9%	1	11.1%	9	100.0%	
Polytechnic * To lower the language barrier that might have between myself and the others	9	100.0%	0	.0%	9	100.0%	
Polytechnic * To transfer the necessary knowledge to the students for clarity & comprehension	9	100.0%	0	.0%	9	100.0%	
Polytechnic * To get Ss' attention & trust before proceeding to the teaching of concepts/theories esp. those who are weak	9	100.0%	0	.0%	9	100.0%	
Polytechnic * To cover up my weaknesses in English language	9	100.0%	0	.0%	9	100.0%	
Polytechnic * To transfer the intended meaning in order to avoid misunderstanding	9	100.0%	0	.0%	9	100.0%	
Polytechnic * To reinforce, emphasize or clarify messages that might not be understood	9	100.0%	0	.0%	9	100.0%	
Polytechnic * To respect others who are not fluent in either languages	9	100.0%	0	.0%	9	100.0%	
Polytechnic * I use more English in the workplace	9	100.0%	0	.0%	9	100.0%	
Polytechnic * I use more Malay language at home	9	100.0%	0	.0%	9	100.0%	
Polytechnic * I used both languages equally at the workplace and home	9	100.0%	0	.0%	9	100.0%	
Gender * Why used both languages: I know both languages very well	9	100.0%	0	.0%	9	100.0%	
Gender * Just to show off that I know both languages	9	100.0%	0	.0%	9	100.0%	
Gender * To show some western value/status in myself	9	100.0%	0	.0%	9	100.0%	
Gender * To create closeness among my colleagues and students	9	100.0%	0	.0%	9	100.0%	
Gender * To signal the change in topic and to get Ss' attention	9	100.0%	0	.0%	9	100.0%	
Gender * To build solidarity and intimate relations with the students	8	88.9%	1	11.1%	9	100.0%	
Gender * To lower the language barrier that might have between myself and the others	9	100.0%	0	.0%	9	100.0%	
Gender * To transfer the necessary knowledge to the students for clarity & comprehension	9	100.0%	0	.0%	9	100.0%	
Gender * To get Ss' attention & trust before proceeding to the teaching of concepts/theories esp. those who are weak	9	100.0%	0	.0%	9	100.0%	
Gender * To cover up my weaknesses in English language	9	100.0%	0	.0%	9	100.0%	
Gender * To transfer the intended meaning in order to avoid misunderstanding	9	100.0%	0	.0%	9	100.0%	
Gender * To reinforce, emphasize or clarify messages that might not be understood	9	100.0%	0	.0%	9	100.0%	
Gender * To respect others who are not fluent in either languages	9	100.0%	0	.0%	9	100.0%	
Gender * I use more English in the workplace	9	100.0%	0	.0%	9	100.0%	
Gender * I use more Malay language at home	9	100.0%	0	.0%	9	100.0%	
Gender * I used both languages equally at the workplace and home	9	100.0%	0	.0%	9	100.0%	
Teaching experience * Why used both languages: I know both languages very well	9	100.0%	0	.0%	9	100.0%	
Teaching experience * Just to show off that I know both languages	9	100.0%	0	.0%	9	100.0%	
Teaching experience * To show some western value/status in myself	9	100.0%	0	.0%	9	100.0%	
Teaching experience * To create closeness among my colleagues and students	9	100.0%	0	.0%	9	100.0%	
Teaching experience * To signal the change in topic and to get Ss' attention	9	100.0%	0	.0%	9	100.0%	
Teaching experience * To build solidarity and intimate relations with the students	8	88.9%	1	11.1%	9	100.0%	
Teaching experience * To lower the language barrier that might have between myself and the others	9	100.0%	0	.0%	9	100.0%	
Teaching experience * To transfer the necessary knowledge to the students for clarity & comprehension	9	100.0%	0	.0%	9	100.0%	
Teaching experience * To get Ss' attention & trust before proceeding to the teaching of concepts/theories esp. those who are weak	9	100.0%	0	.0%	9	100.0%	
Teaching experience * To cover up my weaknesses in English language	9	100.0%	0	.0%	9	100.0%	
Teaching experience * To transfer the intended meaning in order to avoid misunderstanding	9	100.0%	0	.0%	9	100.0%	
Teaching experience * To reinforce, emphasize or clarify messages that might not be understood	9	100.0%	0	.0%	9	100.0%	
Teaching experience * To respect others who are not fluent in either languages	9	100.0%	0	.0%	9	100.0%	
Teaching experience * I use more English in the workplace	9	100.0%	0	.0%	9	100.0%	
Teaching experience * I use more Malay language at home	9	100.0%	0	.0%	9	100.0%	
Teaching experience * I used both languages equally at the workplace and home	9	100.0%	0	.0%	9	100.0%	


Polytechnic * Why used both languages: I know both languages very well Crosstabulation	
Count	
		Why used both languages: I know both languages very well		
		Strongly agree	Agree	Total	
Polytechnic	PUO	2	1	3	
	PSA	2	0	2	
	PIS	3	1	4	
	Total	7	2	9	


Polytechnic * Just to show off that I know both languages Crosstabulation	
Count	
		Just to show off that I know both languages		
		Disagree	Strongly disagree	Total	
Polytechnic	PUO	0	3	3	
	PSA	2	0	2	
	PIS	2	2	4	
	Total	4	5	9	


Polytechnic * To show some western value/satus in myself Crosstabulation	
Count	
		To show some western value/satus in myself		
		Disagree	Strongly disagree	Total	
Polytechnic	PUO	0	3	3	
	PSA	2	0	2	
	PIS	2	2	4	
	Total	4	5	9	


Polytechnic * To create closeness among my colleagues and students Crosstabulation	
Count	
		To create closeness among my colleagues and students		
		Strongly agree	Agree	Disagree	Total	
Polytechnic	PUO	1	2	0	3	
	PSA	0	2	0	2	
	PIS	2	1	1	4	
	Total	3	5	1	9	


Polytechnic * To signal the change in topic and to get Ss' attention Crosstabulation	
Count	
		To signal the change in topic and to get Ss' attention		
		Strongly agree	Agree	Disagree	Total	
Polytechnic	PUO	2	1	0	3	
	PSA	0	1	1	2	
	PIS	3	1	0	4	
	Total	5	3	1	9	


Polytechnic * To build solidarity and intimate relations with the students Crosstabulation	
Count	
		To build solidarity and intimate relations with the students		
		Strongly agree	Agree	Disagree	Total	
Polytechnic	PUO	0	2	0	2	
	PSA	0	2	0	2	
	PIS	2	1	1	4	
	Total	2	5	1	8	


Polytechnic * To lower the language barrier that might have between myself and the others Crosstabulation	
Count	
		To lower the language barrier that might have between myself and the others		
		Strongly agree	Agree	Total	
Polytechnic	PUO	1	2	3	
	PSA	0	2	2	
	PIS	4	0	4	
	Total	5	4	9	


Polytechnic * To transfer the necessary knowledge to the students for clarity & comprehension Crosstabulation	
Count	
		To transfer the necessary knowledge to the students for clarity & comprehension		
		Strongly agree	Agree	Total	
Polytechnic	PUO	2	1	3	
	PSA	0	2	2	
	PIS	4	0	4	
	Total	6	3	9	


Polytechnic * To get Ss' attention & trust before proceeding to the teaching of concepts/theories esp. those who are weak Crosstabulation	
Count	
		To get Ss' attention & trust before proceeding to the teaching of concepts/theories esp. those who are weak		
		Strongly agree	Agree	Total	
Polytechnic	PUO	1	2	3	
	PSA	0	2	2	
	PIS	4	0	4	
	Total	5	4	9	


Polytechnic * To cover up my weaknesses in English language Crosstabulation	
Count	
		To cover up my weaknesses in English language		
		Strongly agree	Agree	Disagree	Strongly disagree	Total	
Polytechnic	PUO	0	0	0	3	3	
	PSA	0	0	1	1	2	
	PIS	1	1	0	2	4	
	Total	1	1	1	6	9	


Polytechnic * To transfer the intended meaning in order to avoid misunderstanding Crosstabulation	
Count	
		To transfer the intended meaning in order to avoid misunderstanding		
		Strongly agree	Agree	Total	
Polytechnic	PUO	3	0	3	
	PSA	0	2	2	
	PIS	4	0	4	
	Total	7	2	9	


Polytechnic * To reinforce, emphasize or clarify messages that might not be understood Crosstabulation	
Count	
		To reinforce, emphasize or clarify messages that might not be understood		
		Strongly agree	Agree	Total	
Polytechnic	PUO	3	0	3	
	PSA	0	2	2	
	PIS	4	0	4	
	Total	7	2	9	


Polytechnic * To respect others who are not fluent in either languages Crosstabulation	
Count	
		To respect others who are not fluent in either languages		
		Strongly agree	Agree	Total	
Polytechnic	PUO	0	3	3	
	PSA	0	2	2	
	PIS	2	2	4	
	Total	2	7	9	


Polytechnic * I use more English in the workplace Crosstabulation	
Count	
		I use more English in the workplace		
		Strongly agree	Agree	Disagree	Total	
Polytechnic	PUO	1	2	0	3	
	PSA	0	1	1	2	
	PIS	0	4	0	4	
	Total	1	7	1	9	


Polytechnic * I use more Malay language at home Crosstabulation	
Count	
		I use more Malay language at home		
		Strongly agree	Agree	Strongly disagree	Total	
Polytechnic	PUO	2	0	1	3	
	PSA	1	0	1	2	
	PIS	1	2	1	4	
	Total	4	2	3	9	


Polytechnic * I used both languages equally at the workplace and home Crosstabulation	
Count	
		I used both languages equally at the workplace and home		
		Agree	Disagree	Total	
Polytechnic	PUO	3	0	3	
	PSA	1	1	2	
	PIS	3	1	4	
	Total	7	2	9	


Gender * Why used both languages: I know both languages very well Crosstabulation	
Count	
		Why used both languages: I know both languages very well		
		Strongly agree	Agree	Total	
Gender	Male	3	0	3	
	Female	4	2	6	
	Total	7	2	9	


Gender * Just to show off that I know both languages Crosstabulation	
Count	
		Just to show off that I know both languages		
		Disagree	Strongly disagree	Total	
Gender	Male	2	1	3	
	Female	2	4	6	
	Total	4	5	9	


Gender * To show some western value/status in myself Crosstabulation	
Count	
		To show some western value/status in myself		
		Disagree	Strongly disagree	Total	
Gender	Male	2	1	3	
	Female	2	4	6	
	Total	4	5	9	


Gender * To create closeness among my colleagues and students Crosstabulation	
Count	
		To create closeness among my colleagues and students		
		Strongly agree	Agree	Disagree	Total	
Gender	Male	1	1	1	3	
	Female	2	4	0	6	
	Total	3	5	1	9	


Gender * To signal the change in topic and to get Ss' attention Crosstabulation	
Count	
		To signal the change in topic and to get Ss' attention		
		Strongly agree	Agree	Disagree	Total	
Gender	Male	2	1	0	3	
	Female	3	2	1	6	
	Total	5	3	1	9	


Gender * To build solidarity and intimate relations with the students Crosstabulation	
Count	
		To build solidarity and intimate relations with the students		
		Strongly agree	Agree	Disagree	Total	
Gender	Male	2	1	0	3	
	Female	0	4	1	5	
	Total	2	5	1	8	


Gender * To lower the language barrier that might have between myself and the others Crosstabulation	
Count	
		To lower the language barrier that might have between myself and the others		
		Strongly agree	Agree	Total	
Gender	Male	2	1	3	
	Female	3	3	6	
	Total	5	4	9	


Gender * To transfer the necessary knowledge to the students for clarity & comprehension Crosstabulation	
Count	
		To transfer the necessary knowledge to the students for clarity & comprehension		
		Strongly agree	Agree	Total	
Gender	Male	2	1	3	
	Female	4	2	6	
	Total	6	3	9	


Gender * To get Ss' attention & trust before proceeding to the teaching of concepts/theories esp. those who are weak Crosstabulation	
Count	
		To get Ss' attention & trust before proceeding to the teaching of concepts/theories esp. those who are weak		
		Strongly agree	Agree	Total	
Gender	Male	2	1	3	
	Female	3	3	6	
	Total	5	4	9	


Gender * To cover up my weaknesses in English language Crosstabulation	
Count	
		To cover up my weaknesses in English language		
		Strongly agree	Agree	Disagree	Strongly disagree	Total	
Gender	Male	0	1	1	1	3	
	Female	1	0	0	5	6	
	Total	1	1	1	6	9	


Gender * To transfer the intended meaning in order to avoid misunderstanding Crosstabulation	
Count	
		To transfer the intended meaning in order to avoid misunderstanding		
		Strongly agree	Agree	Total	
Gender	Male	2	1	3	
	Female	5	1	6	
	Total	7	2	9	


Gender * To reinforce, emphasize or clarify messages that might not be understood Crosstabulation	
Count	
		To reinforce, emphasize or clarify messages that might not be understood		
		Strongly agree	Agree	Total	
Gender	Male	2	1	3	
	Female	5	1	6	
	Total	7	2	9	


Gender * To respect others who are not fluent in either languages Crosstabulation	
Count	
		To respect others who are not fluent in either languages		
		Strongly agree	Agree	Total	
Gender	Male	1	2	3	
	Female	1	5	6	
	Total	2	7	9	


Gender * I use more English in the workplace Crosstabulation	
Count	
		I use more English in the workplace		
		Strongly agree	Agree	Disagree	Total	
Gender	Male	0	2	1	3	
	Female	1	5	0	6	
	Total	1	7	1	9	


Gender * I use more Malay language at home Crosstabulation	
Count	
		I use more Malay language at home		
		Strongly agree	Agree	Strongly disagree	Total	
Gender	Male	2	1	0	3	
	Female	2	1	3	6	
	Total	4	2	3	9	


Gender * I used both languages equally at the workplace and home Crosstabulation	
Count	
		I used both languages equally at the workplace and home		
		Agree	Disagree	Total	
Gender	Male	2	1	3	
	Female	5	1	6	
	Total	7	2	9	


Teaching experience * Why used both languages: I know both languages very well Crosstabulation	
Count	
		Why used both languages: I know both languages very well		
		Strongly agree	Agree	Total	
Teaching experience	0-5	2	2	4	
	6-10	1	0	1	
	11-15	1	0	1	
	21-25	1	0	1	
	26-30	1	0	1	
	31-35	1	0	1	
	Total	7	2	9	


Teaching experience * Just to show off that I know both languages Crosstabulation	
Count	
		Just to show off that I know both languages		
		Disagree	Strongly disagree	Total	
Teaching experience	0-5	2	2	4	
	6-10	1	0	1	
	11-15	0	1	1	
	21-25	1	0	1	
	26-30	0	1	1	
	31-35	0	1	1	
	Total	4	5	9	


Teaching experience * To show some western value/status in myself Crosstabulation	
Count	
		To show some western value/status in myself		
		Disagree	Strongly disagree	Total	
Teaching experience	0-5	2	2	4	
	6-10	1	0	1	
	11-15	0	1	1	
	21-25	1	0	1	
	26-30	0	1	1	
	31-35	0	1	1	
	Total	4	5	9	


Teaching experience * To create closeness among my colleagues and students Crosstabulation	
Count	
		To create closeness among my colleagues and students		
		Strongly agree	Agree	Disagree	Total	
Teaching experience	0-5	2	2	0	4	
	6-10	0	1	0	1	
	11-15	0	1	0	1	
	21-25	0	1	0	1	
	26-30	1	0	0	1	
	31-35	0	0	1	1	
	Total	3	5	1	9	


Teaching experience * To signal the change in topic and to get Ss' attention Crosstabulation	
Count	
		To signal the change in topic and to get Ss' attention		
		Strongly agree	Agree	Disagree	Total	
Teaching experience	0-5	2	2	0	4	
	6-10	0	0	1	1	
	11-15	1	0	0	1	
	21-25	0	1	0	1	
	26-30	1	0	0	1	
	31-35	1	0	0	1	
	Total	5	3	1	9	


Teaching experience * To build solidarity and intimate relations with the students Crosstabulation	
Count	
		To build solidarity and intimate relations with the students		
		Strongly agree	Agree	Disagree	Total	
Teaching experience	0-5	1	2	1	4	
	6-10	0	1	0	1	
	11-15	0	1	0	1	
	21-25	0	1	0	1	
	31-35	1	0	0	1	
	Total	2	5	1	8	


Teaching experience * To lower the language barrier that might have between myself and the others Crosstabulation	
Count	
		To lower the language barrier that might have between myself and the others		
		Strongly agree	Agree	Total	
Teaching experience	0-5	3	1	4	
	6-10	0	1	1	
	11-15	0	1	1	
	21-25	0	1	1	
	26-30	1	0	1	
	31-35	1	0	1	
	Total	5	4	9	


Teaching experience * To transfer the necessary knowledge to the students for clarity & comprehension Crosstabulation	
Count	
		To transfer the necessary knowledge to the students for clarity & comprehension		
		Strongly agree	Agree	Total	
Teaching experience	0-5	4	0	4	
	6-10	0	1	1	
	11-15	0	1	1	
	21-25	0	1	1	
	26-30	1	0	1	
	31-35	1	0	1	
	Total	6	3	9	


Teaching experience * To get Ss' attention & trust before proceeding to the teaching of concepts/theories esp. those who are weak Crosstabulation	
Count	
		To get Ss' attention & trust before proceeding to the teaching of concepts/theories esp. those who are weak		
		Strongly agree	Agree	Total	
Teaching experience	0-5	4	0	4	
	6-10	0	1	1	
	11-15	0	1	1	
	21-25	0	1	1	
	26-30	0	1	1	
	31-35	1	0	1	
	Total	5	4	9	


Teaching experience * To cover up my weaknesses in English language Crosstabulation	
Count	
		To cover up my weaknesses in English language		
		Strongly agree	Agree	Disagree	Strongly disagree	Total	
Teaching experience	0-5	1	1	0	2	4	
	6-10	0	0	0	1	1	
	11-15	0	0	0	1	1	
	21-25	0	0	1	0	1	
	26-30	0	0	0	1	1	
	31-35	0	0	0	1	1	
	Total	1	1	1	6	9	


Teaching experience * To transfer the intended meaning in order to avoid misunderstanding Crosstabulation	
Count	
		To transfer the intended meaning in order to avoid misunderstanding		
		Strongly agree	Agree	Total	
Teaching experience	0-5	4	0	4	
	6-10	0	1	1	
	11-15	1	0	1	
	21-25	0	1	1	
	26-30	1	0	1	
	31-35	1	0	1	
	Total	7	2	9	


Teaching experience * To reinforce, emphasize or clarify messages that might not be understood Crosstabulation	
Count	
		To reinforce, emphasize or clarify messages that might not be understood		
		Strongly agree	Agree	Total	
Teaching experience	0-5	4	0	4	
	6-10	0	1	1	
	11-15	1	0	1	
	21-25	0	1	1	
	26-30	1	0	1	
	31-35	1	0	1	
	Total	7	2	9	


Teaching experience * To respect others who are not fluent in either languages Crosstabulation	
Count	
		To respect others who are not fluent in either languages		
		Strongly agree	Agree	Total	
Teaching experience	0-5	1	3	4	
	6-10	0	1	1	
	11-15	0	1	1	
	21-25	0	1	1	
	26-30	0	1	1	
	31-35	1	0	1	
	Total	2	7	9	


Teaching experience * I use more English in the workplace Crosstabulation	
Count	
		I use more English in the workplace		
		Strongly agree	Agree	Disagree	Total	
Teaching experience	0-5	0	4	0	4	
	6-10	0	1	0	1	
	11-15	0	1	0	1	
	21-25	0	0	1	1	
	26-30	1	0	0	1	
	31-35	0	1	0	1	
	Total	1	7	1	9	


Teaching experience * I use more Malay language at home Crosstabulation	
Count	
		I use more Malay language at home		
		Strongly agree	Agree	Strongly disagree	Total	
Teaching experience	0-5	1	2	1	4	
	6-10	0	0	1	1	
	11-15	1	0	0	1	
	21-25	1	0	0	1	
	26-30	0	0	1	1	
	31-35	1	0	0	1	
	Total	4	2	3	9	


Teaching experience * I used both languages equally at the workplace and home Crosstabulation	
Count	
		I used both languages equally at the workplace and home		
		Agree	Disagree	Total	
Teaching experience	0-5	4	0	4	
	6-10	0	1	1	
	11-15	1	0	1	
	21-25	1	0	1	
	26-30	1	0	1	
	31-35	0	1	1	
	Total	7	2	9	


[DataSet1] C:\Users\Mazlin\Desktop\Lecturers' survey.sav


Case Processing Summary	
	Cases	
	Valid	Missing	Total	
	N	Percent	N	Percent	N	Percent	
Polytechnic * Do you like teaching in English or Malay or both?	9	100.0%	0	.0%	9	100.0%	
Polytechnic * Why?	9	100.0%	0	.0%	9	100.0%	
Polytechnic * Do you like to make joke?	9	100.0%	0	.0%	9	100.0%	
Polytechnic * If yes, in what language?	9	100.0%	0	.0%	9	100.0%	
Polytechnic * Why?	8	88.9%	1	11.1%	9	100.0%	
Polytechnic * Ss will understand better in English or Malay?	9	100.0%	0	.0%	9	100.0%	
Polytechnic * Why?	9	100.0%	0	.0%	9	100.0%	
Polytechnic * What do you do to ensure Ss' understanding if using English in class?	9	100.0%	0	.0%	9	100.0%	
Polytechnic * Do you achieve your objective(s) in the lesson?	9	100.0%	0	.0%	9	100.0%	
Polytechnic * What improvement?	9	100.0%	0	.0%	9	100.0%	
Gender * Do you like teaching in English or Malay or both?	9	100.0%	0	.0%	9	100.0%	
Gender * Why?	9	100.0%	0	.0%	9	100.0%	
Gender * Do you like to make joke?	9	100.0%	0	.0%	9	100.0%	
Gender * If yes, in what language?	9	100.0%	0	.0%	9	100.0%	
Gender * Why?	8	88.9%	1	11.1%	9	100.0%	
Gender * Ss will understand better in English or Malay?	9	100.0%	0	.0%	9	100.0%	
Gender * Why?	9	100.0%	0	.0%	9	100.0%	
Gender * What do you you do to ensure Ss' understanding if using English in class?	9	100.0%	0	.0%	9	100.0%	
Gender * Do you achieve your objective(s) in the lesson?	9	100.0%	0	.0%	9	100.0%	
Gender * What improvement?	9	100.0%	0	.0%	9	100.0%	
Teaching experience * Do you like teaching in English or Malay or both?	9	100.0%	0	.0%	9	100.0%	
Teaching experience * Why?	9	100.0%	0	.0%	9	100.0%	
Teaching experience * Do you like to make joke?	9	100.0%	0	.0%	9	100.0%	
Teaching experience * If yes, in what language?	9	100.0%	0	.0%	9	100.0%	
Teaching experience * Why?	8	88.9%	1	11.1%	9	100.0%	
Teaching experience * Ss will understand better in English or Malay?	9	100.0%	0	.0%	9	100.0%	
Teaching experience * Why?	9	100.0%	0	.0%	9	100.0%	
Teaching experience * What do you do to ensure Ss' understanding if using English in class?	9	100.0%	0	.0%	9	100.0%	
Teaching experience * Do you achieve your objective(s) in the lesson?	9	100.0%	0	.0%	9	100.0%	
Teaching experience * What improvement?	9	100.0%	0	.0%	9	100.0%	


Polytechnic * Do you like teaching in English or Malay or both? Crosstabulation	
Count	
		Do you like teaching in English or Malay or both?		
		English only	Both	Total	
Polytechnic	PUO	3	0	3	
	PSA	0	2	2	
	PIS	1	3	4	
	Total	4	5	9	


Polytechnic * Why? Crosstabulation	
Count	
		Why?	
		As part of my working requirement and also to develop English language environment among the students	English only because I seldom use Malay aprt from just using it to speak with people who can't speak English. I am not used to using it to teach any academic subject.	In order to build students interest to speak it, have to start with the lecturer	I'm comfortable and have no problem using both languages	Sometime students' level of the English language competency is below par, so I need to explain in the native language so that they understand better	
Polytechnic	PUO	1	1	1	0	0	
	PSA	0	0	0	1	1	
	PIS	0	0	0	0	0	
	Total	1	1	1	1	1	

Polytechnic * Why? Crosstabulation	
Count	
		Why?		
		To make it understandable to some students. Some of them won't bother me teaching in class at all because they don't understand.	I try to speak only in English, repeat words in Malay after it is mentioned by the students	I believe teaching English in both languages can help my students to learn because they have different proficiency in English.	Fluent in both	Total	
Polytechnic	PUO	0	0	0	0	3	
	PSA	0	0	0	0	2	
	PIS	1	1	1	1	4	
	Total	1	1	1	1	9	


Polytechnic * Do you like to make joke? Crosstabulation	
Count	
		Do you like to make joke?		
		Yes	Total	
Polytechnic	PUO	3	3	
	PSA	2	2	
	PIS	4	4	
	Total	9	9	


Polytechnic * If yes, in what language? Crosstabulation	
Count	
		If yes, in what language?		
		English	Malay	Both	Total	
Polytechnic	PUO	0	1	2	3	
	PSA	1	0	1	2	
	PIS	1	1	2	4	
	Total	2	2	5	9	


Polytechnic * Why? Crosstabulation	
Count	
		Why?	
		Too attract their interests	It depends on the person I am with. I only use Malay if the joke sounds better if it is made in the Malay and I can't translate it into English.	Some students might not get the joke if it's in English	Depends on the situation. Before class start, I use Malay to have a small chat with the students. Just to create the mood.	I try to get the students to understand the joke in English	
Polytechnic	PUO	1	1	1	0	0	
	PSA	0	0	1	0	0	
	PIS	0	0	0	1	1	
	Total	1	1	2	1	1	

Polytechnic * Why? Crosstabulation	
Count	
		Why?		
		This is due to certain jokes are hard to tell in either language so it depends on the jokes itself	Students understand better & appropriately respond	Total	
Polytechnic	PUO	0	0	3	
	PSA	0	0	1	
	PIS	1	1	4	
	Total	1	1	8	


Polytechnic * Ss will understand better in English or Malay? Crosstabulation	
Count	
		Ss will understand better in English or Malay?		
		English	Malay	Both	Total	
Polytechnic	PUO	3	0	0	3	
	PSA	0	2	0	2	
	PIS	0	3	1	4	
	Total	3	5	1	9	


Polytechnic * Why? Crosstabulation	
Count	
		Why?	
		English-Specifically for this class, the students could understand me better in English because they have the proficiency	English-Most students can understand English. Only a few have difficulties	English-Students will be able to understand me in English if I use simple work & language to explain anything but of course repeating the same thing in Malay can help them	Malay-Because of their level of English is just average	Malay-Because their mother tongue's language is dominant than the other, but I always try to maximise my usage of English language/understand better in their mother tongue and limit the usage of native language when necessary	
Polytechnic	PUO	1	1	1	0	0	
	PSA	0	0	0	1	1	
	PIS	0	0	0	0	2	
	Total	1	1	1	1	3	

Polytechnic * Why? Crosstabulation	
Count	
		Why?		
		Both-We have a mutual understanding of using both languages in class, without complaining it to anyone.	Malay-because it is their mother tongue but if I do teach frequently in Malay, the need to converse in English is less	Total	
Polytechnic	PUO	0	0	3	
	PSA	0	0	2	
	PIS	1	1	4	
	Total	1	1	9	


Polytechnic * What do you you do to ensure Ss' understanding if using English in class? Crosstabulation	
Count	
		What do you you do to ensure Ss' understanding if using English in class?	
		Asking the students to rephrase their understanding of the topic discussed	I would use Malay to explain the part(s) they don't understand.	Ask them to repeat the information given	Explain further in English but describing slowly and with the help from dictionary	I check them by asking question like 'do you follow me?' or 'am I going too fast' or please stop me if you need to ask question when you don't understand.'	
Polytechnic	PUO	1	1	1	0	0	
	PSA	0	0	0	1	1	
	PIS	0	0	0	0	0	
	Total	1	1	1	1	1	

Polytechnic * What do you you do to ensure Ss' understanding if using English in class? Crosstabulation	
Count	
		What do you do to ensure Ss' understanding if using English in class?		
		Asking and get back to the weak students later. They're usually shy to be connected or called in class.	I ask them to tell me what they understand in the native language i.e. BM	I will give more examples and give some description in a simple language/word choice that they are familiar	Explain in depth about the content	Total	
Polytechnic	PUO	0	0	0	0	3	
	PSA	0	0	0	0	2	
	PIS	1	1	1	1	4	
	Total	1	1	1	1	9	


Polytechnic * Do you achieve your objective(s) in the lesson? Crosstabulation	
Count	
		Do you achieve your objective(s) in the lesson?		
		Achieved	Partially achieved	Total	
Polytechnic	PUO	3	0	3	
	PSA	1	1	2	
	PIS	4	0	4	
	Total	8	1	9	


Polytechnic * What improvement? Crosstabulation	
Count	
		What improvement?	
		Students would use English not only during the lesson but apply the same knowledge and askills in the real situation, therefore I would require them to use the specific language focus when they discuss in other topic	To provide more examples for each section of the cover letter.	More groupworks for the students & focus on their writing	To have extra hours of discussion and more exercises to be given to them	Increase students' enthusiasm in the language. Make them feel t ease with the language to build up their confidence.	
Polytechnic	PUO	1	1	1	0	0	
	PSA	0	0	0	1	1	
	PIS	0	0	0	0	0	
	Total	1	1	1	1	1	

Polytechnic * What improvement? Crosstabulation	
Count	
		What improvement?		
		Rather than the subject, I think I need help on technical stuffs. Reading materials too should be a good thing to share with students, but then they might giving up half way reading because they find it troublesome to understand the reading materials.	I am planning to incorporate more visual, AVA, when teaching. Encourage students to speak in English, learn "how to learn" the language rather than treating it as a subject to pass.	I think I should provide more examples and we could do a simple role-play about preparation of job interview	Guide students to use more English even during group discussion	Total	
Polytechnic	PUO	0	0	0	0	3	
	PSA	0	0	0	0	2	
	PIS	1	1	1	1	4	
	Total	1	1	1	1	9	


Gender * Do you like teaching in English or Malay or both? Crosstabulation	
Count	
		Do you like teaching in English or Malay or both?		
		English only	Both	Total	
Gender	Male	0	3	3	
	Female	4	2	6	
	Total	4	5	9	


Gender * Why? Crosstabulation	
Count	
		Why?	
		As part of my working requirement and also to develop English language environment among the students	English only because I seldom use Malay apart from just using it to speak with people who can't speak English. I am not used to using it to teach any academic subject.	In order to build students interest to speak it, have to start with the lecturer	I'm comfortable and have no problem using both languages	Sometime students' level of the English language competency is below par, so I need to explain in the native language so that they understand better	
Gender	Male	0	0	0	0	1	
	Female	1	1	1	1	0	
	Total	1	1	1	1	1	

Gender * Why? Crosstabulation	
Count	
		Why?		
		To make it understandable to some students. Some of them won't bother me teaching in class at all because they don't understand.	I try to speak only in English, repeat words in Malay after it is mentioned by the students	I believe teaching English in both languages can help my students to learn because they have different proficiency in English.	Fluent in both	Total	
Gender	Male	0	0	1	1	3	
	Female	1	1	0	0	6	
	Total	1	1	1	1	9	


Gender * Do you like to make joke? Crosstabulation	
Count	
		Do you like to make joke?		
		Yes	Total	
Gender	Male	3	3	
	Female	6	6	
	Total	9	9	


Gender * If yes, in what language? Crosstabulation	
Count	
		If yes, in what language?		
		English	Malay	Both	Total	
Gender	Male	0	1	2	3	
	Female	2	1	3	6	
	Total	2	2	5	9	


Gender * Why? Crosstabulation	
Count	
		Why?	
		Too attract their interests	It depends on the person I am with. I only use Malay if the joke sounds better if it is made in the Malay and I can't translate it into English.	Some students might not get the joke if it's in English	Depends on the situation. Before class start, I use Malay to have a small chat with the students. Just to create the mood.	I try to get the students to understand the joke in English	
Gender	Male	0	0	1	0	0	
	Female	1	1	1	1	1	
	Total	1	1	2	1	1	

Gender * Why? Crosstabulation	
Count	
		Why?		
		This is due to certain jokes are hard to tell in either language so it depends on the jokes itself	Students understand better & appropriately respond	Total	
Gender	Male	1	1	3	
	Female	0	0	5	
	Total	1	1	8	


Gender * Ss will understand better in English or Malay? Crosstabulation	
Count	
		Ss will understand better in English or Malay?		
		English	Malay	Both	Total	
Gender	Male	0	3	0	3	
	Female	3	2	1	6	
	Total	3	5	1	9	


Gender * Why? Crosstabulation	
Count	
		Why?	
		English-Specifically for this class, the students could understand me better in English because they have the proficiency	English-Most students can understand English. Only a few have difficulties	English-Students will be able to understand me in English if I use simple work & language to explain anything but of course repeating the same thing in Malay can help them	Malay-Because of their level of English is just average	Malay-Because their mother tongue's language is dominant than the other, but I always try to maximise my usage of English language/understand better in their mother tongue and limit the usage of native language when necessary	
Gender	Male	0	0	0	0	3	
	Female	1	1	1	1	0	
	Total	1	1	1	1	3	

Gender * Why? Crosstabulation	
Count	
		Why?		
		Both-We have a mutual understanding of using both languages in class, without complaining it to anyone.	Malay-because it is their mother tongue but f I do teach frequently in Malay, the need to converse in English is less	Total	
Gender	Male	0	0	3	
	Female	1	1	6	
	Total	1	1	9	


Gender * What do you you do to ensure Ss' understanding if using English in class? Crosstabulation	
Count	
		What do you do to ensure Ss' understanding if using English in class?	
		Asking the students to rephrase their understanding of the topic discussed	I would use Malay to explain the part(s) they don't understand.	Ask them to repeat the information given	Explain further in English but describing slowly and with the help from dictionary	I check them by asking question like 'do you follow me?' or 'am I going too fast' or please stop me if you need to ask question when you don't understand.'	
Gender	Male	0	0	0	0	1	
	Female	1	1	1	1	0	
	Total	1	1	1	1	1	

Gender * What do you you do to ensure Ss' understanding if using English in class? Crosstabulation	
Count	
		What do you you do to ensure Ss' understanding if using English in class?		
		Asking and get back to the weak students later. They're usually shy to be connected or called in class.	I ask them to tell me what they understand in the native language i.e. BM	I will give more examples and give some description in a simple language/word choice that they are familiar	Explain in depth about the content	Total	
Gender	Male	0	0	1	1	3	
	Female	1	1	0	0	6	
	Total	1	1	1	1	9	


Gender * Do you achieve your objective(s) in the lesson? Crosstabulation	
Count	
		Do you achieve your objective(s) in the lesson?		
		Achieved	Partially achieved	Total	
Gender	Male	3	0	3	
	Female	5	1	6	
	Total	8	1	9	


Gender * What improvement? Crosstabulation	
Count	
		What improvement?	
		Students would use English not only during the lesson but apply the same knowledge and skills in the real situation, therefore I would require them to use the specific language focus when they discuss in other topic	To provide more examples for each section of the cover letter.	More groupworks for the students & focus on their writing	To have extra hours of discussion and more exercises to be given to them	Increase students' enthusiasm in the language. Make them feel t ease with the language to build up their confidence.	
Gender	Male	0	0	0	0	1	
	Female	1	1	1	1	0	
	Total	1	1	1	1	1	

Gender * What improvement? Crosstabulation	
Count	
		What improvement?		
		Rather than the subject, I think I need help on technical stuffs. Reading materials too should be a good thing to share with students, but then they might giving up half way reading because they find it troublesome to understand the reading materials.	I am planning to incorporate more visual, AVA, when teaching. Encourage students to speak in English, learn "how to learn" the language rather than treating it as a subject to pass.	I think I should provide more examples and we could do a simple role-play about preparation of job interview	Guide students to use more English even during group discussion	Total	
Gender	Male	0	0	1	1	3	
	Female	1	1	0	0	6	
	Total	1	1	1	1	9	


Teaching experience * Do you like teaching in English or Malay or both? Crosstabulation	
Count	
		Do you like teaching in English or Malay or both?		
		English only	Both	Total	
Teaching experience	0-5	2	2	4	
	6-10	0	1	1	
	11-15	1	0	1	
	21-25	0	1	1	
	26-30	1	0	1	
	31-35	0	1	1	
	Total	4	5	9	


Teaching experience * Why? Crosstabulation	
Count	
		Why?	
		As part of my working requirement and also to develop English language environment among the students	English only because I seldom use Malay aprt from just using it to speak with people who can't speak English. I am not used to using it to teach any academic subject.	In order to build students interest to speak it, have to start with the lecturer	I'm comfortable and have no problem using both languages	
Teaching experience	0-5	0	0	1	0	
	6-10	0	0	0	1	
	11-15	1	0	0	0	
	21-25	0	0	0	0	
	26-30	0	1	0	0	
	31-35	0	0	0	0	
	Total	1	1	1	1	

Teaching experience * Why? Crosstabulation	
Count	
		Why?	
		Sometime students' level of the English language competency is below par, so I need to explain in the native language so that they understand better	To make it understandable to some students. Some of them won't bother me teaching in class at all because they don't understand.	I try to speak only in English, repeat words in Malay after it is mentioned by the students	I believe teaching English in both languages can help my students to learn because they have different proficiency in English.	
Teaching experience	0-5	0	1	1	1	
	6-10	0	0	0	0	
	11-15	0	0	0	0	
	21-25	1	0	0	0	
	26-30	0	0	0	0	
	31-35	0	0	0	0	
	Total	1	1	1	1	

Teaching experience * Why? Crosstabulation	
Count	
		Why?		
		Fluent in both	Total	
Teaching experience	0-5	0	4	
	6-10	0	1	
	11-15	0	1	
	21-25	0	1	
	26-30	0	1	
	31-35	1	1	
	Total	1	9	


Teaching experience * Do you like to make joke? Crosstabulation	
Count	
		Do you like to make joke?		
		Yes	Total	
Teaching experience	0-5	4	4	
	6-10	1	1	
	11-15	1	1	
	21-25	1	1	
	26-30	1	1	
	31-35	1	1	
	Total	9	9	


Teaching experience * If yes, in what language? Crosstabulation	
Count	
		If yes, in what language?		
		English	Malay	Both	Total	
Teaching experience	0-5	1	1	2	4	
	6-10	1	0	0	1	
	11-15	0	0	1	1	
	21-25	0	0	1	1	
	26-30	0	0	1	1	
	31-35	0	1	0	1	
	Total	2	2	5	9	


Teaching experience * Why? Crosstabulation	
Count	
		Why?	
		Too attract their interests	It depends on the person I am with. I only use Malay if the joke sounds better if it is made in the Malay and I can't translate it into English.	Some students might not get the joke if it's in English	Depends on the situation. Before class start, I use Malay to have a small chat with the students. Just to create the mood.	
Teaching experience	0-5	0	0	1	1	
	11-15	1	0	0	0	
	21-25	0	0	1	0	
	26-30	0	1	0	0	
	31-35	0	0	0	0	
	Total	1	1	2	1	

Teaching experience * Why? Crosstabulation	
Count	
		Why?		
		I try to get the students to understand the joke in English	This is due to certain jokes are hard to tell in either language so it depends on the jokes itself	Students understand better & appropriately respond	Total	
Teaching experience	0-5	1	1	0	4	
	11-15	0	0	0	1	
	21-25	0	0	0	1	
	26-30	0	0	0	1	
	31-35	0	0	1	1	
	Total	1	1	1	8	


Teaching experience * Ss will understand better in English or Malay? Crosstabulation	
Count	
		Ss will understand better in English or Malay?		
		English	Malay	Both	Total	
Teaching experience	0-5	1	2	1	4	
	6-10	0	1	0	1	
	11-15	1	0	0	1	
	21-25	0	1	0	1	
	26-30	1	0	0	1	
	31-35	0	1	0	1	
	Total	3	5	1	9	


Teaching experience * Why? Crosstabulation	
Count	
		Why?	
		English-Specifically for this class, the students could understand me better in English because they have the proficiency	English-Most students can understand English. Only a few have difficulties	English-Students will be able to understand me in English if I use simple work & language to explain anything but of course repeating the same thing in Malay can help them	Malay-Because of their level of English is just average	
Teaching experience	0-5	0	0	1	0	
	6-10	0	0	0	1	
	11-15	1	0	0	0	
	21-25	0	0	0	0	
	26-30	0	1	0	0	
	31-35	0	0	0	0	
	Total	1	1	1	1	

Teaching experience * Why? Crosstabulation	
Count	
		Why?		
		Malay-Because their mother tongue's language is dominant than the other, but I always try to maximise my usage of English language/understand better in their mother tongue and limit the usage of native language when necessary	Both-We have a mutual understanding of using both languages in class, without complaining it to anyone.	Malay-because it is their mother tongue but f I do teach frequently in Malay, the need to converse in English is less	Total	
Teaching experience	0-5	1	1	1	4	
	6-10	0	0	0	1	
	11-15	0	0	0	1	
	21-25	1	0	0	1	
	26-30	0	0	0	1	
	31-35	1	0	0	1	
	Total	3	1	1	9	


Teaching experience * What do you do to ensure Ss' understanding if using English in class? Crosstabulation	
Count	
		What do you do to ensure Ss' understanding if using English in class?	
		Asking the students to rephrase their understanding of the topic discussed	I would use Malay to explain the part(s) they don't understand.	Ask them to repeat the information given	Explain further in English but describing slowly and with the help from dictionary	
Teaching experience	0-5	0	0	1	0	
	6-10	0	0	0	1	
	11-15	1	0	0	0	
	21-25	0	0	0	0	
	26-30	0	1	0	0	
	31-35	0	0	0	0	
	Total	1	1	1	1	

Teaching experience * What do you do to ensure Ss' understanding if using English in class? Crosstabulation	
Count	
		What do you do to ensure Ss' understanding if using English in class?	
		I check them by asking question like 'do you follow me?' or 'am I going too fast' or please stop me if you need to ask question when you don't understand.'	Asking and get back to the weak students later. They're usually shy to be connected or called in class.	I ask them to tell me what they understand in the native language i.e. BM	I will give more examples and give some description in a simple language/word choice that they are familiar	
Teaching experience	0-5	0	1	1	1	
	6-10	0	0	0	0	
	11-15	0	0	0	0	
	21-25	1	0	0	0	
	26-30	0	0	0	0	
	31-35	0	0	0	0	
	Total	1	1	1	1	

Teaching experience * What do you do to ensure Ss' understanding if using English in class? Crosstabulation	
Count	
		What do you do to ensure Ss' understanding if using English in class?		
		Explain in depth about the content	Total	
Teaching experience	0-5	0	4	
	6-10	0	1	
	11-15	0	1	
	21-25	0	1	
	26-30	0	1	
	31-35	1	1	
	Total	1	9	


Teaching experience * Do you achieve your objective(s) in the lesson? Crosstabulation	
Count	
		Do you achieve your objective(s) in the lesson?		
		Achieved	Partially achieved	Total	
Teaching experience	0-5	4	0	4	
	6-10	0	1	1	
	11-15	1	0	1	
	21-25	1	0	1	
	26-30	1	0	1	
	31-35	1	0	1	
	Total	8	1	9	


Teaching experience * What improvement? Crosstabulation	
Count	
		What improvement?	
		Students would use English not only during the lesson but apply the same knowledge and skills in the real situation, therefore I would require them to use the specific language focus when they discuss in other topic	To provide more examples for each section of the cover letter.	More groupworks for the students & focus on their writing	To have extra hours of discussion and more exercises to be given to them	
Teaching experience	0-5	0	0	1	0	
	6-10	0	0	0	1	
	11-15	1	0	0	0	
	21-25	0	0	0	0	
	26-30	0	1	0	0	
	31-35	0	0	0	0	
	Total	1	1	1	1	

Teaching experience * What improvement? Crosstabulation	
Count	
		What improvement?	
		Increase students' enthusiasm in the language. Make them feel t ease with the language to build up their confidence.	Rather than the subject, I think I need help on technical stuffs. Reading materials too should be a good thing to share with students, but then they might giving up half way reading because they find it troublesome to understand the reading materials.	I am planning to incorporate more visual, AVA, when teaching. Encourage students to speak in English, learn "how to learn" the language rather than treating it as a subject to pass.	I think I should provide more examples and we could do a simple role-play about preparation of job interview	
Teaching experience	0-5	0	1	1	1	
	6-10	0	0	0	0	
	11-15	0	0	0	0	
	21-25	1	0	0	0	
	26-30	0	0	0	0	
	31-35	0	0	0	0	
	Total	1	1	1	1	

Teaching experience * What improvement? Crosstabulation	
Count	
		What improvement?		
		Guide students to use more English even during group discussion	Total	
Teaching experience	0-5	0	4	
	6-10	0	1	
	11-15	0	1	
	21-25	0	1	
	26-30	0	1	
	31-35	1	1	
	Total	1	9	
